# Distinct time trends in colorectal cancer incidence in countries with SDI levels from 1990 to 2019: an age–period–cohort analysis for the Global Burden of Disease 2019 study

**DOI:** 10.3389/fpubh.2024.1370282

**Published:** 2024-05-22

**Authors:** Yan Zhang, Xun-Bing Zhang, Yu-Wei Ding, Yang Kong, Xiao-Feng Zhu, Pu-Heng Li, Yang Tian, Qing-Wei Zhang

**Affiliations:** ^1^Department of Medical Oncology, Cancer Institute, Key Laboratory of Cancer Prevention and Intervention, Ministry of Education, The Second Affiliated Hospital of Zhejiang University School of Medicine, Hangzhou, Zhejiang, China; ^2^Department of Digestive Endoscopy Center, Shuguang Hospital Affiliated to Shanghai University of Traditional Chinese Medicine, Shanghai, China; ^3^Department of Hepatobiliary and Pancreatic Surgery, The Second Affiliated Hospital of Zhejiang University School of Medicine, Hangzhou, Zhejiang, China; ^4^Department of Hepatobiliary and Pancreatic Surgery, Huzhou Central Hospital, Huzhou, Zhejiang, China; ^5^School of Mathematical Sciences, Peking University, Beijing, China; ^6^Division of Gastroenterology and Hepatology, NHC Key Laboratory of Digestive Diseases, Shanghai Institute of Digestive Disease, Renji Hospital, School of Medicine, Shanghai Jiao Tong University, Shanghai, China

**Keywords:** incidence, age-period-cohort analysis, colorectal cancer, SDI, public health

## Abstract

**Introduction:**

The burden of colorectal cancer (CRC) plays a pivotal role in the global cancer epidemic. Our study reported the incidence trends in CRC and the associated effects of age, period, and birth cohort in 204 countries and territories over the past 30 years.

**Methods:**

The incidence data of CRC were extracted from the Global Burden of Disease Study (GBD) 2019. We performed the age–period–cohort (APC) model to estimate the overall annual percentage change (net drift) in the incidence rate, the annual percentage change by age group (local drift), and the relative risk (period and cohort effects) of the period and cohort in CRC during 1990–2019. This approach allows examining and distinguishing age, period, and cohort effects in incidence and potentially distinguishing colorectal cancer gaps in prevention and screening.

**Results:**

In 2019, the incidence of CRC was 2.17 (95% UI 2.00–2.34) million, of which China, the United States of America, and Japan had the highest incidence population, accounting for 45.9% of the global population. The age–standardized incidence rate (ASIR) was 26.7 (95% UI 28.9–24.6) per 100,000 people, of which 30 countries had an incidence rate greater than 40.0 per 100,000 people. From 1990 to 2019, the middle SDI region had the largest increase in incidence rate, with a net drift of 2.33% (95% CI 2.2–2.46%, *p* < 0.001). Globally, the incidence population was concentrated in the age group of 50–69 years, and the age group of 30–34 years had the largest increase in incidence rate (local drift 1.19% (95% CI 1.01–1.37%)). At the same time, the sex and age distributions of CRC incidence had significant heterogeneity across regions and countries. In the past 30 years, the incidence rate in 31 countries has been well controlled (net drift <0), and most of them were concentrated in high–and high–middle–SDI regions, such as Australia, Czechia, and Belgium, and the relative risk of incidence generally improved over time and consecutive young birth cohorts. CRC incidence showed an unfavorable trend (net drift ≥1%) in 89 countries, of which 27 countries were more significant (net drift >2%), mostly concentrated in the middle SDI region, such as China, Mexico, and Brazil, and the risk of period and birth cohort was unfavorable.

**Conclusion:**

Globally, the incidence of CRC has shown an overall upward trend over the past 30 years, with the exception of some countries with higher SDI values. Significant age–period–cohort differences were observed in the risk of incidence in CRC worldwide. Effective prevention and control policies need to take into account the age–period–cohort effect characteristics of different regions.

## Introduction

Currently, colorectal cancer (CRC) has the second highest incidence among cancers (1, 2), with 2.17 million patients worldwide in 2019, and is the major burden of cancer worldwide. Many studies have concluded that the incidence and death rate in CRC is closely related to the level of regional economy and medical care. CRC can be one of the highly controllable tumors (3, 4).

CRC incidence is a useful and highly accessible indicator that reflects the burden of CRC, trends in CRC prevention and control policies, and improvements and advances in medical care (5). Strengthening the prevention and control of CRC risk factors, such as obesity, unhealthy diet and sedentary behavioral interventions, can change the risk over time in all age groups (6). The implementation of early CRC screening methods, such as fecal occult blood detection and colonoscopy screening, may lead to an initial increase in newly diagnosed CRC cases due to centralized screening, thereby potentially raising the incidence of CRC. However, over time, early adenoma stage screening and treatment can reduce the long-term incidence of CRC by identifying and treating a subset of patients who may develop tumors. Additionally, timely detection and treatment can significantly decrease mortality rates (7–11).

We decomposed the risk of CRC into age, period, and birth cohort effects to illustrate different temporal trends. The risk of developing CRC varies not only by biological age (age effect) but also by birth cohorts over time as new diagnoses and cancer screening initiatives are introduced (period effect). In the early stages of life, reducing exposure to risk factors and early screening of the disease, such as early intervention for polyps under gastrointestinal endoscopy, will have a long–term impact on the incidence of CRC (cohort effect). In the past three decades, the incidence of CRC has been on the rise to varying degrees around the world, and many countries have unfavorable periods and cohort effects. Therefore, the in-depth analysis of different ages, periods, and cohort effects in various countries and regions is essential for optimizing regional prevention and control policies. This holds great significance in controlling the burden of CRC.

Although there are many studies on the incidence and death burden of CRC, some countries, mainly developed countries, have conducted in–depth analyses of age, period, and cohort effects but have not systematically explored them on a global scale. For example, Italy assessed the effectiveness of the fecal immunochemical test (FIT) screening program on CRC incidence via an age–period–cohort (APC) model (12). China, Singapore and Australia have also applied the APC model to reveal the incidence trend of CRC (13–16). However, in low–income countries, there is a lack of in–depth analysis of the incidence of CRC, especially the correlation between age, period and birth cohort. To the best of our knowledge, our study is the first to analyze the impact of age and period cohorts on CRC incidence in 204 countries and regions around the world from 1990 to 2019, disaggregated by age, sex, and comprehensively in depth.

## Methods

### Data sources

GBD 2019 provides an updated estimate of descriptive epidemiological data for 369 diseases and injuries in 204 countries and territories between 1990 and 2019. Using standardized tools within the Bayesian framework, the GBD network contributes all available data to generating disease estimates across time, age and geography, as well as across health causes and domains, allowing for “borrowing” information from available data to provide estimates for countries that do not have primary data sources. All GBD estimates in this article are provided with 95% uncertainty intervals (UIs) (17). Some prior distributions used in DisMod-MR, the Bayesian meta-regression tool used to simultaneously estimate incidence, prevalence, remission, excess mortality, and cause-specific mortality, were revised based on the simulation studies, showing that priors with less information help improve coverage of the UIs. To enhance the stability of models in the presence of the addition of subnational data in different GBD cycles, GBD2019 adopted a set of standard locations for the estimation of covariate effects in models (18).

Incidence data of CRC from 1990 to 2019 were from GBD tools.^1^

### Socio-demographic index

The SDI values of all countries are available on the webpage.^2^ SDI ranges from 0 to 1. It is an indicator to measure the overall fertility, education level and the lagging distribution of *per capita* income in a country. According to the SDI value in 2019, countries and regions are divided into five categories, low SDI (<0·45), low-middle SDI (≥0·45 and < 0·61), middle SDI (≥0·61 and < 0·69), high-middle SDI (≥0·69 and < 0·80), and high SDI (≥0·80).

### Overall time trend analysis of colorectal cancer incidence

Temporal trends in incidence rates during the study period were assessed by age–standardized incidence rates (ASIR), rates of change in incidence from 1990 to 2019, mean average annual percentage change (AAPC) and net drift. The AAPC was calculated based on the joinpoint regression model. Net drift was calculated based on the APC model.

At the same time, we divided the CRC incidence population into 4 age groups: 5–49 years old as the premature onset group, 50–69 years old as the middle–aged group, 70–84 years old as the old age group, and ≥ 85 years old as the superaged group. The trend of the incidence of each age group was plotted, and the incidence rate of each age group was calculated. Investigate the temporal distribution of age at onset as an indirect indicator of CRC burden.

### Joinpoint regression analysis

The Joinpoint regression model is a series of linear statistical models used to assess trends in CRC incidence over time. The model employs the least square method to estimate the pattern of incidence, thus avoiding the inherent subjectivity in typical trend analysis based on linear trends. The inflection point of the trend is determined by calculating the sum of squares of residuals between estimated and actual values. Natural logarithm regression is utilized to fit the incidence rates across different time periods, with annual percentage change (APC) and 95% confidence interval (CI) calculated for each period. Annual percentage change (AAPC) is employed to describe overall trends. The National Cancer Institute Joinpoint regression program (version 4.1.0) was used to construct this model, utilizing grid search algorithm and Bayesian information standard test to identify five connection points, with an overall alpha level set at 0.05.

### Age–period–cohort model analysis of incidence data

This study employed an Age-Period-Cohort (APC) model framework to investigate potential trends in CRC incidence by age, period, and birth cohort. APC models are widely utilized in epidemiological analysis of chronic diseases, including malignancies (19–21). In this study, the APC model was used to analyze the underlying trends of CRC incidence by age, period and birth cohort, and to uncover the impact of age-related biological factors as well as technical and social factors on CRC incidence trends.

The APC model implemented using the R tool fits a log-linear Poisson model on a Lexis plot of observed ratios and quantifies the additive effects of age, period, and birth cohort (22). Equally spaced age and time cycle intervals were used in the APC model of this study; i.e., five-year age groups were used in conjunction with five-year calendar cycles. GBD estimates are generated in data formats with unequal intervals (five-year age groups for each year of data), such as [1992] 1990–1994, [1997] 1995–1999, [2017] 2015–2019 representing specific time periods. There are 19 age groups at 5 year intervals from 5 to 9 years old to ≥95 years old representing specific ages. A birth cohort every 5 years based on the middle of the birth year from 1921 to 1929 (the 1925 cohort) to 2011–2019 (the 2015 cohort), totaling up to 24 cohorts. The fitted APC model estimates the overall time trend in incidence, expressed as the annual percentage change in incidence (net drift in incidence, % per year) (23).

The APC model also estimates time trends in incidence across age groups, expressed as a percentage change in incidence from year to year (local drift in incidence, % per year), reflecting trends in birth cohort effects. A drift of ±1% or more per year is considered a substantial change in incidence (19). The significance of annual percentage trends was tested using Wald’s chi–square test (19).

The APC model is capable of fitting age-, period-, and cohort-related risks (effects). The age effect represents the longitudinal age-specific rate fitted across the cohort to depict the age-related natural history. Period effects indicate the relative risk of death for each period, while cohort effects denote the relative risk of death for each cohort (24, 25). Statistical tests were two-sided, and significance was defined as *p* < 0.05. All analyses were conducted using R (version 4.1.2).

## Results

### Global and regional CRC incidence trends from 1990 to 2019

As can be seen in Supplementary Figure S1, in 2019, the ASIR of CRC was 26.71 (95% UI 24.58–28.89) per 100,000 people worldwide, second only to lung cancer (ASIR = 27.66, 95% UI 25.28–29.99). The incidence percent of CRC in the whole population is 13.22%, second only to lung cancer 13.79%. The incidence percent of CRC increases with age. Among people aged≥80 years, it ranks first, surpassing lung cancer. Therefore, in view of the increasing burden of CRC, our study will deeply analyze the temporal trend of colorectal cancer incidence and the age-period-cohort effect.

As shown in Table 1, in 2019, the number of new cases of CRC worldwide was 2.17 (95% UI 2.00–2.34) millions, an increase of 157.23% (95% UI 139.48–177.22%) compared with 1990. The ASIR of CRC in 2019 was 26.71 (95% UI 24.58–28.89) per 100,000 people, an increase of 20.06% (95% UI 12.08–29.19%) compared with 1990. Globally, the AAPC for CRC incidence in the past 30 years was 0.54% (95% CI 0.45–0.62%), and the APC model estimated a net drift in CRC incidence of 0.75% (95% CI 0.67–0.83%, *p* < 0.001) per year.

**Table 1:** Trends in the incidence of colorectal cancer across sociodemographic index quintiles and GBD regions, 1990–2019.

	Incidence			ASIR			Trend	
Location	1990 (95% UI)	2019 (95% UI)	Changes of number (%)	1990 (95% UI)^a^	2019 (95% UI)^a^	Percent of change (%)	AAPC (95% CI)^b^ (%)	Net drift (95% CI)^b^ (%)
Global	842,098 (810,408, 868,574)	2,166,168 (1,996,298, 2,342,842)	157.23 (139.48, 177.22)	22.25 (21.29, 22.97)	26.71 (24.58, 28.89)	20.06 (12.08, 29.19)	0.54 (0.45, 0.62)	0.75 (0.67, 0.83)
Sociodemographic index								
High SDI	443,895 (427,714, 453,338)	798,580 (715,631, 873,249)	79.9 (65.51, 95.02)	42.45 (40.91, 43.35)	42.78 (38.75, 46.64)	0.76 (−7.18, 9.38)	−0.06 (−0.16, 0.04)	0.1 (−0.04, 0.25)
High–middle SDI	237,898 (229,789, 246,091)	656,792 (595,972, 717,873)	175.67 (151.68, 202.01)	22.6 (21.75, 23.36)	32.45 (29.45, 35.44)	43.4 (30.81, 56.91)	1.14 (0.93, 1.34)	1.4 (1.29, 1.51)
Middle SDI	104,834 (97,278, 113,063)	517,606 (463,510, 578,243)	344.49 (291.88, 400.94)	10.23 (9.5, 11.03)	20.94 (18.77, 23.32)	84.53 (63.17, 107.7)	2.44 (2.3, 2.58)	2.33 (2.2, 2.46)
Low–middle SDI	40,778 (36,760, 45,652)	153,080 (138,796, 168,226)	272.63 (214.02, 326.62)	6.88 (6.23, 7.71)	11.35 (10.3, 12.44)	63.87 (38.15, 87.48)	1.79 (1.72, 1.86)	1.4 (1.34, 1.47)
Low SDI	14,278 (11,962, 16,828)	39,045 (34,913, 43,391)	158.78 (111.09, 213.23)	6.2 (5.19, 7.26)	7.74 (6.93, 8.59)	18.3 (−2.72, 41.67)	0.76 (0.7, 0.82)	0.61 (0.51, 0.71)
GBD regions								
High–income Asia Pacific	77,180 (73,961, 79,223)	196,371 (166,417, 225,643)	154.43 (120.07, 189.25)	38.69 (36.92, 39.76)	44.58 (38.38, 51.09)	15.22 (0.41, 31.09)	0.44 (0.18, 0.7)	0.22 (−0.04, 0.47)
High–income North America	167,902 (160,795, 172,260)	260,911 (229,909, 295,693)	55.39 (36.69, 76.03)	47.46 (45.59, 48.63)	42.71 (37.61, 48.61)	−10 (−21.05, 2.46)	−0.48 (−0.67, −0.29)	−0.05 (−0.23, 0.14)
Western Europe	229,473 (220,382, 234,886)	382,442 (332,800, 432,448)	66.66 (47.56, 87.92)	39.57 (38.03, 40.47)	42.42 (37.09, 48.26)	7.21 (−5.6, 21.6)	0.03 (−0.06, 0.13)	0.05 (−0.17, 0.27)
Australasia	12,029 (11,521, 12,422)	23,671 (19,439, 28,848)	96.78 (62.97, 138.6)	51.57 (49.33, 53.25)	48.35 (39.6, 59.06)	−6.25 (−22.72, 14.66)	−0.4 (−0.53, −0.28)	−0.07 (−0.33, 0.19)
Andean Latin America	2021 (1796, 2,243)	11,094 (8,935, 13,467)	448.83 (335.97, 586.38)	9.98 (8.85, 11.07)	19.96 (16.07, 24.18)	99.98 (59.63, 148.68)	2.56 (2.25, 2.86)	2.64 (2.42, 2.87)
Tropical Latin America	10,717 (10,338, 11,048)	42,891 (40,118, 44,928)	300.22 (277.79, 319)	12 (11.46, 12.39)	17.76 (16.58, 18.63)	48.05 (40.23, 54.83)	1.44 (1.3, 1.57)	1.42 (1.29, 1.54)
Central Latin America	7,477 (7,204, 7,656)	37,542 (32,211, 43,870)	402.12 (332.92, 482.98)	9 (8.59, 9.26)	15.93 (13.67, 18.62)	76.98 (52.76, 105.31)	1.99 (1.88, 2.11)	2.05 (1.93, 2.16)
Southern Latin America	10,929 (10,519, 11,262)	26,866 (21,480, 33,612)	145.82 (96.6, 204.81)	24.06 (23.07, 24.82)	32.22 (25.69, 40.39)	33.88 (6.76, 66.2)	0.9 (0.66, 1.13)	1.21 (1.02, 1.39)
Caribbean	4,690 (4,486, 4,854)	13,813 (11,813, 15,959)	194.49 (153.95, 237.68)	18.19 (17.34, 18.83)	26.73 (22.86, 30.86)	46.91 (26.53, 68.61)	1.32 (1.17, 1.48)	1.2 (0.96, 1.43)
Central Europe	41,586 (40,372, 42,590)	84,474 (74,551, 95,453)	103.13 (80.08, 128.66)	28.4 (27.5, 29.11)	39.87 (35.19, 45.07)	40.39 (24.39, 58.4)	1.18 (1.03, 1.32)	0.92 (0.77, 1.06)
Eastern Europe	70,401 (68,287, 72,718)	106,017 (96,250, 117,074)	50.59 (37.39, 66.56)	25.09 (24.32, 25.92)	31.11 (28.22, 34.37)	24.01 (12.97, 37.22)	0.9 (0.13, 1.68)	0.5 (0.36, 0.65)
Central Asia	6,746 (6,520, 6,979)	10,949 (9,999, 12,008)	62.32 (48.97, 78.91)	13.99 (13.51, 14.46)	15.21 (13.93, 16.61)	8.78 (−0.27, 19.1)	0.47 (0.3, 0.64)	−0.14 (−0.32, 0.04)
North Africa and Middle East	15,426 (12,968, 18,178)	60,010 (53,354, 67,555)	289.01 (209.76, 395.99)	9.02 (7.56, 10.56)	13.93 (12.32, 15.6)	54.47 (24.44, 97.98)	1.53 (1.42, 1.63)	1.4 (1.32, 1.49)
South Asia	29,941 (26,436, 34,063)	113,711 (98,190, 129,352)	279.78 (201.23, 353.39)	5.45 (4.79, 6.21)	8.31 (7.21, 9.43)	52.64 (20.56, 81.59)	1.6 (1.38, 1.81)	1.11 (1, 1.22)
Southeast Asia	27,898 (24,572, 30,662)	117,010 (96,631, 136,244)	319.41 (249.37, 385.19)	10.81 (9.6, 11.81)	19.3 (15.97, 22.4)	78.54 (47.77, 106.88)	2.01 (1.96, 2.06)	1.66 (1.59, 1.73)
East Asia	112,326 (100,313, 125,624)	637,096 (548,895, 738,549)	467.19 (367.77, 585.92)	12.77 (11.44, 14.26)	30.94 (26.75, 35.72)	142.27 (101.05, 190.89)	3.01 (2.76, 3.26)	3.27 (3.04, 3.49)
Oceania	246 (194, 295)	691 (555, 855)	181.05 (125.41, 247.26)	8.34 (6.63, 9.95)	9.99 (8.16, 12.13)	19.71 (−1.96, 45.48)	0.62 (0.56, 0.68)	0.5 (−0.19, 1.19)
Western Sub–Saharan Africa	5,434 (4,402, 6,640)	15,321 (12,895, 17,824)	181.95 (127.68, 251.36)	6.55 (5.35, 7.95)	8.72 (7.45, 10.03)	33.19 (9.2, 64.72)	0.98 (0.93, 1.04)	1.04 (0.89, 1.2)
Eastern Sub–Saharan Africa	5,196 (4,336, 6,144)	14,227 (12,130, 16,886)	173.81 (118.76, 241.68)	7.01 (5.83, 8.25)	8.83 (7.64, 10.37)	25.9 (2.34, 53.81)	0.82 (0.74, 0.91)	0.65 (0.49, 0.81)
Central Sub–Saharan Africa	1,612 (1,255, 2045)	3,957 (3,015, 5,113)	145.39 (67.6, 245.43)	7.42 (5.91, 9.27)	7.68 (5.92, 10.07)	3.5 (−27.81, 40.31)	0.08 (−0.02, 0.18)	0 (−0.3, 0.31)
Southern Sub–Saharan Africa	2,868 (2,504, 3,344)	7,106 (6,389, 7,882)	147.75 (121.35, 184.04)	10.73 (9.26, 12.71)	13.07 (11.8, 14.45)	21.76 (8.11, 41.11)	1.06 (0.78, 1.33)	0.3 (0.06, 0.53)

ASIR, age–standardized incidence; AAPC, average annual percentage change, which is calculated by joinpoint software, which is described the trend of colorectal cancer incidence. Net drifts are estimates derived from the age–period–cohort model and denotes overall annual percentage change in incidence, which captures the contribution of the effects from calendar time and successive birth cohorts. SDI, sociodemographic index.

a 95% UI: 95% uncertainty intervals.

b 95% CI: 95% confidence intervals.

In 5 SDI regions, the same as the global trend, there was an upward trend of varying degrees. Among them, the middle SDI region had the highest incidence rate. In the past 30 years, the incidence population in the middle SDI region shows dramatic growth by 344.49% (95% UI 291.88–400.94%), and the incidence rate has increased by 84.53% (95% UI 63.17–107.7%). The AAPC was 2.44% (95% CI 2.3–2.58%), and the net drift was 2.33% (95% CI 2.2–2.46%, *p* < 0.001). In the high SDI region, the incidence population increased by 79.9% (95% UI 65.51–95.02%) in the past 30 years, and the incidence rate showed a stable and declining trend, with an increase of 0.76% (95% UI −7.18–9.38%), the AAPC was −0.06% (95% CI −0.16–0.04%), and the net drift was 0.1% (95% CI −0.04–0.25%, *p* = 0.144). In low SDI region, the growth trend was relatively flat, with an AAPC of 0.76% (95% CI 0.7–0.82%) and net drift of 0.61% (95% CI 0.51–0.71%, *p* < 0.001).

In the 21 GBD regions, high–income North America had a higher economic index; the AAPC was −0.48% (95% CI −0.67%–−0.29%), and the net drift was −0.05% (95% CI −0.23–0.14%, *p* = 0.617). In Australasia, the AAPC was −0.4% (95% CI −0.53%–−0.28%), and the net drift was −0.07% (95% CI −0.33–0.19%, *p* = 0.589), showing a decreasing trend of incidence. East Asia, Andean Latin America, Central Latin America and other regions have shown a very obvious upward trend in the past 30 years, represented by East Asia; the AAPC was 3.01% (95% CI 2.76–3.26%), and the net drift was 3.27% (95% CI 3.04–3.49%, *p* < 0.001). High–income Asia Pacific and Western Europe showed a very stable trend. It is worth noting that in Central Asia, the AAPC was 0.47% (95% CI 0.3–0.64%), and the net drift was −0.14% (95% CI −0.32–0.04%, *p* = 0.116). In southern sub–Saharan Africa, the AAPC was 1.06% (95% CI 0.78–1.33%), and the net drift was 0.3% (95% CI 0.06–0.53%, *p* = 0.015). The trend displayed by net drift was far from that of AAPC.

**Figure 1:**
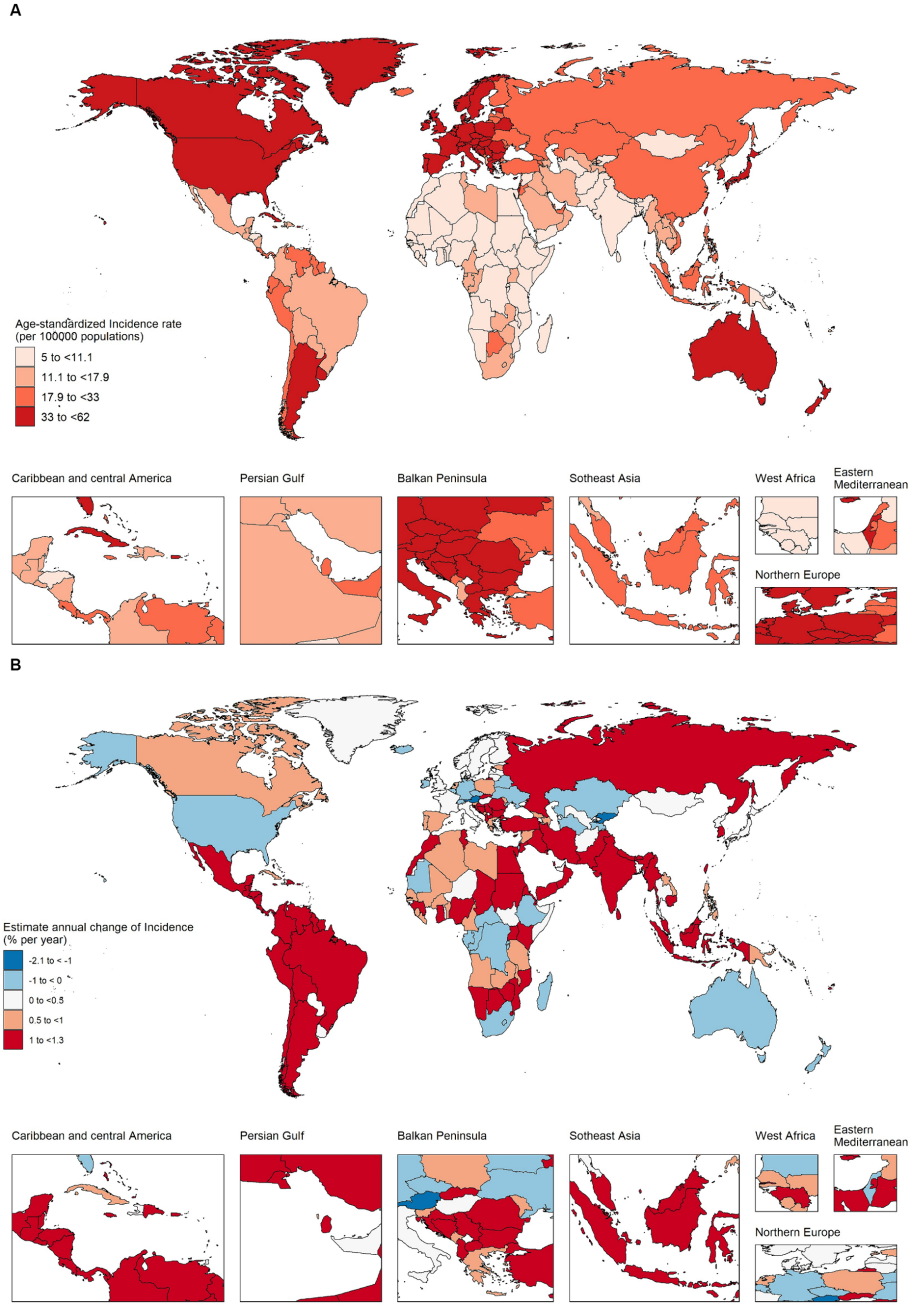
The age–standardized incidence in 2019 and net drift of incidence during 1990–2019 for colorectal cancer in 204 countries and territories. **(A)** World map of age–standardized incidence for colorectal cancer in 2019, **(B)** World map of net drifts for colorectal cancer incidence, i.e., estimated annual percentage change of mortality from age–period–cohort model. Net drift captures components of the trends attributable to calendar time and successive birth cohorts.

As shown in Figures 1A,B and Supplementary Table S1, in 2019, among 204 countries, the top three countries and regions with the highest ASIR of CRC were Taiwan Province of China (ASIR = 62.05, 95% UI 48.91–80.05), Monaco (ASIR = 60.69, 95% UI 48.55–73.57), Andorra (ASIR = 56.65 95% UI 42.79–71.9), and 30 countries had ASIR greater than 40 per 100,000 people, mainly concentrated in high and high–middle SDI regions. The top three countries and regions with the lowest ASIR were Malawi (ASIR = 6.27, 95% UI 4.87–7.8), Central African Republic (ASIR = 6.31, 95% UI 4.65–8.68), Democratic Republic of the Congo (ASIR = 6.36, 95% UI 4.24–9.58). And more than 40 countries have ASIR below 10 per 100,000 people, mostly concentrated in middle–low and low SDI regions. There were 31 countries with a downward trend (net drift <0), mainly concentrated in high SDI areas, represented by Austria, Kyrgyzstan, Czechia, etc. (net drift < ≤ − 0.5%). Countries with a more obvious upward trend were mainly concentrated in the middle SDI regions, represented by Vietnam, Equatorial Guinea, Saudi Arabia, China, etc. (net drift ≥3%). In low SDI regions, the ASIR was low, while maintaining a relatively stable growth trend (net drift < 1%). Except for a few regions, such as Mozambique, incidence rate increased by 78.34% (95% CI 31.22–136.33%), AAPC was 2.07% (95% CI 1.84–2.3%), net drift was 2.63% (95% CI 1.88–3.39%, *p* < 0.001), same trend in Uganda, Pakistan, Eritrea, Nepal, etc. High SDI region maintained high ASIRs, but in recent years, the increase in incidence has stabilized, and negative growth has occurred in some regions. However, in individual regions such as Taiwan (Province of China), the incidence rate increased by 149.2% (95% UI 95.4–219.77%), AAPC was 3.15% (95% CI 2.25–4.07%), and net drift was 2.86% (95% CI 2.59–3.14%, *p* < 0.001). Cyprus, Republic of Korea, Slovakia and other countries had the same trend. In most high–middle and middle SDI regions, the ASIR of CRC also maintained a high value and an obvious upward trend, such as China, in the past 30 years, the incidence population has dramatically increased by 473.97% (95% UI 369.16–600.91%), the incidence rate has increased by 144.07% (95% UI 99.97–195.49%), the AAPC was 2.99% (95% CI 2.71–3.26%), and the net drift was 3.31% (95% CI 3.08–3.55%, *p* < 0.001). There were also very few countries, such as Ukraine (AAPC 0.19% (95% CI −0.45–0.83%), net drift = −0.69% (95% CI −1.07%–−0.31%, *p* < 0.001)), United States of America (AAPC −0.56% (95% CI −0.77%–−0.35%), net drift = −0.12% (95% CI −0.3–0.07%, *p* = 0.219)), which had a downward trend. Collectively, these results suggested that trends in CRC incidence rates have been very uneven across countries and that increases in incidence rates have not necessarily matched expectations for SDI values. In addition, neither the rate of change in incidence nor the trend shown by AAPC calculated by joinpoint regression model was necessarily consistent with the net drift derived from the APC model, which indicated that it was necessary to distinguish the cycle and cohort effects of CRC incidence.

### Trends in the incidence of CRC in different age groups

**Figure 2:**
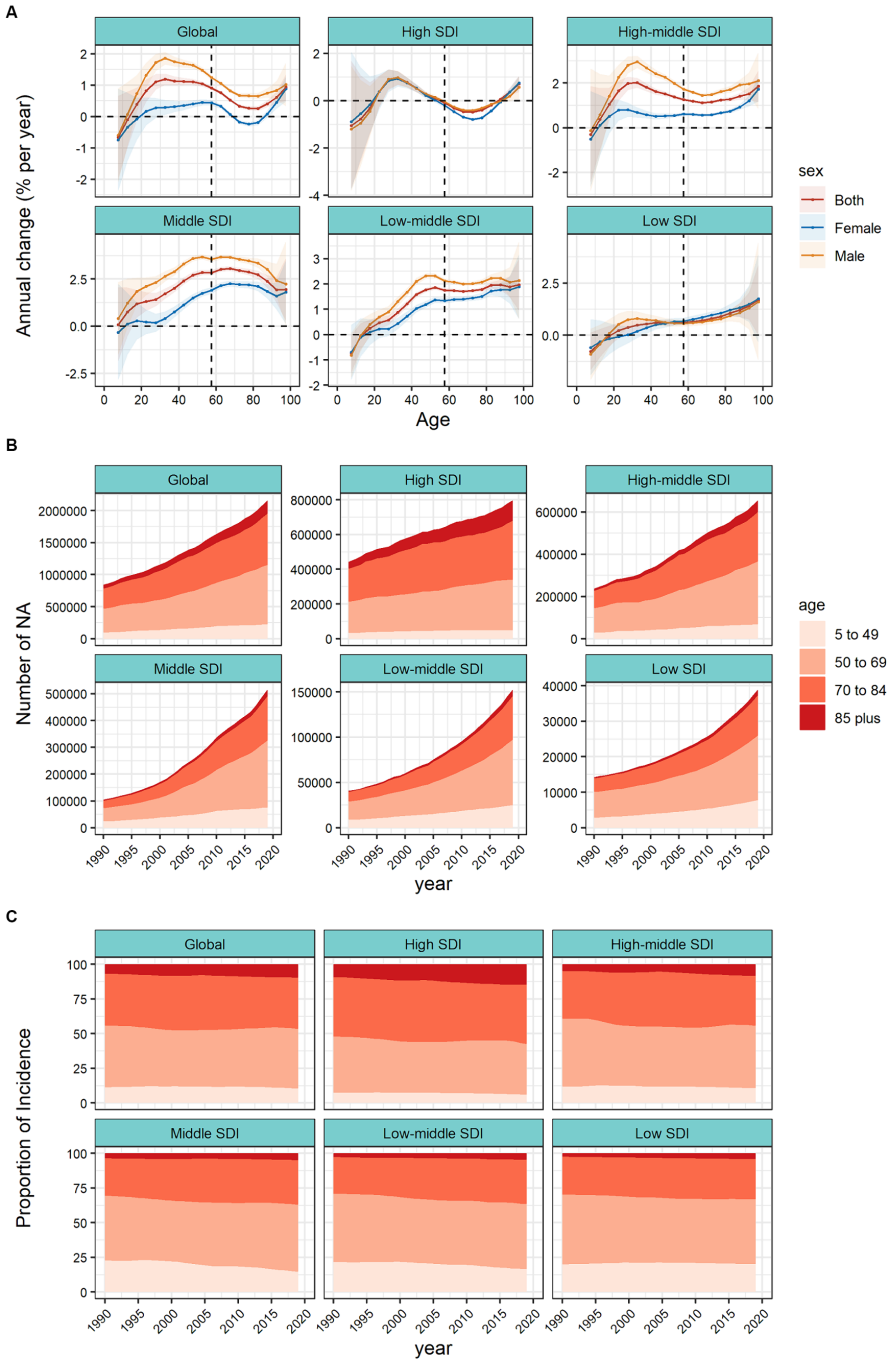
Local drifts of colorectal cancer incidence and age distribution of colorectal cancer incidence by SDI quintiles, 1990–2019. **(A)** Local drifts of colorectal cancer incidence (estimates from age–period–cohort models) for 19 age groups (5–9 to 95 plus years), 1990–2019. The dots and shaded areas indicate the annual percentage change of incidence (% per year) and the corresponding 95% CIs. **(B)** Temporal change in the absolute cases of colorectal cancer incidence across four age groups (5–49 years old as the premature onset group, 50–69 years old as the middle–aged group, 70–84 years old as the old age group, and ≥ 85 years old as the superaged group), 1990–2019. **(C)** Temporal change in the relative proportion of colorectal cancer incidence across four age groups (5–49 years old as the premature onset group, 50–69 years old as the middle–aged group, 70–84 years old as the old age group, and ≥ 85 years old as the superaged group), 1990–2019. SDI, sociodemographic index.

Figure 2A and Supplementary Table S2 shows the trend of annual percentage change in incidence for different age groups at 5–year intervals from age 5 to 95 plus years. Taking the world and the 5 SDI regions as an example, in addition to the 5–9 and 10–14 age groups, the incidence of CRC in all age groups has increased to varying degrees. The largest increase in incidence was in the 30–34 age group (1.19% per year (95% CI 1.01–1.37%)), followed by a weakening trend with age and a stronger trend after the 80–84 and older age groups. It should be noted that the increasing trend of the incidence of CRC in females at all ages was significantly lower than that in males. Among females, the incidence of CRC in the 70–74, 75–79, and 80–85 age groups showed a slight downward trend. In high SDI regions, the incidence of CRC was in the age group of 20–24 to 50–54 years old (the 30–34 age group had the highest growth trend, 0.96% (95% CI 0.67–1.26%)), and the ≥85 age group had the highest growth rate of 0.96% (95% CI 0.67–1.26%). This was consistent with the increasing trend of premature onset (≤50 years) reported in developed regions in many studies over the years. In the high–middle SDI, middle SDI and low–middle SDI regions, there was a clear upward trend in all age groups at the age of 20 and above. Among them, the peak growth rate in the middle–high SDI region was the age group of 30–34 age group (2.02% (95% CI 1.80–2.24%) per year), the peak growth rate in the middle SDI region was in the age group of 60–64, 65–70 years old, local drift >3%, and the 50–54 age group in the middle–low SDI region had the largest growth rate (1.86% (95% CI 1.79–1.94%) per year). In low SDI regions, starting from the age of 20–24, the growth trend gradually increased with age, reaching a peak in the 95 plus age group (1.69% (95% CI −0.017–3.42%) per year). Due to factors such as incomplete information registration, the confidence interval spans a great deal. At the same time, we observed that in the high–middle, middle and low-middle SDI regions, the sex difference was prominent in CRC incidence, and the growth trend in males was significantly higher than that in females. In the high and low SDI regions, the gender differences were not pronounced. As shown in Supplementary Figure S2A, among 21 GBD regions, high–income North America, Australasia showed a significant upward trend in the incidence of people aged 20–50 years which requires close attention. East Asia, Southeast Asia and most of Latin America saw a clear upward trend across all age groups. The rising trend of the incidence in Central Asia was concentrated in the age group of 60 years and above, and the rising trend in the High–income Asia Pacific was concentrated in the superaged group of 80 years and above. See Supplementary Figures S3–S7A in the attached table for the local drift of each country.

Figure 2B shows the changing trend of incidence population in the four age groups (5–49 years old as the premature onset group, 50–69 years old as the middle–aged group, 70–84 years old as the old age group, and ≥ 85 years old as the superaged group). Globally, the incidence population in each age group doubled in 30 years. In terms of case numbers, the burden of CRC has gradually shifted to the middle–aged group (50–69 years old). In high SDI regions, the core incidence group was mainly concentrated in old age group (70–84 years old), while superaged group (≥ 85 years old) increased significantly. Represented by the middle SDI region, the incidence population was concentrated in the middle–aged group (50–69 years old), and the increase was nearly 5 times. In Figure 2C, we further analyzed the temporal changes in the distribution of the four age groups. Globally, middle–aged group (50–69 years old) are still the core population of CRC incidence, the proportion of premature–onset group (5–49 years old) has stabilized, and the proportion of superaged group (≥ 85 years old) has been increasing year by year. In different SDI regions, we found that the proportion of premature onset group (5–49 years old) in middle, middle–low, and low SDI regions were significantly higher than those in high and high–middle SDI regions. Meantime the premature onset proportion in the low SDI region was still on the rise. In high and high–middle SDI regions, the proportion of superaged group (≥ 85 years old) showed an upward trend, and were significantly higher than other regions. In the 21 GBD regions, high–income Asia Pacific, Australasia, Western Europe and other high SDI regions, the proportion of premature onset group (5–49 years old) were low, but the proportion of superaged group (≥ 85 years old) were increasing year by year. In Oceania, most regions of Africa, the proportion of premature onset group was noteworthy (Supplementary Figures S2B,C). The trend of age proportion in each country is shown in the attached Supplementary Figures S3–S7B,C.

### APC model (influence of age, period, cohort on CRC incidence)

Taking the 5 SDIs as an example, Supplementary Figure S8 plots the interaction among age, period, and birth cohort. Supplementary Figure S8A plots the incidence rate of each age group in different periods. In the high–middle SDI region and middle SDI region, the incidence rate of people over 60 years old increased significantly under different periods. Supplementary Figure S8B plots the incidence of different birth cohorts for each age group, taking the middle SDI as an example, the incidence of the same age group gradually increased with the birth cohort time until the 1970 birth cohort. Supplementary Figure S8C plots the changes in the incidence of different birth cohorts in different periods. In the same period, represented by the high SDI region, the incidence of different birth cohorts changed significantly. Age, period, and cohort factors all affect the incidence of CRC.

**Figure 3:**
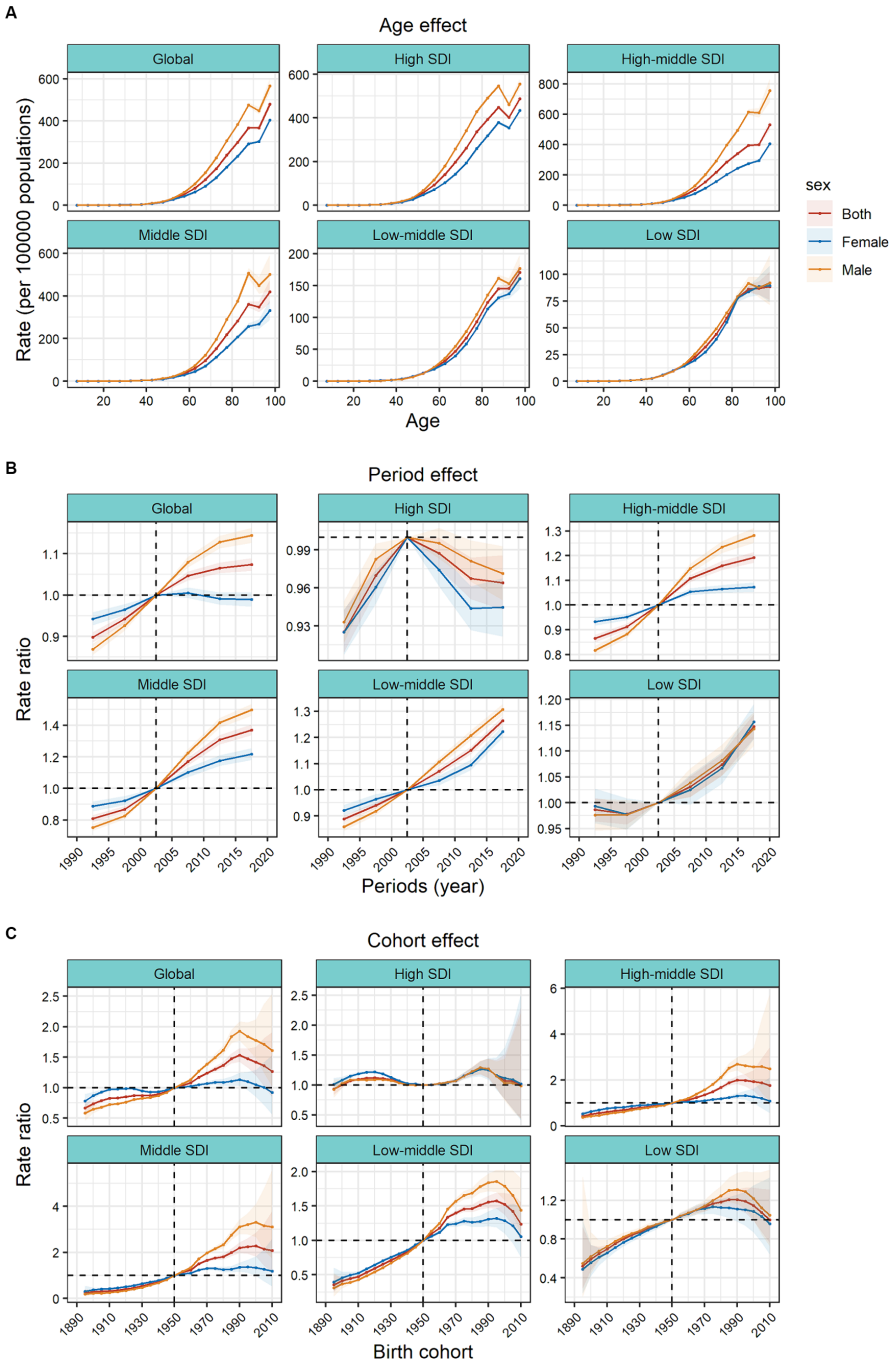
Age, period and cohort effects on colorectal cancer incidence by SDI quintiles. **(A)** Age effects are shown by the fitted longitudinal age curves of incidence (per 100,000 people–years) adjusted for period deviations. **(B)** Period effects are shown by the relative risk of incidence (incidence rate ratio) and computed as the ratio of age–specific rates from 1990–1994 to 2015–2019 (2000–2005 as the reference period). **(C)** Cohort effects are shown by the relative risk of incidence and computed as the ratio of age–specific rates from the 1895 cohort to the 2010 cohort, with the reference cohort set at 1950. The dots and shaded areas denote incidence rates or rate ratios and their corresponding 95% CIs. SDI, sociodemographic index.

The APC model further analyzed the age–period–cohort effects of the 5 SDIs and different regions (i.e., the age effect, expressed as a longitudinal age curve, to represent the natural history of age–related CRC incidence; the cycle effect, expressed as relative incidence risk by period, with for tracking progression over time; cohort effects, expressed as relative risk of incidence by cohort, for tracking changes in incidence across birth cohorts). Consistent age effects were found across the global and 5 SDI regions, with increasing incidence rates with increasing age, noting that there were significant gender differences in high, high–middle, and middle SDI regions (Figure 3A).

Period effects generally showed an increasing risk of CRC incidence across different SDI quintiles over the study period. At the same time, the period effects had increased significantly over the past 15 years in areas except high SDI, which indicated the uncontrolled increase in incidence. But for high SDI, the period effect has shown a downward trend over the past 15 years, indicating initially incidence improvement. The period effect showed significant gender differences. In the high SDI region, period risk in male was higher than that in female during past 30 years. However, in high-middle, middle, low-middle SDI regions, the risks in males had increased significantly over the past 15 years and surpassed that in females. During this period, there were no significant gender differences in low SDI region (Figure 3B).

Birth cohort risk was relatively stable in the high SDI region, with two flat growth peaks during 1910–1930 and 1980–1990 birth cohorts, with no significant sex differences. In other SDI regions, the cohort effects maintained a certain uniformity, increasing cohort risks were more noticeable in those born after the 1920s. Sex differences existed in the high–middle, middle, and low–middle SDI regions, and in the post-1950 birth cohort, the cohort risks had significant sex differences. The risks in males were significantly higher than that in females (Figure 3C).

In the 21 GBD regions, the age effects gradually increased with age. Among them, the sex differences of age effects were obvious in the high–income Asia Pacific, East Asia and Europe. In these regions, the age effects of males were significantly higher than those of females. In most regions, the period risks increased significantly in past 15 years, while the high–income North America, Western Europe, and Australasia regions showed a downward trend. There were large gender gaps of period risks in Australasia and East Asia. Represented by East Asia, Central Latin America, and Andean Latin America, the increasing cohort effects were notably after the 1990 cohort. However, it showed a downward trend in cohort risk after the 1990 cohort in Central Asia. In East Asia, the cohort effects showed significant gender differences in those born after the 1990s (Supplementary Figures S9, S10).

### APC effects in model countries

**Figure 4:**
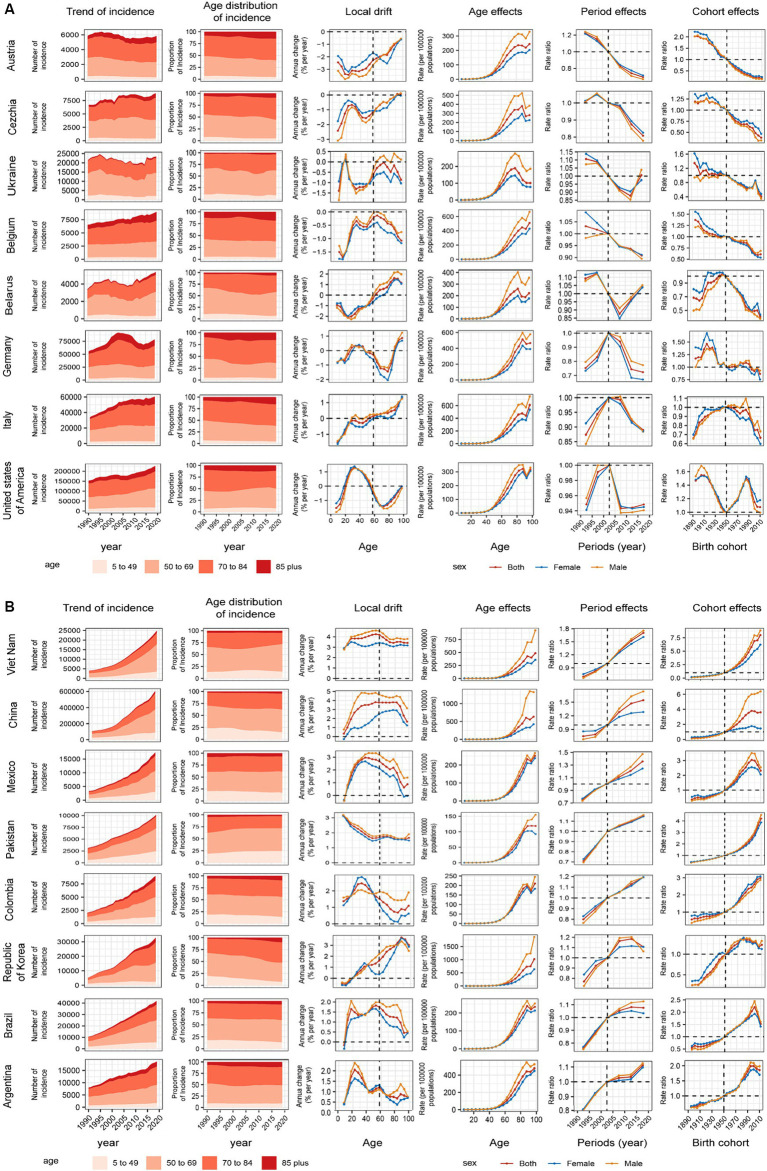
Positive **(A)** and adverse **(B)** age–period–cohort effects on model countries across SDI quintiles. Temporal change in the absolute cases of colorectal cancer incidence across four age groups, 1990–2019. The age distribution of deaths shows the relative proportion of incidence from each age group during 1990–2019. Local drifts of colorectal cancer incidence (estimates from age–period–cohort models) for 19 age groups (5–9 to 95 plus years), 1990–2019. Age effects are shown by the fitted longitudinal age curves of incidence (per 100,000 people–years) adjusted for period deviations. **(B)** Period effects are shown by the relative risk of incidence (incidence rate ratio) and computed as the ratio of age–specific rates from 1990–1994 to 2015–2019 (2000–2005 as the reference period). Cohort effects are shown by the relative risk of incidence and computed as the ratio of age–specific rates from the 1895 cohort to the 2010 cohort, with the reference cohort set at 1950. The shaded areas indicate the corresponding 95% CIs of each point estimate. SDI, sociodemographic index.

We introduced several representative countries to better describe the APC effect of global CRC incidence trends. Figure 4A shows countries with favorable age–cycle cohort effects, mostly concentrated in high and high–middle SDI regions. Represented by countries with high SDI, such as Australia, Czechia, and Belgium, the risk of age increased with natural age increase in the past 30 years, the risk of period and cohort gradually decreased, and the incidence of CRC was well controlled. Negative growth was 2.44% (95% UI −3.09%–−1.79%, *p* = 0.891) in Australia. At the same time, there was no obvious gender gaps in the period and cohort effects in these countries, and the gender difference was mainly reflected in the age effect of natural age increase. In the United States of America, the risk decreased significantly in the past 15 years, the cohort risks increased in the post-1950s birth cohort but decreased until the post-1980s birth cohort. Germany and Israel showed a significant downward trend in risk in the past 15 years, and the cohort risks were stable in the post-1950s birth cohort. After a period of declining risk in Ukraine and Belarus, the risk began to increase in the last decade, respectively, the birth cohort effects dropped significantly in the post-1950s birth cohort. In these countries, the incidence of CRC was significantly controlled due to time and/or cohort risk reduction.

Figure 4B shows countries with unfavorable age–period-cohort effects, mostly concentrated in middle SDI regions. Both period and cohort risks worsened to varying degrees in these countries, in addition to the naturally increasing age risk. Taking China as an example, the period risks increased significantly in past 15 years, the cohort effects increased gradually in the post-1950s birth cohort, and gender differences were evident in age, period, and cohort risk. This was also the reason for the large difference between males and females in the incidence trend in the high–middle SDI region. The Republic of Korea period risks have been initially controlled in recent years. In Argentina, Brazil, Mexico, Cohort risks had improved in the post-2000 birth cohort. The age–period–cohort risk for all countries is shown in Supplementary Figures S11–S20.

## Discussion

Colorectal cancer (CRC) is currently one of the cancers that has the heaviest burden. Therefore, controlling the incidence of CRC is the focus of tumor prevention and control (25). Consistent with previous research reports on CRC, the incidence of CRC in the world shows a clear upward trend. However, there is significant heterogeneity in the growth rate of different regions. In some countries with high SDI, the incidence of CRC has been flat or declining (14). East Asia and parts of Latin America have a significant increase in incidence (2, 26, 27). However, the control of disease burden is not completely consistent with the economic and social level. Countries such as Kuwait, Republic of Korea, and Slovakia have higher SDI values, but the incidence still shows a significant increase. At the same time, there are different gender burdens in different regions and different growth trends in different age groups. Data from many countries in low SDI regions are model–based, therefore, more accurate and further studies are needed to verify true incidence trends. Assessing change using overall rate or Joinpoint regression models ignores important information about differences in age, time period, and birth cohort, and our study found that the growth rate of incidence in many regions differed significantly from the net drift calculated by the APC model, which is very significant. It is necessary to distinguish the influence of the age–period–cohort effect on the incidence of colorectal cancer to accurately obtain the epidemic characteristics of different regions and formulate efficient prevention and control policies.

Our study shows that there is a beneficial age–period–cohort effect in some countries and regions, and the incidence of CRC appears to be flat or even decreasing. Most of these countries are concentrated in high SDI regions. Countries with high SDI, such as Austria, the United States, Italy, and Australia, have strengthened relevant health promotion measures in recent decades, such as implementing smoking bans, alcohol restriction policies, increasing fiber intake, encouraging exercise, and actively controlling metabolic–related diseases. The implementation of a series of public health policies has had a profound impact on the control of CRC incidence and mortality (5, 28). These countries have also implemented a series of early screening policies for CRC, such as FIT, CT colonography, colorectal screening and early–stage disease resection, which will help reduce CRC in the long run. At the same time, early diagnosis and early treatment can help reduce CRC mortality (29). The United States has been insured for CRC screening since 1998 and has a Specialized Multi–Social Task Force (MSTF) for CRC (7). Our study showed a decline in risk in the United States in the past 15 years and a gradual decline in risk in the post–1990 birth cohort. In Australia, Australia’s National Bowel Screening Program (NBCSP) was launched in 2006, implemented and gradually improved (30), and our study shows a significant reduction in risk in Australia in the last decade. Fecal occult blood testing (FOBT) was introduced in Belgium in 2009, and FIT screening was further expanded in 2016 (31). Our study showed that the risk of CRC incidence decreased in the period in past 15 years. This series of early screening policies, as well as a series of healthy life advocacy policies, such as smoking cessation and alcohol restriction, have brought the incidence of CRC under control.

In contrast, CRC incidence and death rate has increased in regions with low SDIs and in some transition countries, such as China, Mexico, and Vietnam, due to dramatic changes in lifestyle and dietary patterns, smoking, diet, and metabolism. Obviously (32, 33). At the same time, inadequate national screening prevention policies and inadequate healthcare improvements are contributing factors. Our study also showed very unfavorable age–period–cohort effects in these regions. In 2016, the Chinese government put forward the “Healthy China 2030” plan, which clearly pointed out reasonable diet, tobacco control, alcohol control, and the implementation of actions to improve the quality of medical services for the whole population (34). It is expected that effective prevention and control policies will gradually control the incidence in these areas. Despite high SDI values, South Korea has a clear upward trend in incidence with unfavorable age–period–cohort risk. In addition to dietary habits and lifestyle reasons, the lack of early attention to CRC screening policy is also one of the main reasons (35, 36).

Our study found that the 30–34 age group had the most pronounced increase in incidence worldwide, especially in areas with high and high–middle SDIs, which is consistent with the early onset phenomenon of CRC pointed out by many current studies around the world (37). The early onset of CRC is currently considered to be significantly correlated with the occurrence of a Western–style diet, obesity, and early onset of diabetes (38–41). At the same time, we also observed superaged (≥85 years) in high SDI regions, which is associated with deep population aging in developed countries. In the middle and middle–low SDI regions, the most significant increase is still in the middle–aged population. For these regions, prevention and control policies should pay more attention to the implementation of early screening policies for CRC, such as FIT and colonoscopy screening for people aged 45 and above.

The sex difference in the incidence of CRC is mainly reflected in high–middle–, middle– and middle–low–SDI regions. The incidence and growth trend of males were significantly higher than those of females, and the gender gap gradually increased. Asia and Latin America are the regions with the most significant gender differences, which may be related to the uncontrolled behaviors and lifestyles of men in these regions, such as smoking, drinking, and physical inactivity (5). However, in areas with high and low SDIs, the gender difference is very limitable. In high SDI regions, the implementation of the policy of smoking and restricting alcohol and tobacco is beneficial to reduce the gender difference (42, 43).

To our knowledge, our study is the first to use the APC model to comprehensively analyze time–period trends and age–period–cohort effects in different countries worldwide. Compared to previous studies, we have achieved a more comprehensive and precise understanding of the burden of CRC. In particular, examination of period and cohort effects allows us to differentiate incidence by time period and birth cohort in each country to inform the effectiveness of CRC–related health care services. Estimates of local drift values allowed us to capture temporal trends in incidence for each age group, adjusted for period effects. Of course, our study has certain limitations, and due to the complexity of the data, the interpretation of the trend of CRC incidence needs to be cautious. The accuracy of GBD data depends on the quality and quantity of data collected, and many economically underdeveloped regions, such as South America, Asia, and Africa, lack high–quality cancer registries. In some countries with no data, GBD estimates depend heavily on the choice of covariates in the model and the regional model.

## Conclusion

The growing burden of colorectal cancer requires urgent global attention. Our age–period–cohort analysis of colorectal cancer incidence found that colorectal cancer incidence trends were not always consistent with socioeconomic development despite some correlation. Current research shows that many countries are currently insufficient in the prevention, control and management of colorectal cancer burden. At the same time, the medical and economic levels of many countries cannot afford a sound colorectal cancer early screening policy. We should pay more attention to the early onset of colorectal cancer and formulate cost–effective prevention and control policies according to local conditions for different age groups and incidence trends.

## Data availability statement

The original contributions presented in the study are included in the article/Supplementary material, further inquiries can be directed to the corresponding authors.

## Author contributions

YZ: Writing – original draft. X-BZ: Writing – review & editing, Methodology, Resources, Software. Y-WD: Writing – original draft. YK: Writing – original draft, Formal analysis. X-FZ: Writing – original draft, Project administration. P-HL: Writing – original draft. YT: Writing – original draft, Funding acquisition. Q-WZ: Writing – review & editing.

## Footnotes

^1^ https://ghdx.healthdata.org/gbd-results-tool

^2^ http://ghdx.healthdata.org/record/ihme-data/gbd-2019-socio-demographic-index-sdi-1950-2019
