# Supplementary material for: Distinct time trends in colorectal cancer incidence in countries with SDI levels from 1990 to 2019: an age–period–cohort analysis for the Global Burden of Disease 2019 study
Source: Front Public Health. 2024 May 22;12:1370282. doi: 10.3389/fpubh.2024.1370282 (PMC11150799; doi:10.3389/fpubh.2024.1370282)
Supplement: Supplementary file 1 [file Data_Sheet_1.PDF]

Supplementary appendix to

Distinct time trends in colorectal cancer incidence in countries with SDI levels from 1990 to 2019: An age-period-cohort analysis for the Global Burden of Disease 2019 study

Table S1 The incidence cases, age-standardized incidence rate of CRC in 1990 and 2019, and their variations from 1990 to 2019.

	Incidence			ASIR			AAPC	Net drift
location	incidence in 1990 (95% UI)	incidence in 2019 (95% UI)	percent change 1990-2019,% (95% UI)	ASIR in 1990 (95% UI)	ASIR in 2019 (95% UI)	percent change 1990-2019,% (95% UI)	average annual percentage change of ASIR from Joinpoint Regression analysis (95% CI)	drift of mortality from APC model, % per year (95% CI)
High SDI								
Switzerland	3206 (3023, 3381)	5824 (4529, 7396)	81.63 (41.7, 129.79)	30.71 (29.09, 32.26)	33.59 (25.91, 42.71)	9.38 (-14.64, 39.43)	0.21 (0.09, 0.33)	-0.32 (-0.9, 0.26)
Norway	2791 (2645, 2905)	4746 (4026, 5535)	70.05 (45.96, 97.05)	40.85 (38.89, 42.41)	49.46 (41.9, 57.73)	21.09 (3.39, 40.63)	0.59 (0.35, 0.84)	0.44 (-0.24, 1.11)
Monaco	32 (25, 39)	57 (46, 68)	78.85 (35.62, 132.81)	45.64 (36.26, 55.36)	60.69 (48.55, 73.57)	32.99 (-1.55, 76.53)	1.07 (0.97, 1.16)	1.1 (-6.55, 9.37)
Germany	55726 (53110, 57808)	78951 (62925, 101417)	41.68 (11.63, 81.57)	43.58 (41.64, 45.17)	41.4 (32.67, 53.84)	-5.01 (-25.72, 23.6)	-0.19 (-0.9, 0.52)	-0.34 (-1.47, 0.81)
Luxembourg	245 (229, 262)	372 (302, 451)	51.8 (23.11, 83.77)	44.89 (41.96, 47.94)	37.17 (30.27, 45.07)	-17.2 (-33.05, 0.32)	-0.86 (-1.06, -0.66)	-0.92 (-3.08, 1.3)
Andorra	29 (22, 38)	79 (60, 101)	177.58 (91.79, 282.2)	53.3 (41.79, 70.73)	56.65 (42.79, 71.9)	6.29 (-26.48, 44.17)	0.2 (0.08, 0.31)	0.12 (-5.4, 5.97)
Denmark	3160 (3026, 3283)	5577 (4364, 7017)	76.5 (37.97, 120.68)	39 (37.42, 40.5)	48.41 (37.76, 61.15)	24.13 (-3.69, 56.2)	0.63 (0.29, 0.98)	0.32 (-0.37, 1.01)
San Marino	15 (13, 17)	32 (25, 42)	116.91 (60.81, 189.9)	44.35 (38.44, 50.48)	49.55 (37.89, 65.53)	11.72 (-18.45, 50.61)	0.43 (0.36, 0.5)	0.81 (-7.14, 9.44)

Netherlands	8766 (8319, 9149)	18774 (14703, 23598)	114.16 (68.59, 168.24)	43.93 (41.77, 45.81)	55.39 (43.29, 69.76)	26.1 (-1.39, 58.59)	0.66 (0.16, 1.17)	0.52 (0.14, 0.91)
United Arab Emirates	73 (48, 101)	762 (521, 1030)	946.51 (488.37, 1621.73)	19.74 (12.74, 26.5)	21.36 (14.7, 29.76)	8.2 (-36.04, 71.17)	0.2 (-0.11, 0.5)	0.5 (-1.18, 2.2)
Republic of Korea	5027 (4773, 5316)	32929 (27484, 39146)	555.1 (445.14, 681.24)	16.97 (16.02, 18.04)	37.16 (31.14, 44.01)	119.04 (83.62, 160.27)	2.5 (2.1, 2.91)	1.57 (1.15, 1.98)
Canada	14718 (13933, 15384)	33633 (26448, 42308)	128.52 (78.1, 191.04)	45.47 (43.09, 47.46)	49.6 (38.84, 62.84)	9.09 (-15.06, 39.21)	0.2 (0.04, 0.35)	0.56 (0.11, 1)
Sweden	5049 (4767, 5280)	7720 (6590, 8930)	52.9 (31.2, 76.9)	33.51 (31.71, 34.94)	36.99 (31.41, 42.78)	10.37 (-5.18, 27.62)	0.27 (0.04, 0.5)	0.35 (-0.17, 0.86)
Japan	71184 (68046, 73123)	160211 (130730, 186831)	125.07 (90.18, 160.94)	42.2 (40.23, 43.4)	47.59 (40.18, 55.59)	12.77 (-4.22, 31.52)	0.48 (0.33, 0.64)	0.21 (-0.1, 0.52)
Iceland	85 (78, 92)	169 (147, 195)	99.81 (70.66, 132.31)	29.62 (27.3, 32.18)	30.71 (26.82, 35.36)	3.68 (-11.52, 20.58)	0.08 (-0.09, 0.26)	-0.06 (-2.92, 2.89)
Taiwan (Province of China)	4085 (3940, 4230)	24210 (19139, 31206)	492.59 (364.79, 661.76)	24.9 (23.96, 25.77)	62.05 (48.91, 80.05)	149.2 (95.4, 219.77)	3.15 (2.25, 4.07)	2.86 (2.59, 3.14)
Ireland	1779 (1692, 1861)	3431 (2687, 4295)	92.87 (50.76, 142.71)	43.76 (41.69, 45.74)	45.95 (35.89, 57.72)	5.02 (-18.07, 32.68)	-0.01 (-0.19, 0.17)	-0.09 (-0.82, 0.66)
Singapore	931 (880, 981)	3093 (2471, 3847)	232.18 (163.7, 312.97)	42.95 (40.48, 45.21)	39.93 (31.93, 49.46)	-7.02 (-25.65, 14.96)	-0.24 (-0.4, -0.09)	-0.69 (-1.37, 0)
United States Virgin Islands	22 (18, 25)	79 (66, 92)	264.68 (190.56, 348.66)	26.44 (22.61, 30.13)	43.04 (35.69, 50.62)	62.8 (30.14, 100.4)	1.66 (1.33, 1.99)	1.22 (-2.92, 5.54)
Finland	1868 (1782, 1952)	3799 (2973, 4805)	103.42 (58.51, 159.8)	26.14 (24.95, 27.3)	31.46 (24.55, 40)	20.34 (-6.85, 54.53)	0.7 (0.64, 0.77)	0.46 (-0.3, 1.22)
Kuwait	64 (57, 70)	454 (377, 545)	610.07 (482.6, 764.06)	10.12 (8.97, 11.3)	17.78 (14.65, 21.32)	75.73 (46.31, 114.62)	1.68 (0.21, 3.18)	2.17 (0.9, 3.46)
Belgium	6564 (6198, 6876)	8994 (7126, 11223)	37.01 (8.12, 72.81)	42.43 (40.15, 44.39)	39.33 (30.86, 49.46)	-7.29 (-26.95, 16.65)	-0.37 (-0.45, -0.29)	-0.53 (-1.02, -0.05)
Austria	5881 (5619, 6151)	5847 (4783, 7109)	-0.58 (-18.6, 21.41)	50.14 (47.99, 52.36)	33.07 (26.71, 40.55)	-34.05 (-46.72, -19.07)	-1.53 (-1.79, -1.27)	-2.44 (-3.09, -1.79)
United Kingdom	38718 (37268, 39640)	54429 (45870, 63942)	40.58 (18.72, 65.45)	42.73 (41.17, 43.7)	43.6 (36.6, 51.5)	2.03 (-14.33, 20.59)	0.13 (-0.18, 0.43)	0.33 (0.09, 0.57)

Lithuania	1107 (1060, 1156)	1669 (1385, 2008)	50.77 (24.54, 80.95)	24.65 (23.6, 25.8)	29.2 (24.01, 35.37)	18.49 (-2.46, 42.94)	0.54 (-0.38, 1.46)	0.29 (-0.71, 1.31)
Cyprus	160 (141, 182)	739 (638, 843)	361.73 (275.67, 454.61)	20.45 (18.17, 23.34)	38.15 (33.1, 43.43)	86.54 (52.33, 122.36)	2.09 (1.84, 2.34)	2.36 (0.62, 4.12)
Slovenia	803 (621, 1024)	1726 (1347, 2222)	114.84 (48.28, 198.02)	32.94 (25.35, 41.98)	41.05 (31.88, 52.95)	24.64 (-13.94, 74)	0.45 (0.07, 0.83)	0.51 (-0.71, 1.75)
New Zealand	2389 (2267, 2512)	4326 (3591, 5137)	81.06 (49.42, 115.32)	61.45 (58.21, 64.59)	55.34 (45.72, 65.79)	-9.94 (-25.81, 6.96)	-0.61 (-0.71, -0.52)	-0.34 (-0.98, 0.31)
Australia	9640 (9198, 9992)	19345 (15224, 24451)	100.67 (58.66, 153.89)	49.59 (47.32, 51.49)	47.04 (36.95, 59.55)	-5.15 (-25.33, 20.46)	-0.39 (-0.53, -0.25)	-0.02 (-0.3, 0.26)
Estonia	634 (603, 664)	1118 (892, 1391)	76.32 (39.65, 120.94)	30.9 (29.45, 32.33)	42.21 (33.4, 52.75)	36.62 (7.14, 72.61)	0.98 (0.3, 1.67)	0.84 (-0.48, 2.17)
France	32302 (30506, 33816)	52277 (40682, 65962)	61.84 (28.54, 104.35)	38.68 (36.69, 40.42)	38.47 (30.1, 49.3)	-0.55 (-21.94, 25.98)	-0.1 (-0.21, 0.01)	0.05 (-0.25, 0.35)
Qatar	15 (12, 20)	191 (137, 258)	1140.58 (692.44, 1771.57)	15.58 (11.57, 19.67)	25.05 (19.33, 31.85)	60.79 (10.14, 134.95)	1.51 (0.89, 2.13)	1.81 (-0.71, 4.39)
Czechia	6723 (6472, 6957)	8839 (7288, 10730)	31.47 (6.97, 59.67)	48.79 (46.99, 50.49)	42.14 (34.45, 51.15)	-13.62 (-30.31, 5.19)	-0.8 (-1.15, -0.45)	-0.95 (-1.5, -0.41)
Brunei Darussalam	38 (32, 45)	138 (122, 159)	262.82 (181.03, 355.18)	40.74 (34.27, 47.57)	49.38 (43.44, 55.88)	21.18 (-3.24, 50.61)	0.67 (0.39, 0.94)	1.13 (-1.12, 3.44)
Latvia	905 (866, 945)	1214 (1015, 1461)	34.12 (11.95, 61.99)	25.33 (24.25, 26.45)	30.54 (25.44, 36.96)	20.58 (0.37, 47.16)	0.45 (-0.3, 1.2)	0.2 (-1.09, 1.5)
Puerto Rico	775 (730, 814)	2379 (1864, 3026)	207.18 (138.49, 289.52)	21.42 (20.15, 22.53)	34.99 (26.99, 44.78)	63.33 (25.67, 108.97)	1.75 (1.49, 2.01)	1.73 (0.76, 2.71)
Bermuda	25 (23, 27)	56 (47, 68)	122.68 (83.54, 173.09)	41.25 (37.92, 44.44)	43.29 (36.11, 52.78)	4.95 (-13.32, 28.31)	0.23 (0.16, 0.29)	0.47 (-4.95, 6.2)
Guam	16 (14, 18)	40 (33, 47)	148.58 (96.04, 212.4)	21.74 (18.06, 25.48)	21.01 (17.62, 24.81)	-3.39 (-23.58, 21.96)	-0.04 (-0.4, 0.33)	0.16 (-2.96, 3.38)
Slovakia	2282 (2171, 2402)	5203 (4087, 6574)	127.98 (80.65, 188.29)	38.16 (36.33, 40.06)	56.45 (44.36, 71.04)	47.91 (17.18, 87.11)	1.81 (1.43, 2.2)	1.08 (0.39, 1.77)
High middle SDI								
Saudi Arabia	388 (272, 528)	3167 (2418, 4019)	715.35 (405.25, 1173.06)	6.64 (4.6, 8.96)	15.36 (12.16, 18.83)	131.13 (46.67, 259.6)	2.99 (2.89, 3.09)	3.57 (3.08, 4.06)

Russian Federation	41739 (40276, 43259)	71542 (62884, 81644)	71.41 (51.03, 95.32)	23 (22.17, 23.86)	30.77 (27.02, 35.11)	33.8 (17.69, 52.4)	1.07 (0.85, 1.29)	1.01 (0.82, 1.2)
Israel	1538 (1453, 1619)	3893 (3050, 4900)	153.19 (100.18, 221.66)	31.87 (30.08, 33.55)	33.64 (26.3, 42.59)	5.55 (-16.82, 34.84)	-0.13 (-0.51, 0.26)	-0.38 (-0.89, 0.13)
Poland	10521 (10184, 10780)	24277 (20748, 28769)	130.74 (97.39, 171.85)	24.23 (23.38, 24.84)	34.96 (29.84, 41.55)	44.29 (23.35, 69.74)	1.25 (1.02, 1.48)	0.91 (0.6, 1.22)
Malta	120 (110, 130)	303 (256, 361)	153.76 (108.12, 205.1)	28.1 (25.83, 30.43)	32.92 (27.83, 39.12)	17.14 (-3.58, 39.99)	0.49 (0.34, 0.64)	0.91 (-1.66, 3.54)
Italy	33947 (32569, 35072)	60514 (50073, 71460)	78.26 (48.27, 109.71)	38.37 (36.84, 39.6)	43.54 (35.99, 51.56)	13.47 (-6.12, 34.63)	0.35 (0.23, 0.47)	0.01 (-0.32, 0.34)
United States of America	153168 (146891, 157265)	227242 (197022, 261375)	48.36 (28.89, 70.83)	47.64 (45.84, 48.85)	41.86 (36.15, 48.2)	-12.14 (-23.93, 1.47)	-0.56 (-0.77, -0.35)	-0.12 (-0.3, 0.07)
Bahamas	36 (33, 39)	108 (88, 131)	200.88 (139.25, 273.13)	23.01 (21.18, 25.05)	27.96 (22.83, 34.01)	21.48 (-2.81, 50.32)	0.78 (0.37, 1.19)	1.05 (-1.19, 3.33)
Croatia	2204 (2071, 2346)	4409 (3503, 5472)	100 (57.06, 151.11)	35.03 (32.89, 37.28)	50.47 (39.91, 62.58)	44.04 (13.09, 81.91)	1.47 (0.99, 1.96)	1.03 (0.22, 1.84)
Greece	3843 (3640, 4034)	7903 (6276, 9940)	105.64 (63.9, 159.35)	25.22 (23.89, 26.45)	33.21 (26.1, 42.09)	31.68 (3.42, 67.13)	0.78 (0.48, 1.08)	0.63 (0.11, 1.15)
Montenegro	135 (114, 155)	299 (245, 362)	121.63 (76.94, 180.05)	21.79 (18.27, 24.88)	30.47 (24.96, 36.76)	39.86 (13.03, 76.79)	1.37 (1.21, 1.53)	0.95 (-1.02, 2.95)
Hungary	6157 (5919, 6405)	9873 (8137, 11905)	60.34 (32.84, 92.43)	42.22 (40.57, 43.89)	52.24 (42.96, 62.9)	23.72 (1.74, 48.75)	0.62 (0.36, 0.89)	0.11 (-0.42, 0.64)
Oman	58 (40, 81)	251 (200, 333)	330.7 (166.19, 572.61)	9.01 (6.25, 12.26)	15.32 (12.63, 18.56)	70.05 (9.46, 166.22)	1.95 (1.79, 2.1)	1.52 (0.08, 2.99)
Northern Mariana Islands	5 (4, 6)	15 (12, 17)	192.52 (118.25, 281.79)	26.64 (21.39, 32.65)	28.86 (24.45, 33.06)	8.32 (-16.17, 33.14)	0.28 (0.09, 0.48)	0.01 (-6.15, 6.58)
Serbia	3075 (2524, 3443)	6789 (5452, 8446)	120.75 (72.06, 187.25)	27.33 (22.22, 30.62)	43.55 (35.07, 54.26)	59.38 (24.15, 108.86)	1.66 (1.39, 1.94)	1.27 (0.71, 1.83)
Spain	19094 (18176, 19858)	47432 (37493, 60137)	148.41 (96.81, 213.92)	35.28 (33.51, 36.64)	50.14 (39.34, 63.84)	42.11 (10.88, 81.28)	1.25 (0.93, 1.57)	0.56 (0.2, 0.91)
Bulgaria	3519 (3329, 3704)	6199 (4975, 7641)	76.18 (40.1, 119.58)	28.16 (26.65, 29.6)	43.84 (35.01, 54.21)	55.68 (23.38, 95.2)	1.87 (1.3, 2.44)	2.33 (1.7, 2.96)

Cook Islands	1 (1, 2)	3 (3, 4)	121.23 (69.89, 190.67)	10.95 (9.05, 12.91)	12.42 (10.26, 15.12)	13.43 (-13, 48.57)	0.39 (0.25, 0.53)	0.28 (-13.37, 16.1)
Greenland	13 (11, 15)	32 (26, 38)	150.27 (101.7, 209.66)	37.79 (33.7, 42.75)	47.44 (39.57, 55.72)	25.54 (2.46, 54.53)	0.76 (0.51, 1.01)	0.12 (-5.82, 6.43)
Romania	4862 (4676, 5043)	13039 (10746, 15541)	168.15 (122.12, 222.11)	17.29 (16.62, 17.92)	36.16 (29.52, 43.3)	109.17 (72.28, 151.69)	2.81 (2.3, 3.32)	1.9 (1.6, 2.2)
Chile	1502 (1426, 1567)	6006 (4800, 7551)	299.88 (217.1, 404.06)	15.58 (14.76, 16.27)	25.1 (20.08, 31.56)	61.04 (27.96, 102.62)	1.84 (1.36, 2.32)	2.24 (1.79, 2.69)
Trinidad and Tobago	157 (148, 165)	387 (296, 498)	146.49 (87.69, 221.79)	19.03 (17.9, 20.03)	21.07 (16.12, 27.06)	10.72 (-15.69, 43.9)	0.31 (0.13, 0.49)	0.37 (-0.91, 1.66)
Bahrain	25 (20, 29)	163 (123, 211)	555.13 (370.55, 849.47)	14.16 (11.83, 16.63)	16.96 (13.23, 21.08)	19.78 (-12.4, 69.84)	0.18 (-0.7, 1.07)	0.66 (-1.95, 3.33)
Turkey	5350 (4272, 6576)	18104 (14441, 22119)	238.4 (142.05, 364.53)	14.74 (11.65, 18)	20.56 (16.42, 25.01)	39.47 (0.99, 91.47)	1.07 (0.81, 1.32)	1.05 (0.88, 1.23)
Saint Kitts and Nevis	9 (9, 10)	20 (16, 23)	109.41 (70.77, 152.93)	25.44 (23.33, 27.59)	31.11 (26.32, 36.41)	22.29 (1.68, 45.47)	0.38 (-0.26, 1.02)	0.38 (-5.38, 6.49)
Belarus	3387 (3253, 3534)	5396 (4322, 6837)	59.3 (26.34, 103.39)	26.19 (25.17, 27.28)	34.12 (27.11, 43.36)	30.3 (3.52, 66.78)	0.87 (0.29, 1.46)	-0.48 (-1.01, 0.05)
North Macedonia	360 (329, 387)	1135 (898, 1416)	215.29 (143.85, 298.55)	19.27 (17.6, 20.75)	35.36 (28.19, 43.94)	83.49 (42.72, 131.24)	2.21 (2.01, 2.41)	1.93 (0.76, 3.1)
Antigua and Barbuda	9 (8, 9)	25 (21, 29)	188.47 (142.4, 239.3)	16 (14.6, 17.41)	25.58 (22.05, 29.43)	59.91 (35.09, 86.81)	1.79 (1.16, 2.42)	1.19 (-4.49, 7.2)
Portugal	4365 (4179, 4542)	10245 (8094, 12946)	134.72 (85.17, 195.64)	32 (30.61, 33.25)	45.29 (35.23, 57.72)	41.51 (10.5, 79.84)	1 (0.76, 1.25)	0.61 (0.18, 1.04)
Barbados	70 (64, 74)	195 (162, 233)	179.89 (130.38, 233.08)	23.81 (22.07, 25.34)	39.83 (33.03, 47.38)	67.24 (37.95, 98.91)	1.7 (1.42, 1.97)	1.4 (-1.04, 3.89)
Palau	2 (1, 2)	4 (3, 5)	146.73 (71.48, 244.67)	17.14 (13.28, 22.29)	19.94 (15.7, 24.69)	16.35 (-17.85, 59.96)	0.49 (0.38, 0.6)	0.43 (-11.8, 14.35)
Malaysia	1643 (1455, 1837)	7627 (5970, 9403)	364.26 (244.6, 511.68)	18.36 (16.04, 20.82)	29.57 (23.27, 36.5)	61.05 (19.01, 112.18)	1.6 (1.23, 1.98)	1.3 (0.98, 1.62)
Ukraine	21516 (20700, 22345)	23388 (19879, 27253)	8.7 (-8.36, 26.93)	30.04 (28.99, 31.17)	31.27 (26.56, 36.54)	4.09 (-12.35, 22.1)	0.19 (-0.45, 0.83)	-0.69 (-1.07, -0.31)

Jordan	192 (154, 236)	1258 (1037, 1520)	555.59 (373.28, 781.68)	14.11 (11.37, 17.35)	19.14 (15.96, 22.86)	35.67 (-1.31, 83.06)	1.07 (0.87, 1.28)	1.26 (0.53, 2)
Dominica	11 (10, 12)	18 (15, 22)	60.37 (31.5, 99.06)	15.39 (13.58, 17.28)	19.85 (16.29, 24.15)	28.98 (5.31, 60.02)	0.98 (0.7, 1.25)	0.99 (-4.72, 7.05)
Seychelles	11 (10, 12)	39 (34, 44)	256.78 (205.78, 314.31)	19.14 (17.36, 20.99)	35.7 (31.42, 40.58)	86.47 (60.12, 115.02)	2 (1.63, 2.37)	1.73 (-2.87, 6.55)
Kazakhstan	2466 (2338, 2596)	3378 (2927, 3878)	37 (18.56, 56.77)	19.24 (18.26, 20.23)	19.72 (17.18, 22.45)	2.49 (-11.04, 16.83)	0.43 (-0.14, 1)	-0.23 (-0.67, 0.22)
Bosnia and Herzegovina	723 (684, 766)	2058 (1619, 2571)	184.75 (122.46, 261)	18.01 (17.01, 19.13)	34.91 (27.54, 43.53)	93.8 (50.46, 144.52)	2.22 (1.87, 2.57)	2.53 (1.82, 3.25)
American Samoa	4 (3, 5)	10 (8, 12)	134.53 (81.91, 202.69)	18.61 (15.15, 21.73)	20.81 (17.56, 24.7)	11.83 (-12.71, 42.2)	0.62 (0.24, 1)	0.64 (-5.1, 6.73)
Niue	0 (0, 0)	0 (0, 1)	27.43 (-2.59, 65.19)	15.55 (12.92, 18.85)	20.4 (15.71, 26.06)	31.17 (-0.4, 72.37)	0.87 (0.69, 1.04)	0.86 (-26.13, 37.71)
Libya	267 (188, 386)	901 (658, 1180)	237.88 (86.92, 464.44)	14 (9.91, 20.12)	17 (12.41, 21.83)	21.4 (-32.58, 101.65)	0.6 (0.12, 1.08)	0.93 (0.18, 1.68)
Lebanon	337 (261, 427)	1555 (1259, 1991)	361.88 (226.15, 579.56)	15.46 (12.13, 19.4)	29.83 (24.07, 38.13)	92.91 (35.56, 179.73)	2.33 (2.24, 2.42)	3.06 (2.29, 3.83)
Argentina	8064 (7711, 8370)	18697 (14880, 23362)	131.86 (84.75, 189.09)	25.29 (24.12, 26.29)	34.68 (27.56, 43.36)	37.14 (8.81, 70.66)	0.88 (0.54, 1.22)	1.13 (0.92, 1.35)
Mauritius	76 (72, 82)	344 (278, 423)	351.53 (264.57, 459.57)	10.41 (9.73, 11.13)	19.77 (16.12, 24.16)	90.01 (54.25, 135.64)	2.25 (1.96, 2.55)	2.25 (0.58, 3.95)
Georgia	971 (892, 1057)	1096 (920, 1284)	12.94 (-6.86, 34.6)	15.66 (14.41, 17.02)	19.13 (16.02, 22.48)	22.19 (1.57, 45.69)	0.58 (0.13, 1.03)	0.94 (0.16, 1.72)
Uruguay	1363 (1294, 1430)	2162 (1734, 2690)	58.61 (27.41, 98.65)	35.04 (33.24, 36.73)	39.49 (31.56, 49.54)	12.72 (-9.93, 42.03)	0.27 (-0.2, 0.75)	0.39 (-0.4, 1.19)
Republic of Moldova	1113 (1068, 1166)	1691 (1472, 1927)	51.94 (32.8, 73.39)	24.62 (23.62, 25.77)	29.17 (25.39, 33.14)	18.49 (3.23, 35.2)	0.68 (-0.15, 1.52)	0.78 (0.06, 1.5)
Sri Lanka	596 (536, 658)	2546 (1896, 3346)	327.41 (205.31, 469.79)	5.61 (5.08, 6.18)	10.18 (7.63, 13.19)	81.38 (31.01, 139.44)	2.65 (2.28, 3.02)	1.96 (1.43, 2.49)
Middle SDI								
Armenia	503 (479, 528)	880 (738, 1035)	75.1 (46.28, 106.17)	18.13 (17.29, 19.1)	21.37 (17.85, 25.01)	17.86 (-1.69, 39.03)	0.61 (0.21, 1.01)	0.05 (-0.86, 0.96)

Thailand	4515 (4008, 5042)	17397 (12834, 22839)	285.35 (179.82, 419.72)	12.59 (11.12, 14.08)	17.18 (12.71, 22.51)	36.52 (-1, 83.23)	1.1 (0.8, 1.41)	0.23 (0, 0.47)
China	105911 (93808, 119021)	607900 (521805, 708420)	473.97 (369.16, 600.91)	12.52 (11.15, 14.03)	30.55 (26.37, 35.5)	144.07 (99.97, 195.49)	2.99 (2.71, 3.26)	3.31 (3.08, 3.55)
Panama	176 (164, 188)	772 (599, 979)	337.54 (237.28, 461.11)	11.83 (10.94, 12.64)	18.61 (14.45, 23.57)	57.29 (21.52, 101.72)	1.72 (1.41, 2.04)	1.83 (0.91, 2.75)
Equatorial Guinea	12 (8, 17)	73 (45, 108)	484.55 (201.95, 827.05)	6.38 (4.39, 8.6)	15.6 (9.84, 22.43)	144.56 (28.53, 288.91)	3.22 (3.07, 3.37)	3.63 (0.66, 6.7)
Jamaica	253 (235, 268)	772 (622, 960)	205.77 (143.7, 281.99)	14.03 (13.09, 14.89)	25.95 (20.82, 32.26)	85.03 (46.98, 130.77)	2.47 (1.73, 3.21)	2.48 (1.55, 3.43)
Azerbaijan	694 (642, 748)	1558 (1280, 1911)	124.57 (84.1, 178.37)	13.04 (12.07, 14.02)	16.32 (13.28, 20.08)	25.13 (0.62, 54.17)	0.68 (0.14, 1.22)	0.76 (0.29, 1.24)
Albania	220 (204, 235)	630 (470, 828)	186.73 (112.4, 278.97)	10.53 (9.74, 11.29)	15.15 (11.4, 19.9)	43.93 (7.2, 89.93)	1.16 (0.79, 1.54)	1.84 (1.07, 2.62)
Costa Rica	256 (239, 272)	1496 (1161, 1902)	484.1 (351.39, 649.23)	14.61 (13.58, 15.5)	29.18 (22.72, 37.17)	99.69 (54.4, 156.28)	2.62 (2.45, 2.78)	3 (2.3, 3.7)
South Africa	2265 (1953, 2731)	5566 (5002, 6289)	145.74 (117.68, 182.06)	11.03 (9.38, 13.51)	12.88 (11.62, 14.5)	16.76 (2.11, 36.12)	1.06 (0.63, 1.48)	-0.06 (-0.34, 0.23)
Tunisia	440 (355, 523)	1801 (1300, 2441)	309.1 (180.7, 489.44)	9.16 (7.39, 10.97)	14.49 (10.54, 19.5)	58.11 (9.36, 127.36)	1.58 (1.46, 1.71)	1.51 (0.87, 2.15)
Iraq	632 (460, 868)	2647 (2045, 3379)	319 (173.17, 531.43)	8.02 (5.84, 10.85)	11.13 (8.73, 13.83)	38.71 (-7.37, 107.02)	1.15 (0.95, 1.36)	1.61 (1.22, 2)
Iran (Islamic Republic of)	2162 (1774, 2613)	10183 (9381, 11029)	370.95 (270.66, 498.98)	8.35 (6.8, 10.06)	13.88 (12.76, 15.07)	66.33 (31.46, 112.48)	1.82 (1.59, 2.06)	1.79 (1.57, 2.01)
Saint Lucia	13 (12, 14)	38 (32, 45)	202.96 (152.87, 263.09)	14.68 (13.56, 15.75)	17.96 (15.15, 21.2)	22.33 (1.98, 46.56)	0.69 (0.46, 0.93)	0.59 (-3.63, 5)
Turkmenistan	187 (178, 197)	354 (284, 439)	88.92 (51.83, 136.45)	8.98 (8.56, 9.44)	9.19 (7.47, 11.33)	2.31 (-17.22, 26.02)	0.09 (-0.73, 0.92)	-0.5 (-1.42, 0.43)
Grenada	13 (12, 14)	30 (27, 33)	137.94 (108.42, 170.44)	17.38 (15.84, 19.04)	27.86 (25.13, 30.69)	60.27 (40.7, 81.55)	1.79 (1.19, 2.39)	1.48 (-3.21, 6.39)
Cuba	2400 (2278, 2504)	6569 (5388, 7975)	173.69 (122.99, 230.71)	23.35 (22.18, 24.36)	34.57 (28.25, 42.05)	48.06 (20.36, 79.25)	1.51 (1.11, 1.92)	0.82 (0.35, 1.28)

Fiji	37 (30, 45)	95 (75, 120)	160.69 (90.45, 258.57)	10.24 (8.4, 12.33)	13.52 (10.86, 16.56)	31.95 (-2.73, 75.98)	0.94 (0.75, 1.13)	1.06 (-0.89, 3.05)
Indonesia	9509 (7236, 11371)	39110 (26895, 49833)	311.29 (221.04, 401.84)	9.53 (7.35, 11.28)	18.53 (12.63, 23.42)	94.49 (51.43, 140.82)	2.31 (2.27, 2.36)	1.87 (1.76, 1.99)
Egypt	1804 (1655, 1963)	6520 (4676, 9006)	261.48 (156.41, 406.98)	5.93 (5.4, 6.42)	9.78 (7.07, 13.43)	64.9 (17.68, 131)	1.61 (1.51, 1.71)	1.45 (1.19, 1.71)
Gabon	81 (53, 121)	166 (119, 210)	106.01 (31.28, 203.9)	14.75 (9.83, 21.87)	16.38 (12.14, 20.32)	11.05 (-27.57, 60.44)	0.4 (0.29, 0.52)	-0.01 (-1.52, 1.53)
Algeria	890 (719, 1084)	3407 (2668, 4282)	283.04 (177.02, 412.91)	7.81 (6.43, 9.33)	10.52 (8.35, 13.05)	34.71 (-1.6, 78.36)	0.98 (0.83, 1.13)	0.74 (0.27, 1.21)
Mexico	3351 (3227, 3429)	17470 (15042, 20060)	421.35 (347.55, 498.06)	7.88 (7.52, 8.1)	14.92 (12.88, 17.11)	89.22 (63.09, 116.76)	2.13 (1.52, 2.74)	2.27 (2.09, 2.45)
Peru	1262 (1082, 1457)	6805 (5101, 8908)	439.15 (290.48, 642.18)	10.6 (9.06, 12.25)	21.24 (15.91, 27.77)	100.44 (45.16, 175.91)	2.64 (2.1, 3.18)	2.76 (2.47, 3.04)
Samoa	10 (8, 12)	18 (15, 23)	80.37 (34.76, 141.41)	11.82 (9.84, 14.3)	12.76 (10.27, 15.68)	7.93 (-18.19, 41.58)	0.23 (0.09, 0.38)	0.19 (-4.27, 4.86)
Ecuador	459 (431, 486)	2850 (2269, 3613)	521.34 (393.34, 692.33)	8.77 (8.19, 9.31)	19.25 (15.35, 24.31)	119.41 (74.7, 178.83)	2.76 (2.46, 3.07)	3.09 (2.64, 3.54)
Brazil	10556 (10181, 10886)	41935 (39320, 43999)	297.26 (274.93, 315.5)	12.12 (11.58, 12.52)	17.77 (16.64, 18.65)	46.61 (38.65, 53.14)	1.4 (1.27, 1.53)	1.39 (1.26, 1.51)
Paraguay	161 (143, 180)	955 (731, 1218)	495.14 (336.32, 685.73)	7.3 (6.48, 8.2)	17.37 (13.38, 22.12)	137.95 (75.23, 213.86)	3.09 (2.74, 3.44)	3.07 (2.29, 3.85)
Suriname	37 (33, 40)	128 (105, 152)	247.46 (184.47, 322.09)	14.32 (12.95, 15.51)	21.61 (17.85, 25.57)	50.96 (24.45, 82.62)	1.14 (-0.09, 2.39)	1.38 (-0.73, 3.54)
Tonga	4 (3, 4)	7 (5, 8)	83.56 (41.57, 139.47)	6.65 (5.48, 7.82)	8.28 (6.45, 10.46)	24.57 (-3.04, 62.26)	0.88 (0.63, 1.13)	0.7 (-6.48, 8.43)
Botswana	60 (45, 79)	247 (171, 334)	312.87 (177.61, 477.26)	11.09 (8.57, 14.35)	18.75 (13.48, 24.55)	69.17 (17.96, 128.74)	1.83 (1.64, 2.02)	1.35 (0, 2.73)
Colombia	2046 (1951, 2128)	9046 (7010, 11553)	342.13 (239.64, 462.61)	11.8 (11.19, 12.35)	17.15 (13.28, 21.89)	45.29 (11.87, 85.84)	1.21 (1.03, 1.39)	1.58 (1.35, 1.82)
Uzbekistan	1070 (1017, 1120)	2404 (2026, 2826)	124.63 (89.34, 164.59)	8.85 (8.4, 9.26)	12.82 (11.1, 14.7)	44.84 (24.29, 66.21)	1.64 (1.32, 1.97)	0.22 (-0.13, 0.57)

Saint Vincent and the Grenadines	10 (10, 11)	26 (23, 30)	151.54 (116.57, 196.05)	14.5 (13.31, 15.67)	19.66 (17.2, 22.56)	35.58 (17.14, 59.06)	1.13 (0.83, 1.42)	0.76 (-4.07, 5.84)
Tokelau	0 (0, 0)	0 (0, 0)	26.9 (-5.58, 68.86)	10.88 (8.65, 13.52)	14.11 (10.54, 18.35)	29.72 (-3.53, 71.93)	0.9 (0.83, 0.97)	0.99 (-36.98, 61.86)
Philippines	4663 (4155, 5204)	15220 (12313, 18708)	226.39 (155.68, 324.61)	14.93 (13.31, 16.67)	18.93 (15.42, 23.14)	26.82 (0.28, 63.12)	0.99 (0.74, 1.25)	0.55 (0.37, 0.72)
Syrian Arab Republic	343 (265, 436)	1028 (744, 1375)	199.84 (91.56, 355.27)	6.2 (4.81, 7.87)	8.54 (6.26, 11.28)	37.73 (-10.6, 108.95)	1.45 (0.77, 2.14)	0.75 (0.25, 1.24)
Guyana	55 (47, 62)	116 (90, 147)	111.77 (58.34, 176.81)	14.5 (12.67, 16.43)	18.71 (14.85, 23.58)	29.05 (-1.67, 66.27)	1.03 (0.66, 1.4)	1.14 (-0.66, 2.97)
Nauru	1 (1, 1)	1 (1, 1)	14.48 (-18.62, 58.13)	21.14 (14.09, 30.31)	21.43 (14.78, 27.76)	1.36 (-25.89, 36.34)	0.01 (-0.17, 0.19)	-0.18 (-14.76, 16.88)
Viet Nam	3981 (3207, 4814)	24869 (19211, 30956)	524.67 (360.79, 730.14)	9.93 (8.03, 11.94)	26.36 (20.58, 32.37)	165.36 (96.64, 247.98)	3.51 (3.4, 3.62)	3.82 (3.6, 4.04)
Namibia	41 (32, 50)	133 (103, 172)	226.02 (134.92, 353.45)	5.81 (4.62, 7.05)	9.74 (7.69, 12.25)	67.79 (23.23, 129.13)	1.83 (1.51, 2.15)	1.52 (-0.4, 3.47)
Low middle SDI								
Venezuela (Bolivarian Republic of)	1060 (1013, 1110)	5361 (4064, 7020)	405.79 (279.67, 564.91)	10.91 (10.33, 11.44)	18.58 (14.14, 24.22)	70.32 (28.6, 122.17)	1.63 (1.27, 1.99)	1.73 (1.41, 2.05)
Mongolia	100 (82, 120)	256 (199, 333)	154.79 (89.87, 243.67)	9.47 (7.79, 11.27)	11.09 (8.77, 14.12)	17.08 (-11.67, 55.33)	0.42 (0.25, 0.6)	0.21 (-1.35, 1.79)
Belize	8 (7, 8)	41 (35, 47)	422.72 (341.18, 516.9)	8.39 (7.55, 9.18)	14.64 (12.76, 16.95)	74.61 (47.92, 106.02)	1.84 (1.3, 2.37)	1.99 (-1.51, 5.6)
Kyrgyzstan	430 (403, 460)	472 (412, 541)	9.8 (-5.67, 26.9)	13.94 (13.07, 14.92)	10.52 (9.21, 11.99)	-24.57 (-35.05, -12.82)	-1 (-1.33, -0.67)	-1.55 (-2.33, -0.77)
Dominican Republic	306 (270, 343)	1579 (1133, 2067)	416.74 (267.87, 598.72)	8.35 (7.34, 9.4)	17.08 (12.37, 22.31)	104.43 (44.5, 174.99)	2.78 (2.31, 3.24)	2.95 (2.4, 3.5)
Tuvalu	1 (1, 1)	1 (1, 2)	78.62 (32.05, 143.19)	10.87 (8.74, 13.02)	13.03 (9.6, 16.87)	19.9 (-11.3, 61.12)	0.61 (0.53, 0.69)	0.47 (-15.24, 19.08)
Palestine	149 (101, 206)	616 (521, 716)	314.4 (188.11, 539.42)	17.05 (11.58, 23.6)	26.12 (22.15, 30.27)	53.19 (6.36, 135.9)	1.59 (1.37, 1.82)	1.1 (0.25, 1.95)
Micronesia (Federated States of)	6 (4, 7)	11 (7, 14)	90.13 (23.58, 166.99)	11.95 (9.18, 14.98)	15.36 (10.93, 20)	28.51 (-14.04, 76.61)	0.84 (0.74, 0.94)	0.72 (-4.73, 6.48)

Eswatini	29 (22, 38)	80 (53, 111)	177.87 (94.58, 293.25)	10.38 (7.92, 13.34)	14.4 (9.82, 19.67)	38.72 (-0.8, 92.21)	1.12 (0.93, 1.31)	1.44 (-0.56, 3.47)
El Salvador	170 (158, 182)	844 (644, 1096)	396.41 (272.85, 546.44)	5.65 (5.22, 6.06)	14.12 (10.72, 18.33)	150.1 (86.44, 227.7)	3.28 (2.98, 3.57)	2.93 (2.18, 3.68)
Congo	124 (82, 166)	303 (219, 405)	144.55 (59.25, 278.84)	11.78 (8.15, 15.41)	11.94 (9.05, 15.43)	1.41 (-32.26, 50.81)	0.13 (-0.04, 0.29)	-0.24 (-1.5, 1.03)
Bolivia (Plurinational State of)	301 (214, 377)	1439 (991, 1911)	378.82 (261.76, 526.71)	9.68 (6.99, 12.1)	16.89 (11.6, 22.44)	74.58 (33.91, 123.9)	1.94 (1.82, 2.07)	1.39 (0.74, 2.03)
India	24320 (21284, 27797)	95112 (79687, 110631)	291.08 (202.87, 374.86)	5.59 (4.86, 6.39)	8.59 (7.22, 9.94)	53.61 (19.56, 85.85)	1.65 (1.38, 1.91)	1.04 (0.88, 1.19)
Maldives	9 (6, 11)	41 (34, 49)	373.66 (233.82, 634.2)	10.01 (7.4, 12.5)	13.8 (11.28, 16.4)	37.87 (-1.49, 98.72)	1.03 (0.82, 1.25)	0.54 (-4.09, 5.39)
Democratic People's Republic of Korea	2329 (1735, 3057)	4986 (3708, 6464)	114.08 (40.82, 207.36)	13.93 (10.79, 17.85)	15.42 (11.47, 19.91)	10.73 (-25.69, 55.53)	0.28 (0.14, 0.42)	0.31 (-0.05, 0.66)
Ghana	388 (315, 477)	1495 (1165, 1901)	285.07 (178.65, 426.58)	6.37 (5.28, 7.72)	9.51 (7.57, 11.94)	49.36 (9.4, 101.12)	1.42 (1.19, 1.64)	1.39 (0.9, 1.87)
Morocco	905 (726, 1060)	3213 (2395, 4072)	255.18 (156, 373.61)	6.66 (5.24, 7.81)	10.34 (7.7, 13.02)	55.25 (12.63, 107.74)	1.53 (1.35, 1.7)	1.13 (0.7, 1.56)
Marshall Islands	2 (2, 2)	5 (3, 6)	146.77 (78.43, 234.33)	11.48 (9.49, 13.88)	13.71 (10.51, 17.43)	19.48 (-11.02, 58.71)	0.55 (0.41, 0.7)	0.68 (-7.59, 9.7)
Tajikistan	324 (299, 354)	551 (443, 674)	69.66 (34.39, 112.36)	10.7 (9.85, 11.68)	11.62 (9.5, 14.05)	8.64 (-12.49, 33.88)	0.58 (0.13, 1.04)	-0.2 (-0.81, 0.41)
Kiribati	4 (3, 5)	8 (6, 10)	82.34 (28.25, 170.99)	11.2 (9.11, 13.4)	11.45 (8.7, 15.08)	2.22 (-26.94, 48.62)	0.08 (0, 0.16)	-0.22 (-6.54, 6.53)
Guatemala	192 (171, 216)	1290 (1020, 1619)	572.38 (418.15, 761.11)	5.41 (4.83, 6.06)	11.51 (9.18, 14.4)	112.81 (65.08, 170.07)	2.67 (2.34, 3)	2.8 (2.15, 3.45)
Cabo Verde	11 (10, 12)	56 (45, 66)	405.33 (302.41, 509.12)	4.77 (4.32, 5.27)	13.39 (10.67, 15.73)	180.58 (121.11, 237.29)	3.75 (3.27, 4.23)	2.5 (-0.6, 5.71)
Myanmar	2186 (1621, 2970)	6913 (5085, 9011)	216.27 (107.19, 335.87)	9.23 (7.07, 12.61)	15.03 (11.1, 19.26)	62.76 (6.01, 120.69)	1.67 (1.5, 1.84)	1.51 (1.27, 1.76)
Nicaragua	114 (96, 130)	689 (571, 820)	506.97 (372.67, 675.17)	7.33 (6.07, 8.64)	16.17 (13.44, 19.03)	120.53 (72.03, 188.25)	2.86 (2.54, 3.17)	2.77 (1.93, 3.62)

Sudan	484 (366, 716)	1564 (1105, 2336)	222.88 (125, 353.17)	5.17 (3.96, 7.75)	8.22 (5.99, 12.28)	58.77 (14.66, 118.75)	1.6 (1.57, 1.64)	1.67 (1.17, 2.18)
Nigeria	2586 (1873, 3447)	7084 (5312, 8958)	173.93 (102.71, 287.09)	6.32 (4.66, 8.32)	8.95 (6.87, 11.02)	41.61 (6.51, 95.25)	1.17 (1.08, 1.25)	1.38 (1.13, 1.62)
Timor-Leste	22 (16, 30)	112 (76, 144)	405.22 (230.01, 629.87)	7.6 (5.71, 10.13)	13.91 (9.69, 17.85)	82.96 (22.44, 164.08)	2.12 (1.99, 2.25)	2.48 (0.38, 4.62)
Kenya	428 (275, 528)	1784 (1423, 2206)	316.9 (227.81, 457.27)	5.26 (3.39, 6.51)	8.24 (6.68, 10.05)	56.74 (25.61, 108.6)	1.5 (1.34, 1.67)	1.47 (0.96, 1.99)
Lesotho	61 (47, 87)	146 (104, 194)	140.57 (54.4, 249.98)	6.5 (5.09, 9.32)	12.01 (8.83, 15.54)	84.69 (21.94, 165.57)	2.26 (2.05, 2.48)	2.98 (1.32, 4.67)
Zambia	292 (221, 374)	924 (671, 1201)	216.08 (124.6, 328.53)	10.34 (7.9, 13.05)	13.62 (10.14, 17.44)	31.74 (-3.65, 74.19)	0.91 (0.85, 0.96)	0.86 (0.2, 1.52)
Sao Tome and Principe	6 (5, 7)	16 (12, 22)	172.33 (91.73, 277.1)	9.7 (7.93, 11.41)	16.46 (12.02, 22.22)	69.64 (21.56, 134.27)	1.91 (1.77, 2.05)	1.67 (-2.9, 6.46)
Honduras	112 (91, 130)	574 (398, 812)	412.63 (266.58, 609.04)	5.25 (4.19, 6.14)	9.67 (6.74, 13.58)	84.28 (31.89, 155.18)	2.26 (2.03, 2.49)	1.08 (0.18, 1.98)
Mauritania	83 (62, 102)	173 (130, 220)	108.44 (49.1, 207.09)	8.55 (6.41, 10.5)	8.84 (6.8, 11.08)	3.47 (-24.37, 50.37)	0.15 (0.06, 0.25)	0 (-1.46, 1.47)
Lao People's Democratic Republic	222 (149, 299)	656 (465, 874)	194.96 (100.8, 313.91)	10.44 (7.19, 13.87)	14.9 (10.64, 19.54)	42.73 (-1.73, 92.97)	1.22 (1.15, 1.3)	0.94 (0.15, 1.73)
Cameroon	384 (305, 470)	1267 (933, 1684)	229.5 (128.28, 356.87)	9.11 (7.26, 11.04)	11.18 (8.58, 14.61)	22.74 (-12.39, 66.31)	0.71 (0.6, 0.81)	0.72 (0.19, 1.26)
Vanuatu	5 (4, 7)	17 (13, 23)	232.56 (138.69, 364.32)	7.97 (5.86, 10.37)	10.1 (7.56, 13.08)	26.7 (-7.41, 71.43)	0.81 (0.44, 1.19)	0.64 (-3.98, 5.48)
Bangladesh	2165 (1600, 2864)	7167 (4965, 10209)	231.07 (125.53, 379.96)	4.66 (3.4, 6.23)	5.63 (3.9, 8)	20.89 (-16.11, 70.6)	0.84 (0.3, 1.38)	0.47 (0.22, 0.72)
Zimbabwe	413 (361, 470)	935 (709, 1183)	126.08 (65.35, 195.45)	10.66 (9.37, 12.02)	13.76 (10.57, 17.23)	29.06 (-4.18, 67.54)	1.01 (0.67, 1.35)	1.13 (0.52, 1.74)
Angola	301 (209, 433)	1083 (829, 1389)	259.98 (143.61, 436.21)	7.85 (5.54, 10.96)	10.02 (8.06, 12.55)	27.67 (-10.87, 81.46)	0.83 (0.67, 0.98)	0.84 (0.2, 1.49)
Cambodia	429 (328, 554)	1983 (1563, 2416)	362.37 (232.56, 514.06)	9.33 (7.36, 12.07)	16.67 (13.29, 20)	78.77 (29.83, 129.94)	2 (1.96, 2.04)	1.85 (1.33, 2.37)

Djibouti	12 (8, 17)	70 (49, 97)	461.26 (262.39, 733.43)	9 (6.37, 12.11)	11.92 (9.06, 15.8)	32.51 (-10.13, 88.51)	0.94 (0.64, 1.24)	1.02 (-2.01, 4.14)
Bhutan	11 (6, 16)	43 (26, 59)	298.52 (187.66, 456.34)	4.6 (2.63, 6.54)	8.03 (4.91, 10.87)	74.62 (27.94, 135.51)	1.93 (1.85, 2.01)	1.53 (-1.89, 5.08)
Comoros	17 (10, 23)	43 (31, 55)	156.02 (64.3, 371.42)	7.73 (4.71, 10.3)	8.96 (6.63, 11.43)	15.92 (-24.17, 98.7)	0.58 (0.28, 0.88)	0.44 (-2.56, 3.54)
Low SDI								
Pakistan	3111 (2689, 3576)	10140 (8100, 12938)	225.95 (149.25, 343.09)	5.49 (4.74, 6.33)	9.14 (7.33, 11.63)	66.43 (28.2, 124.96)	1.76 (1.67, 1.85)	1.87 (1.68, 2.06)
Haiti	328 (215, 404)	779 (536, 1065)	137.69 (58.97, 252.27)	10.23 (6.74, 12.43)	11.45 (7.97, 15.43)	11.91 (-24.53, 62.45)	0.41 (0.35, 0.47)	0.42 (-0.26, 1.11)
Rwanda	255 (182, 324)	549 (424, 704)	115.15 (50.29, 254.25)	8.75 (6.45, 11.03)	9.32 (7.43, 11.66)	6.59 (-23.07, 66.57)	0.14 (-0.09, 0.36)	-0.44 (-1.25, 0.37)
United Republic of Tanzania	844 (698, 1035)	2457 (1926, 3175)	190.95 (127.07, 265.09)	7.95 (6.62, 9.6)	10.1 (8.13, 12.7)	27.15 (1.78, 55.74)	0.79 (0.64, 0.94)	0.92 (0.51, 1.32)
Nepal	334 (223, 457)	1249 (891, 1727)	273.6 (167.16, 412.1)	3.62 (2.35, 5.05)	5.88 (4.22, 8.15)	62.7 (18.11, 122.17)	1.73 (1.57, 1.88)	1.21 (0.57, 1.86)
Togo	85 (71, 102)	280 (205, 368)	227.89 (144.86, 333.12)	7.13 (6, 8.46)	8.22 (6.14, 10.53)	15.33 (-12.22, 49.68)	0.49 (0.38, 0.6)	0.38 (-0.74, 1.51)
Yemen	276 (178, 408)	996 (736, 1350)	260.84 (121.87, 492.55)	5.66 (3.65, 8.25)	7.39 (5.56, 9.99)	30.58 (-17.71, 108.99)	0.99 (0.85, 1.12)	1.42 (0.74, 2.1)
Cote d'Ivoire	336 (260, 432)	946 (724, 1199)	181.2 (100.32, 275.04)	8.85 (6.93, 11.3)	9.59 (7.65, 11.87)	8.34 (-18.49, 39.05)	0.21 (0.09, 0.33)	-0.1 (-0.68, 0.47)
Solomon Islands	14 (9, 20)	41 (26, 56)	199.35 (111.99, 308.86)	9.32 (6.21, 13.17)	12.36 (8.44, 16.09)	32.58 (-2.65, 79.34)	1.06 (0.89, 1.22)	0.88 (-3.04, 4.97)
Uganda	424 (345, 501)	1737 (1348, 2150)	309.65 (205.61, 435.7)	6.75 (5.57, 7.86)	12.31 (9.77, 14.83)	82.34 (39.56, 131.27)	2.09 (1.91, 2.26)	2.17 (1.67, 2.67)
Gambia	16 (12, 20)	64 (46, 85)	298.75 (156, 497.69)	4.73 (3.71, 5.89)	6.84 (4.99, 9.15)	44.47 (-4.95, 109.03)	1.33 (0.87, 1.79)	1.04 (-1.63, 3.79)
Eritrea	67 (47, 89)	279 (211, 365)	318.24 (187.39, 532.82)	6.5 (4.66, 8.72)	10.34 (8.12, 13.21)	58.99 (11.71, 132.15)	1.62 (1.44, 1.79)	1.27 (-0.26, 2.82)
Madagascar	352 (283, 417)	802 (582, 1078)	127.65 (59.94, 215.91)	6.8 (5.5, 7.93)	7.33 (5.41, 9.73)	7.78 (-23.24, 45.79)	0.28 (0.04, 0.53)	-0.08 (-0.68, 0.52)

Papua New Guinea	121 (82, 162)	382 (273, 515)	216.07 (135.95, 327.91)	6.55 (4.4, 8.74)	8.06 (5.88, 10.54)	23.13 (-6.05, 64.38)	0.71 (0.65, 0.77)	0.63 (-0.34, 1.61)
Senegal	233 (186, 278)	635 (499, 800)	172.22 (104.98, 256.13)	7.51 (6.1, 8.88)	8.92 (7.17, 11.08)	18.85 (-7.81, 52.39)	0.46 (-0.02, 0.95)	0.65 (-0.1, 1.41)
Malawi	176 (147, 204)	442 (335, 562)	151.19 (83.14, 235.52)	4.74 (4, 5.4)	6.27 (4.87, 7.8)	32.11 (-2.08, 72.46)	0.96 (0.81, 1.12)	0.84 (-0.02, 1.71)
Democratic Republic of the Congo	1009 (785, 1352)	2194 (1480, 3261)	117.38 (37.52, 216.21)	6.74 (5.22, 9.2)	6.36 (4.24, 9.58)	-5.65 (-39.14, 34.17)	-0.26 (-0.4, -0.13)	-0.39 (-0.79, 0.01)
Liberia	71 (59, 86)	132 (89, 189)	84.61 (26.24, 159.69)	6.67 (5.56, 7.99)	6.84 (4.66, 9.71)	2.42 (-29.68, 41.95)	-0.04 (-0.27, 0.2)	0.58 (-0.96, 2.14)
South Sudan	234 (139, 385)	367 (236, 552)	56.6 (12.59, 133.21)	10.09 (6.1, 16.37)	9.92 (6.56, 14.66)	-1.64 (-27.29, 42)	-0.07 (-0.14, -0.01)	0.06 (-0.8, 0.93)
Guinea-Bissau	35 (24, 44)	65 (49, 83)	88.74 (34.95, 164.35)	8.62 (6.11, 10.88)	9.32 (7.08, 11.68)	8.18 (-21.02, 48.65)	0.24 (0.17, 0.31)	0.29 (-1.83, 2.46)
Benin	125 (105, 148)	360 (283, 465)	189.1 (117.87, 268.32)	6.38 (5.4, 7.5)	7.78 (6.25, 9.85)	21.98 (-5.55, 52.21)	0.69 (0.61, 0.77)	0.65 (-0.28, 1.6)
Sierra Leone	113 (89, 137)	244 (185, 316)	116.4 (58.16, 192.35)	6.03 (4.8, 7.3)	7.09 (5.48, 9.11)	17.53 (-12.72, 57.01)	0.6 (0.44, 0.75)	0.92 (-0.26, 2.11)
Afghanistan	563 (331, 870)	1168 (749, 1606)	107.44 (45.38, 204.08)	7.91 (4.89, 11.93)	8.69 (6.04, 11.55)	9.91 (-23.48, 56.53)	0.36 (0.2, 0.53)	0.39 (-0.14, 0.92)
Ethiopia	1500 (1079, 2323)	3199 (2401, 4461)	113.21 (54.11, 242.15)	7.32 (5.14, 11.44)	7.7 (5.78, 10.75)	5.15 (-22.77, 55.27)	0.16 (0.07, 0.25)	-0.37 (-0.74, 0.01)
Guinea	179 (151, 210)	390 (296, 510)	118.48 (56.84, 205.77)	5.59 (4.72, 6.54)	7.3 (5.56, 9.43)	30.55 (-6.24, 80.86)	0.95 (0.87, 1.02)	1.1 (0.2, 2.01)
Mozambique	269 (221, 321)	900 (649, 1189)	234.45 (146, 350.24)	4.9 (4.09, 5.74)	8.73 (6.43, 11.37)	78.34 (31.22, 136.33)	2.07 (1.84, 2.3)	2.63 (1.88, 3.39)
Burundi	187 (148, 241)	335 (237, 484)	78.61 (15.37, 185.65)	7.96 (6.36, 10.07)	7.35 (5.32, 10.43)	-7.72 (-39.91, 43.72)	-0.3 (-0.42, -0.17)	-0.56 (-1.49, 0.38)
Central African Republic	85 (63, 109)	138 (98, 192)	61.51 (13.38, 124.04)	7.31 (5.65, 9.36)	6.31 (4.65, 8.68)	-13.7 (-38.16, 16.59)	-0.53 (-0.65, -0.42)	-0.4 (-1.82, 1.05)
Mali	260 (219, 303)	675 (529, 853)	159.79 (99.22, 243.16)	6.49 (5.52, 7.53)	8.11 (6.43, 10.13)	25 (-2.36, 61.09)	0.8 (0.54, 1.07)	0.66 (-0.09, 1.42)
Burkina Faso	237 (189, 298)	641 (501, 816)	170.14 (117.25, 241.48)	5.77 (4.67, 7.24)	7.42 (5.92, 9.38)	28.74 (5.51, 59.86)	0.94 (0.83, 1.05)	0.98 (0.19, 1.78)

Chad	144 (111, 180)	391 (300, 513)	172.54 (109.2, 250.33)	5.21 (4.04, 6.5)	7.32 (5.68, 9.41)	40.45 (10.27, 77.22)	1.18 (1.08, 1.28)	1.27 (0.33, 2.22)
Niger	141 (108, 186)	406 (295, 556)	187.58 (116.62, 279.16)	5.31 (4.07, 6.91)	5.63 (4.19, 7.56)	6.11 (-17.81, 34.83)	0.25 (0.13, 0.38)	0.08 (-0.86, 1.04)
Somalia	133 (91, 203)	330 (206, 615)	147.19 (58.61, 275.44)	5.25 (3.67, 7.9)	4.95 (3.13, 9.24)	-5.68 (-37.07, 40.86)	-0.18 (-0.26, -0.1)	0.04 (-1.03, 1.12)

Notes:

Age-standardized incidence rate is computed by direct standardization with global standard population in GBD 2019.

AAPC: Average annual percentage change, which is calculated by joinpoint software, which is described the trend of colorectal cancer incidence.

Net drifts are estimates derived from the age-period-cohort model and denotes overall annual percent change in morbidity.

Parenthesis for all GBD health estimate indicates 95% uncertainty intervals; parenthesis for net drift indicates 95% confidence intervals.

SDI= Socio-demographic Index; APC= age-period-cohort

Table S2. The ASIR and local draft of age groups by SDI quintiles from 1990 to 2019.

			5-9 years	10-14 years	15-19 years	20-24 years	25-29 years	30-34 years	35-39 years	40-44 years	45-49 years	50-54 years	55-59 years	60-64 years	65-69 years	70-74 years	75-79 years	80-84 years	85-89 years	90-94 years	95 plus years
global	ASIR (95% UI)	both	0.06 (0.05, 0.07)	0.06 (0.05, 0.07)	0.26 (0.24, 0.28)	0.56 (0.52, 0.59)	1.09 (1.04, 1.15)	2.37 (2.28, 2.45)	4.62 (4.49, 4.75)	8.64 (8.46, 8.83)	16.25 (15.97, 16.54)	30.61 (30.17, 31.05)	51.69 (51.04, 52.34)	81.45 (80.52, 82.40)	121.91 (120.54, 123.30)	174.47 (172.13, 176.83)	237.44 (234.09, 240.84)	298.86 (294.26, 303.54)	367.66 (361.08, 374.36)	366.57 (357.42, 375.95)	480.45 (462.11, 499.52)
		male	0.05 (0.04, 0.06)	0.05 (0.04, 0.06)	0.25 (0.22, 0.27)	0.51 (0.48, 0.55)	1.03 (0.98, 1.09)	2.32 (2.23, 2.41)	4.75 (4.61, 4.90)	9.09 (8.87, 9.31)	17.67 (17.33, 18.02)	34.18 (33.64, 34.73)	61.48 (60.65, 62.33)	101.29 (100.03, 102.55)	155.84 (153.95, 157.75)	224.59 (221.31, 227.91)	307.12 (302.34, 311.97)	384.44 (377.75, 391.26)	476.34 (466.23, 486.67)	448.59 (433.48, 464.22)	567.14 (534.48, 601.80)
		female	0.08 (0.06, 0.09)	0.09 (0.07, 0.10)	0.28 (0.25, 0.31)	0.63 (0.58, 0.68)	1.21 (1.14, 1.28)	2.47 (2.37, 2.59)	4.52 (4.36, 4.68)	8.21 (7.99, 8.44)	14.80 (14.47, 15.13)	26.95 (26.46, 27.46)	41.91 (41.23, 42.59)	62.40 (61.47, 63.35)	90.82 (89.49, 92.17)	130.83 (128.59, 133.12)	180.51 (177.29, 183.78)	233.20 (228.75, 237.72)	291.08 (284.89, 297.40)	303.33 (295.06, 311.83)	403.52 (387.86, 419.82)
	Local Drift (%) (95% CI)	both	-0.66 (-1.97, 0.67)	-0.10 (-0.99, 0.80)	0.39 (-0.14, 0.92)	0.82 (0.47, 1.18)	1.10 (0.86, 1.35)	1.19 (1.01, 1.37)	1.14 (1.00, 1.28)	1.12 (1.00, 1.23)	1.11 (1.02, 1.20)	1.04 (0.97, 1.12)	0.91 (0.85, 0.98)	0.76 (0.70, 0.82)	0.53 (0.47, 0.58)	0.33 (0.27, 0.38)	0.27 (0.21, 0.33)	0.26 (0.18, 0.33)	0.41 (0.30, 0.51)	0.61 (0.42, 0.80)	0.93 (0.51, 1.36)
		male	-0.60 (-2.07, 0.90)	0.08 (-0.94, 1.10)	0.73 (0.15, 1.32)	1.32 (0.93, 1.71)	1.73 (1.45, 2.00)	1.86 (1.66, 2.07)	1.75 (1.60, 1.91)	1.69 (1.57, 1.82)	1.64 (1.54, 1.74)	1.48 (1.40, 1.57)	1.25 (1.18, 1.32)	1.06 (1.00, 1.12)	0.83 (0.77, 0.89)	0.68 (0.61, 0.74)	0.67 (0.59, 0.74)	0.66 (0.56, 0.75)	0.76 (0.61, 0.90)	0.84 (0.54, 1.13)	1.02 (0.33, 1.71)
		female	-0.75 (-2.36, 0.89)	-0.33 (-1.41, 0.76)	-0.08 (-0.75, 0.60)	0.17 (-0.28, 0.61)	0.28 (-0.03, 0.59)	0.30 (0.06, 0.53)	0.32 (0.14, 0.50)	0.35 (0.21, 0.49)	0.41 (0.29, 0.52)	0.45 (0.36, 0.55)	0.44 (0.36, 0.52)	0.33 (0.25, 0.40)	0.07 (0.00, 0.14)	-0.18 (-0.24, -0.11)	-0.24 (-0.31, -0.17)	-0.19 (-0.27, -0.11)	0.08 (-0.03, 0.19)	0.45 (0.26, 0.64)	0.89 (0.48, 1.30)
High SDI	ASIR (95% UI)	both	0.10 (0.07, 0.15)	0.10 (0.08, 0.14)	0.36 (0.30, 0.42)	0.86 (0.77, 0.96)	1.83 (1.70, 1.97)	3.96 (3.76, 4.17)	8.30 (7.99, 8.62)	16.30 (15.86, 16.76)	30.95 (30.31, 31.61)	57.56 (56.60, 58.53)	93.46 (92.12, 94.81)	140.98 (139.13, 142.86)	198.44 (195.91, 201.01)	262.85 (258.97, 266.79)	336.37 (331.22, 341.60)	394.18 (387.76, 400.70)	449.57 (441.47, 457.83)	403.26 (393.89, 412.85)	488.59 (472.59, 505.14)
		male	0.11 (0.08, 0.16)	0.10 (0.07, 0.13)	0.41 (0.35, 0.48)	0.94 (0.85, 1.04)	1.95 (1.81, 2.10)	4.22 (4.02, 4.44)	8.90 (8.59, 9.23)	17.72 (17.26, 18.19)	34.69 (34.01, 35.39)	67.30 (66.26, 68.36)	115.09 (113.58, 116.63)	180.14 (177.98, 182.32)	258.29 (255.28, 261.35)	341.68 (337.02, 346.40)	429.69 (423.53, 435.93)	492.47 (484.78, 500.28)	546.57 (536.63, 556.69)	461.99 (449.73, 474.59)	556.41 (532.86, 581.01)
		female	0.10 (0.07, 0.14)	0.11 (0.08, 0.15)	0.31 (0.26, 0.37)	0.79 (0.70, 0.89)	1.71 (1.57, 1.86)	3.70 (3.50, 3.92)	7.69 (7.38, 8.02)	14.87 (14.43, 15.33)	27.18 (26.55, 27.83)	47.83 (46.92, 48.76)	72.23 (71.02, 73.46)	103.52 (101.91, 105.16)	143.42 (141.26, 145.62)	194.21 (190.87, 197.61)	259.98 (255.41, 264.63)	318.92 (313.07, 324.87)	379.04 (371.61, 386.62)	355.10 (346.80, 363.58)	433.89 (420.67, 447.53)

	Local Drift (%) (95% CI)	both	-1.05 (-3.70, 1.68)	-0.76 (-2.57, 1.08)	-0.31 (-1.40, 0.79)	0.40 (-0.27, 1.07)	0.89 (0.46, 1.32)	0.96 (0.67, 1.26)	0.78 (0.57, 0.99)	0.54 (0.38, 0.69)	0.27 (0.16, 0.39)	0.11 (0.02, 0.20)	-0.09 (-0.16, -0.01)	-0.30 (-0.37, -0.24)	-0.45 (-0.51, -0.39)	-0.48 (-0.54, -0.43)	-0.40 (-0.46, -0.34)	-0.19 (-0.25, -0.12)	0.07 (-0.02, 0.16)	0.37 (0.21, 0.53)	0.72 (0.38, 1.06)
		male	-1.20 (-3.79, 1.47)	-0.95 (-2.75, 0.89)	-0.44 (-1.49, 0.62)	0.39 (-0.25, 1.04)	0.91 (0.50, 1.33)	0.98 (0.70, 1.26)	0.78 (0.58, 0.98)	0.52 (0.37, 0.66)	0.29 (0.18, 0.40)	0.15 (0.07, 0.24)	-0.04 (-0.10, 0.03)	-0.26 (-0.32, -0.21)	-0.40 (-0.45, -0.34)	-0.41 (-0.46, -0.36)	-0.31 (-0.37, -0.25)	-0.15 (-0.22, -0.08)	-0.01 (-0.12, 0.10)	0.19 (-0.02, 0.40)	0.57 (0.09, 1.05)
		female	-0.88 (-3.75, 2.07)	-0.55 (-2.46, 1.41)	-0.17 (-1.36, 1.04)	0.39 (-0.35, 1.13)	0.84 (0.37, 1.32)	0.93 (0.60, 1.25)	0.76 (0.53, 0.99)	0.54 (0.37, 0.71)	0.23 (0.10, 0.36)	0.03 (-0.07, 0.14)	-0.20 (-0.29, -0.12)	-0.46 (-0.53, -0.39)	-0.69 (-0.75, -0.63)	-0.79 (-0.85, -0.72)	-0.72 (-0.78, -0.66)	-0.43 (-0.50, -0.37)	-0.04 (-0.13, 0.04)	0.38 (0.24, 0.52)	0.75 (0.46, 1.04)
High-middle SDI	ASIR (95% UI)	both	0.05 (0.04, 0.07)	0.06 (0.05, 0.07)	0.31 (0.27, 0.34)	0.65 (0.61, 0.71)	1.31 (1.24, 1.39)	2.89 (2.77, 3.01)	5.72 (5.54, 5.91)	10.85 (10.58, 11.13)	19.76 (19.36, 20.18)	37.46 (36.83, 38.10)	64.10 (63.18, 65.04)	102.53 (101.17, 103.90)	154.07 (152.07, 156.10)	216.86 (213.49, 220.27)	284.31 (279.64, 289.06)	341.72 (335.49, 348.06)	394.59 (385.91, 403.47)	400.44 (387.20, 414.14)	529.83 (499.52, 561.98)
		male	0.04 (0.03, 0.06)	0.04 (0.03, 0.06)	0.28 (0.24, 0.32)	0.59 (0.53, 0.64)	1.18 (1.10, 1.27)	2.74 (2.59, 2.89)	5.75 (5.52, 5.99)	11.27 (10.91, 11.64)	21.42 (20.87, 21.99)	41.81 (40.92, 42.71)	76.48 (75.11, 77.89)	129.45 (127.33, 131.60)	202.79 (199.52, 206.10)	292.79 (287.09, 298.61)	397.03 (388.74, 405.49)	494.08 (482.40, 506.05)	614.27 (596.03, 633.06)	608.91 (578.16, 641.29)	754.92 (681.87, 835.80)
		female	0.08 (0.06, 0.10)	0.09 (0.07, 0.11)	0.36 (0.32, 0.40)	0.79 (0.73, 0.85)	1.55 (1.47, 1.64)	3.16 (3.03, 3.29)	5.78 (5.60, 5.97)	10.50 (10.23, 10.77)	18.09 (17.71, 18.46)	33.12 (32.57, 33.69)	52.18 (51.41, 52.96)	78.01 (76.94, 79.08)	112.56 (111.06, 114.08)	156.44 (153.99, 158.92)	202.48 (199.17, 205.85)	242.72 (238.40, 247.11)	274.83 (269.14, 280.64)	294.79 (286.38, 303.45)	404.13 (385.48, 423.67)
	Local Drift (%) (95% CI)	both	-0.31 (-2.43, 1.86)	0.38 (-1.00, 1.78)	1.05 (0.32, 1.79)	1.66 (1.21, 2.12)	1.99 (1.69, 2.29)	2.02 (1.80, 2.24)	1.81 (1.64, 1.98)	1.62 (1.49, 1.75)	1.52 (1.41, 1.62)	1.38 (1.29, 1.47)	1.26 (1.19, 1.34)	1.18 (1.12, 1.25)	1.12 (1.06, 1.18)	1.15 (1.08, 1.22)	1.24 (1.16, 1.32)	1.28 (1.18, 1.38)	1.41 (1.26, 1.56)	1.51 (1.22, 1.81)	1.86 (1.15, 2.58)
		male	-0.12 (-2.81, 2.64)	0.59 (-1.20, 2.41)	1.41 (0.51, 2.33)	2.26 (1.70, 2.82)	2.81 (2.44, 3.18)	2.96 (2.69, 3.23)	2.68 (2.47, 2.89)	2.43 (2.26, 2.59)	2.26 (2.12, 2.39)	2.00 (1.88, 2.11)	1.73 (1.64, 1.83)	1.58 (1.50, 1.67)	1.46 (1.37, 1.54)	1.49 (1.40, 1.58)	1.63 (1.52, 1.74)	1.72 (1.58, 1.86)	1.92 (1.69, 2.15)	1.97 (1.47, 2.48)	2.11 (0.83, 3.40)
		female	-0.52 (-2.63, 1.64)	0.11 (-1.24, 1.47)	0.51 (-0.25, 1.27)	0.78 (0.32, 1.25)	0.80 (0.49, 1.12)	0.68 (0.45, 0.90)	0.59 (0.42, 0.76)	0.51 (0.38, 0.64)	0.52 (0.42, 0.63)	0.55 (0.46, 0.63)	0.61 (0.53, 0.68)	0.60 (0.53, 0.66)	0.56 (0.50, 0.62)	0.57 (0.51, 0.64)	0.67 (0.60, 0.74)	0.73 (0.65, 0.82)	0.93 (0.81, 1.06)	1.21 (0.97, 1.44)	1.73 (1.18, 2.29)
Middle SDI	ASIR (95% UI)	both	0.04 (0.03, 0.06)	0.05 (0.04, 0.06)	0.22 (0.19, 0.24)	0.45 (0.41, 0.49)	0.87 (0.81, 0.94)	1.87 (1.77, 1.97)	3.47 (3.32, 3.63)	6.22 (6.00, 6.46)	11.28 (10.93, 11.63)	20.88 (20.33, 21.44)	36.40 (35.56, 37.25)	60.17 (58.91, 61.47)	96.44 (94.44, 98.48)	153.19 (149.23, 157.27)	218.75 (212.55, 225.14)	282.63 (273.40, 292.17)	360.44 (345.48, 376.05)	347.78 (323.66, 373.71)	418.48 (366.39, 477.98)
		male	0.03 (0.03, 0.04)	0.03 (0.03, 0.04)	0.19 (0.17, 0.21)	0.38 (0.35, 0.42)	0.77 (0.72, 0.83)	1.76 (1.67, 1.86)	3.53 (3.38, 3.68)	6.57 (6.34, 6.81)	12.49 (12.12, 12.87)	23.25 (22.67, 23.85)	43.23 (42.28, 44.20)	73.98 (72.51, 75.48)	122.86 (120.43, 125.33)	197.13 (192.19, 202.19)	290.73 (282.67, 299.02)	376.30 (363.98, 389.03)	507.07 (485.20, 529.94)	447.46 (410.64, 487.60)	501.87 (424.24, 593.70)

		female	0.07 (0.05, 0.09)	0.08 (0.06, 0.11)	0.27 (0.23, 0.32)	0.57 (0.51, 0.64)	1.07 (0.97, 1.17)	2.08 (1.94, 2.23)	3.47 (3.27, 3.68)	5.87 (5.59, 6.16)	9.94 (9.54, 10.36)	18.40 (17.77, 19.05)	29.36 (28.46, 30.29)	46.55 (45.24, 47.91)	71.67 (69.69, 73.72)	113.69 (109.84, 117.68)	158.32 (152.52, 164.34)	207.77 (199.24, 216.67)	257.43 (244.49, 271.06)	267.21 (246.89, 289.20)	331.19 (287.90, 380.98)
	Local Drift (%) (95% CI)	both	0.09 (-1.85, 2.07)	0.75 (-0.53, 2.06)	1.20 (0.46, 1.94)	1.32 (0.83, 1.81)	1.42 (1.07, 1.78)	1.73 (1.46, 2.00)	2.02 (1.80, 2.24)	2.41 (2.22, 2.59)	2.72 (2.56, 2.87)	2.86 (2.72, 3.00)	2.84 (2.72, 2.97)	3.02 (2.90, 3.14)	3.06 (2.94, 3.18)	2.96 (2.83, 3.09)	2.88 (2.72, 3.04)	2.73 (2.51, 2.95)	2.38 (2.04, 2.72)	1.92 (1.24, 2.61)	1.95 (0.28, 3.64)
		male	0.41 (-1.49, 2.34)	1.23 (-0.06, 2.52)	1.85 (1.15, 2.56)	2.11 (1.64, 2.59)	2.32 (1.98, 2.66)	2.65 (2.39, 2.92)	2.90 (2.69, 3.12)	3.30 (3.13, 3.48)	3.60 (3.44, 3.75)	3.68 (3.55, 3.82)	3.55 (3.42, 3.67)	3.67 (3.55, 3.79)	3.67 (3.55, 3.79)	3.56 (3.42, 3.69)	3.47 (3.30, 3.64)	3.36 (3.11, 3.61)	3.02 (2.63, 3.42)	2.43 (1.56, 3.30)	2.24 (0.00, 4.53)
		female	-0.33 (-2.83, 2.23)	0.13 (-1.50, 1.78)	0.28 (-0.70, 1.27)	0.23 (-0.42, 0.89)	0.19 (-0.28, 0.65)	0.42 (0.06, 0.78)	0.76 (0.47, 1.05)	1.14 (0.90, 1.38)	1.50 (1.30, 1.71)	1.75 (1.57, 1.93)	1.90 (1.74, 2.07)	2.16 (2.01, 2.32)	2.26 (2.11, 2.42)	2.22 (2.06, 2.38)	2.20 (2.01, 2.39)	2.11 (1.87, 2.36)	1.86 (1.49, 2.23)	1.60 (0.89, 2.31)	1.81 (0.12, 3.52)
Low-middle SDI	ASIR (95% UI)	both	0.05 (0.05, 0.06)	0.05 (0.04, 0.05)	0.17 (0.16, 0.18)	0.34 (0.32, 0.36)	0.57 (0.54, 0.59)	1.18 (1.14, 1.22)	2.10 (2.05, 2.16)	3.60 (3.53, 3.68)	7.21 (7.08, 7.34)	12.89 (12.69, 13.09)	20.61 (20.33, 20.90)	31.98 (31.57, 32.39)	47.05 (46.47, 47.64)	67.95 (66.92, 69.00)	93.42 (91.86, 94.99)	123.94 (121.57, 126.35)	145.43 (141.80, 149.15)	145.99 (139.96, 152.28)	170.85 (157.79, 184.99)
		male	0.05 (0.04, 0.05)	0.04 (0.04, 0.05)	0.16 (0.15, 0.17)	0.29 (0.27, 0.31)	0.51 (0.49, 0.53)	1.06 (1.02, 1.10)	2.01 (1.95, 2.07)	3.37 (3.29, 3.46)	6.84 (6.69, 6.98)	12.51 (12.28, 12.74)	21.86 (21.50, 22.22)	36.38 (35.85, 36.93)	54.76 (53.97, 55.57)	77.99 (76.58, 79.41)	104.94 (102.85, 107.07)	135.62 (132.46, 138.86)	161.80 (156.74, 167.02)	152.80 (144.27, 161.83)	176.78 (157.30, 198.67)
		female	0.06 (0.05, 0.07)	0.06 (0.05, 0.07)	0.17 (0.15, 0.19)	0.40 (0.38, 0.43)	0.63 (0.60, 0.67)	1.32 (1.26, 1.38)	2.20 (2.12, 2.28)	3.85 (3.74, 3.98)	7.61 (7.42, 7.81)	13.28 (12.99, 13.58)	19.33 (18.94, 19.73)	27.70 (27.17, 28.24)	39.87 (39.12, 40.63)	58.93 (57.59, 60.29)	83.25 (81.21, 85.34)	113.41 (110.27, 116.63)	131.38 (126.75, 136.17)	137.50 (129.96, 145.47)	160.79 (145.33, 177.89)
	Local Drift (%) (95% CI)	both	-0.75 (-1.49, -0.01)	-0.07 (-0.58, 0.45)	0.26 (-0.08, 0.60)	0.47 (0.23, 0.72)	0.57 (0.38, 0.76)	0.89 (0.74, 1.04)	1.22 (1.09, 1.34)	1.58 (1.48, 1.69)	1.77 (1.68, 1.85)	1.86 (1.79, 1.94)	1.75 (1.68, 1.82)	1.75 (1.68, 1.81)	1.71 (1.65, 1.78)	1.73 (1.66, 1.81)	1.79 (1.70, 1.88)	1.95 (1.83, 2.08)	1.97 (1.77, 2.18)	1.90 (1.48, 2.31)	1.98 (0.95, 3.02)
		male	-0.81 (-1.71, 0.09)	-0.04 (-0.66, 0.59)	0.41 (0.01, 0.81)	0.69 (0.40, 0.99)	0.90 (0.67, 1.13)	1.31 (1.13, 1.50)	1.68 (1.53, 1.83)	2.11 (1.99, 2.24)	2.32 (2.21, 2.42)	2.33 (2.24, 2.42)	2.13 (2.05, 2.22)	2.08 (2.00, 2.16)	2.00 (1.92, 2.08)	2.02 (1.93, 2.11)	2.08 (1.97, 2.19)	2.22 (2.07, 2.38)	2.22 (1.96, 2.49)	2.08 (1.50, 2.66)	2.13 (0.59, 3.70)
		female	-0.69 (-1.77, 0.40)	-0.10 (-0.86, 0.66)	0.10 (-0.40, 0.61)	0.23 (-0.13, 0.59)	0.23 (-0.05, 0.51)	0.45 (0.23, 0.67)	0.74 (0.55, 0.92)	1.03 (0.88, 1.18)	1.20 (1.07, 1.32)	1.38 (1.27, 1.49)	1.35 (1.24, 1.45)	1.39 (1.30, 1.49)	1.41 (1.31, 1.51)	1.45 (1.34, 1.55)	1.52 (1.39, 1.64)	1.72 (1.55, 1.89)	1.78 (1.50, 2.05)	1.77 (1.23, 2.31)	1.89 (0.61, 3.19)
Low SDI	ASIR (95% UI)	both	0.05 (0.04, 0.05)	0.04 (0.04, 0.05)	0.11 (0.10, 0.13)	0.26 (0.24, 0.28)	0.45 (0.42, 0.48)	0.92 (0.88, 0.97)	1.69 (1.62, 1.76)	2.84 (2.74, 2.94)	5.71 (5.56, 5.88)	9.90 (9.67, 10.15)	14.97 (14.63, 15.30)	22.69 (22.22, 23.17)	32.25 (31.58, 32.93)	44.24 (43.13, 45.37)	59.45 (57.83, 61.11)	78.00 (75.55, 80.52)	86.50 (82.90, 90.26)	87.08 (81.11, 93.49)	88.39 (76.01, 102.78)

	male	0.05 (0.04, 0.06)	0.04 (0.03, 0.05)	0.12 (0.11, 0.14)	0.25 (0.22, 0.28)	0.41 (0.38, 0.45)	0.87 (0.81, 0.94)	1.64 (1.55, 1.74)	2.82 (2.69, 2.96)	5.50 (5.29, 5.72)	9.69 (9.36, 10.02)	15.74 (15.26, 16.22)	25.67 (24.96, 26.41)	37.00 (35.98, 38.04)	49.22 (47.58, 50.92)	64.23 (61.87, 66.69)	79.56 (76.13, 83.15)	91.85 (86.51, 97.52)	87.81 (78.78, 97.89)	92.09 (71.46, 118.67)
	female	0.04 (0.04, 0.05)	0.05 (0.04, 0.06)	0.10 (0.09, 0.12)	0.26 (0.23, 0.29)	0.49 (0.45, 0.53)	0.97 (0.90, 1.04)	1.73 (1.63, 1.83)	2.85 (2.72, 2.99)	5.94 (5.71, 6.18)	10.14 (9.79, 10.50)	14.20 (13.74, 14.68)	19.71 (19.09, 20.34)	27.54 (26.69, 28.42)	39.59 (38.12, 41.11)	55.53 (53.30, 57.85)	77.90 (74.38, 81.59)	83.93 (78.99, 89.18)	88.99 (80.95, 97.83)	89.64 (74.23, 108.25)
Local Drift (%) (95% CI)	both	-0.76 (-1.66, 0.14)	-0.37 (-1.05, 0.31)	-0.03 (-0.53, 0.46)	0.23 (-0.15, 0.60)	0.38 (0.09, 0.68)	0.49 (0.26, 0.73)	0.55 (0.36, 0.74)	0.61 (0.44, 0.77)	0.59 (0.46, 0.73)	0.61 (0.49, 0.73)	0.61 (0.50, 0.72)	0.65 (0.55, 0.76)	0.71 (0.60, 0.81)	0.80 (0.69, 0.91)	0.90 (0.76, 1.04)	1.07 (0.88, 1.26)	1.21 (0.89, 1.53)	1.41 (0.75, 2.08)	1.69 (-0.02, 3.42)
	male	-0.91 (-2.12, 0.31)	-0.42 (-1.34, 0.51)	0.10 (-0.56, 0.78)	0.53 (0.01, 1.05)	0.75 (0.33, 1.17)	0.80 (0.47, 1.14)	0.75 (0.47, 1.02)	0.70 (0.47, 0.94)	0.62 (0.43, 0.82)	0.59 (0.42, 0.76)	0.57 (0.42, 0.72)	0.59 (0.45, 0.73)	0.63 (0.49, 0.77)	0.70 (0.55, 0.86)	0.79 (0.60, 0.99)	0.94 (0.67, 1.22)	1.09 (0.62, 1.56)	1.31 (0.28, 2.34)	1.60 (-1.25, 4.53)
	female	-0.58 (-1.91, 0.77)	-0.30 (-1.29, 0.71)	-0.16 (-0.90, 0.58)	-0.07 (-0.61, 0.47)	0.03 (-0.39, 0.44)	0.20 (-0.13, 0.53)	0.36 (0.09, 0.63)	0.51 (0.28, 0.74)	0.57 (0.38, 0.77)	0.65 (0.48, 0.82)	0.68 (0.52, 0.84)	0.78 (0.62, 0.93)	0.86 (0.71, 1.02)	0.97 (0.80, 1.14)	1.06 (0.86, 1.26)	1.21 (0.94, 1.49)	1.33 (0.88, 1.78)	1.50 (0.63, 2.38)	1.75 (-0.37, 3.92)

Notes:

Age-standardized incidence rate is computed by direct standardization with global standard population in GBD 2019.

Local drifts are estimates derived from the age-period-cohort model for 19 age groups (5−9 to 95 plus years), 1990−2019.

Parenthesis for all GBD health estimate indicates 95% uncertainty intervals; parenthesis for local drift indicates 95% confidence intervals.

Figure S1. The age–standardized incidence (ASIR) heatmap of cancers by SDI quintiles, sexes, ages. (A) The ASIR heatmap of cancers by SDI quintiles and sexes in 2019. (B) The incidence percent heatmap of cancers by ages worldwide in 2019.

A

	Both						Female						Male					
	Global	High SDI	High-middle SDI	Middle SDI	Low-middle SDI	Low SDI	Global	High SDI	High-middle SDI	Middle SDI	Low-middle SDI	Low SDI	Global	High SDI	High-middle SDI	Middle SDI	Low-middle SDI	Low SDI
Tracheal, bronchus, and lung cancer	27.66	37.36	32.65	27	12.68	8.82	16.84	27.33	17.13	15.89	7.39	4.21	40.44	49.65	52.04	39.54	18.51	13.67
Colon and rectum cancer	26.71	42.78	32.45	20.94	11.35	7.74	21.22	34.08	24.58	16.19	10.4	7.27	33.06	52.8	42.24	26.15	12.38	8.25
Breast cancer	24.17	41.22	26	18.52	15.38	13.51	45.86	79.22	48.93	35.52	29.47	25.67	0.65	0.71	0.67	0.6	0.55	1
Prostate cancer	17.39	36.63	15.42	10.76	8.54	9.59							38.63	79.7	35.74	23.64	18.53	20.25
Stomach cancer	15.59	12.4	18.77	18.68	10.96	8.38	9.71	7.91	10.45	10.98	8.94	6.97	22.39	17.78	28.91	27.24	13.19	9.89
Leukemia	8.22	11.99	10.11	6.9	4.72	5.1	7.23	9.96	9.01	6.25	4.43	4.64	9.42	14.43	11.5	7.62	5.06	5.59
Cervical cancer	6.81	4.48	5.93	6.87	8.05	11.73	13.35	8.91	11.59	13.44	15.78	23.21						
Pancreatic cancer	6.57	10.16	7.67	4.8	3.54	2.54	5.71	8.82	6.22	4.16	3.36	2.33	7.49	11.6	9.36	5.49	3.73	2.76
Bladder cancer	6.52	9.92	8.75	4.28	2.68	3.27	2.66	4.48	2.92	1.63	1.42	1.88	11.28	16.62	16.47	7.31	4.1	4.77
Liver cancer	6.51	7.61	5.34	8.28	4.05	3.69	3.63	4.06	2.75	4.43	2.85	2.65	9.71	11.61	8.29	12.41	5.35	4.79
Esophageal cancer	6.51	5.2	7.06	8.42	4.37	5.39	3.33	1.93	3.06	4.58	3.01	4.48	10.13	8.92	11.86	12.7	5.84	6.3
Non−Hodgkin lymphoma	5.73	9.93	5.86	4.13	3.05	2.36	4.45	7.72	4.42	3.09	2.43	2.17	7.2	12.54	7.54	5.23	3.69	2.55
Uterine cancer	5.21	9.91	7.32	2.93	2.03	1.74	9.99	19.16	13.87	5.7	3.94	3.43						
Kidney cancer	4.55	9.02	6.09	2.63	1.54	1.22	3.07	5.88	4.12	1.85	1.14	0.95	6.24	12.54	8.41	3.5	1.98	1.5
Lip and oral cavity cancer	4.52	4.71	3.56	3.72	6.65	5.36	3.01	2.68	1.99	2.39	5.3	4.53	6.16	6.89	5.36	5.15	8.1	6.2
Brain and central nervous system cancer	4.34	6.46	5.64	4.01	2.63	2.01	3.89	5.57	5.24	3.81	2.27	1.59	4.84	7.4	6.11	4.23	3	2.42
Ovarian cancer	3.58	4.83	3.96	2.93	2.9	2.61	6.87	9.3	7.56	5.71	5.65	5.15						
Malignant skin melanoma	3.56	12.4	3.54	0.73	0.43	0.54	3.2	11.27	3.6	0.72	0.42	0.55	4.05	13.95	3.54	0.75	0.43	0.53
Thyroid cancer	2.83	4.59	3.06	2.49	1.91	1.63	3.74	5.68	4.07	3.3	2.7	2.43	1.9	3.5	2.02	1.67	1.1	0.82
Larynx cancer	2.51	2.48	2.8	2.23	2.69	2.29	0.65	0.68	0.54	0.63	0.81	0.76	4.58	4.49	5.44	3.99	4.73	3.88
Gallbladder and biliary tract cancer	2.49	3.15	2.5	1.92	2.23	1.86	2.58	2.91	2.5	2.01	2.71	2.34	2.39	3.43	2.52	1.81	1.71	1.36
Nasopharynx cancer	2.12	1.29	3.58	2.5	1.1	0.93	1.16	0.71	1.71	1.45	0.73	0.63	3.14	1.9	5.56	3.6	1.48	1.23
Other pharynx cancer	1.99	2.75	1.57	1.2	3.35	1.81	0.87	0.96	0.48	0.55	1.93	0.92	3.23	4.67	2.78	1.9	4.88	2.73
Multiple myeloma	1.92	3.77	1.83	1.06	1	1.07	1.62	3.07	1.55	0.91	0.92	1.03	2.28	4.61	2.21	1.24	1.09	1.11
Testicular cancer	1.4	3.97	2.71	1	0.44	0.29							2.79	7.75	5.31	1.99	0.88	0.59
Hodgkin lymphoma	1.1	2.51	1.36	0.64	0.6	0.75	0.89	2.22	1.24	0.51	0.42	0.49	1.34	2.86	1.5	0.78	0.78	1.01
Mesothelioma	0.43	0.91	0.36	0.2	0.25	0.22	0.22	0.31	0.23	0.13	0.18	0.22	0.69	1.64	0.52	0.27	0.33	0.22

Rank: 5, 10, 15, 20, 25

B

	All ages	5 to 9	10 to 14	15 to 19	20 to 24	25 to 29	30 to 34	35 to 39	40 to 44	45 to 49	50 to 54	55 to 59	60 to 64	65 to 69	70 to 74	75 to 79	80 to 84	85 to 89	90 to 94	95 plus
Tracheal, bronchus, and lung cancer	13.79		0.62	1.83	2.07	2.37	3.05	3.88	5.28	8.05	11.05	13.6	15.49	16.76	17.58	17.04	16.48	15.62	13.27	10.5
Colon and rectum cancer	13.22	0.82	1.22	3.39	4.93	6.24	7.72	8.47	9.27	10.39	11.71	12.34	12.96	13.71	14.5	15.68	16.56	17.39	17.77	21.88
Breast cancer	12.22			4.27	6.2	11.5	17.15	21.68	25.47	23.74	18.91	14.96	12.22	10.22	8.14	7.34	7.61	7.2	7.48	11.48
Prostate cancer	8.61				0.77	0.71	0.52	0.46	0.6	1.5	3.35	6.29	9.77	11.78	12.76	13.07	11.79	10.59	10.08	8.65
Stomach cancer	7.75			1.94	3.18	3.99	5.27	5.3	6.13	6.46	7.08	7.32	7.67	8.25	8.76	8.77	9.39	9.17	9.2	7.83
Leukemia	3.93	48.37	39.28	26.64	17.81	11.32	7.07	5.87	3.84	3.43	3.01	2.73	2.6	2.52	2.68	2.86	2.89	3.29	3.96	6.04
Cervical cancer	3.45			2	5.45	9.91	13.6	13.29	11.18	7.74	5.54	3.91	2.61	1.88	1.43	1.18	1.09	0.92	0.89	0.9
Esophageal cancer	3.26				0.53	0.57	0.72	1.06	1.65	2.53	3.28	3.62	3.68	3.83	3.93	3.65	3.62	3.23	2.58	1.69
Liver cancer	3.26	1.6	3.33	1.76	1.75	2.05	2.5	2.78	3.3	3.66	3.79	3.62	3.42	3.25	3.16	3.2	3.23	3.18	2.5	2.09
Pancreatic cancer	3.24			0.35	0.45	0.6	0.91	1.2	1.53	2.02	2.46	2.9	3.23	3.48	3.75	3.97	4.66	4.76	5.83	4.66
Bladder cancer	3.2			1.08	0.96	0.98	1.34	1.62	1.46	1.77	2.26	2.57	3	3.27	3.69	4.17	4.57	5.82	5.62	5.38
Non−Hodgkin lymphoma	2.79	13.29	15.16	15.32	7.93	5.46	3.85	3.15	2	1.89	1.96	2.05	2.06	2.31	2.76	3.27	3.1	3.92	5.03	5.38
Uterine cancer	2.66				1.07	1.6	1.99	2.33	2.47	3.25	4.08	4.05	3.59	2.84	2.25	1.82	1.52	1.27	1.21	1.17
Lip and oral cavity cancer	2.28	0.77	1.37	2.39	2.72	2.64	2.65	3.04	2.93	3.02	2.99	2.91	2.48	2.19	1.91	1.85	1.55	1.49	1.58	1.18
Kidney cancer	2.27	5.23	2.85	1.96	1.85	1.84	1.92	2.18	2.44	2.45	2.4	2.43	2.37	2.25	2.18	2.13	2.03	1.78	1.8	1.45
Brain and central nervous system cancer	2.12	20.35	21.09	11.3	8.3	7.23	5.43	4.06	3.03	2.53	2.1	1.88	1.64	1.5	1.36	1.26	1.13	1.11	1.65	0.49
Ovarian cancer	1.8	1.67	3.34	4.73	4.81	4.08	2.9	2.91	2.89	2.92	2.48	2.05	1.73	1.49	1.29	1.14	1.25	1.06	1.29	1.22
Malignant skin melanoma	1.77	0.65	0.89	2.29	3.46	4.43	3.81	3.21	2.72	2.13	1.82	1.7	1.48	1.47	1.49	1.48	1.59	1.8	1.86	2.24
Thyroid cancer	1.43	1.93	2.65	3.78	4.81	5.07	4.74	4.01	3.41	2.61	2.1	1.64	1.2	0.92	0.74	0.68	0.54	0.51	0.39	0.77
Larynx cancer	1.28				0.34	0.29	0.34	0.56	0.88	1.33	1.62	1.89	1.83	1.55	1.26	1.03	0.82	0.7	0.53	0.33
Gallbladder and biliary tract cancer	1.22				0.25	0.28	0.37	0.48	0.6	0.69	0.84	0.98	1.09	1.19	1.34	1.58	1.86	2.34	2.65	2.6
Nasopharynx cancer	1.08	0.7	1.54	2.11	1.92	2.19	3.25	2.85	2.8	2.48	1.89	1.36	0.92	0.71	0.49	0.37	0.32	0.41	0.28	0.17
Other pharynx cancer	1.02				0.76	0.6	0.6	0.85	1.18	1.42	1.61	1.59	1.39	1.04	0.86	0.64	0.53	0.4	0.32	0.25
Multiple myeloma	0.95				0.35	0.25	0.26	0.32	0.43	0.58	0.75	0.89	0.98	1.04	1.14	1.2	1.26	1.31	1.36	1.08
Testicular cancer	0.67	1.48	1.82	5.56	9.95	8.99	5.44	2.89	1.47	0.7	0.35	0.21	0.15	0.1	0.07	0.08	0.06	0.07	0.04	0.03
Hodgkin lymphoma	0.53	3.13	4.83	7.3	7.2	4.62	2.47	1.4	0.87	0.55	0.41	0.31	0.26	0.24	0.23	0.26	0.26	0.39	0.6	0.42
Mesothelioma	0.21				0.18	0.18	0.12	0.15	0.15	0.15	0.17	0.2	0.17	0.2	0.25	0.29	0.29	0.28	0.25	0.13

Figure S2-A. The local drifts of colorectal cancer incidence in 21 GBD regions, 1990-2019.

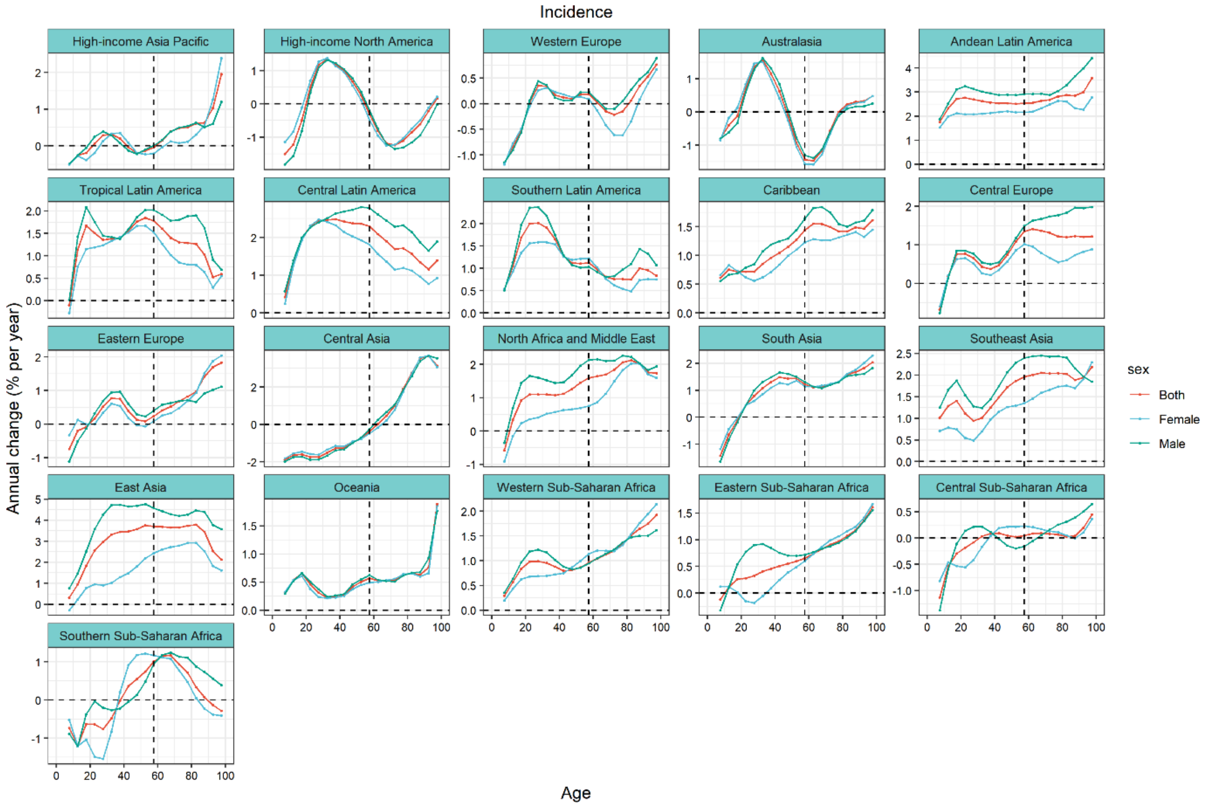

Local drifts of colorectal cancer incidence (estimates from age-period-cohort models) for 19 age groups (5−9 to 95 plus years), 1990−2019. The dots and shaded areas indicate the annual percentage change of incidence (% per year) and the corresponding 95% CIs.

Figure S2-B. Age distribution of absolute cases of colorectal cancer incidence in 21 GBD regions, 1990-2019

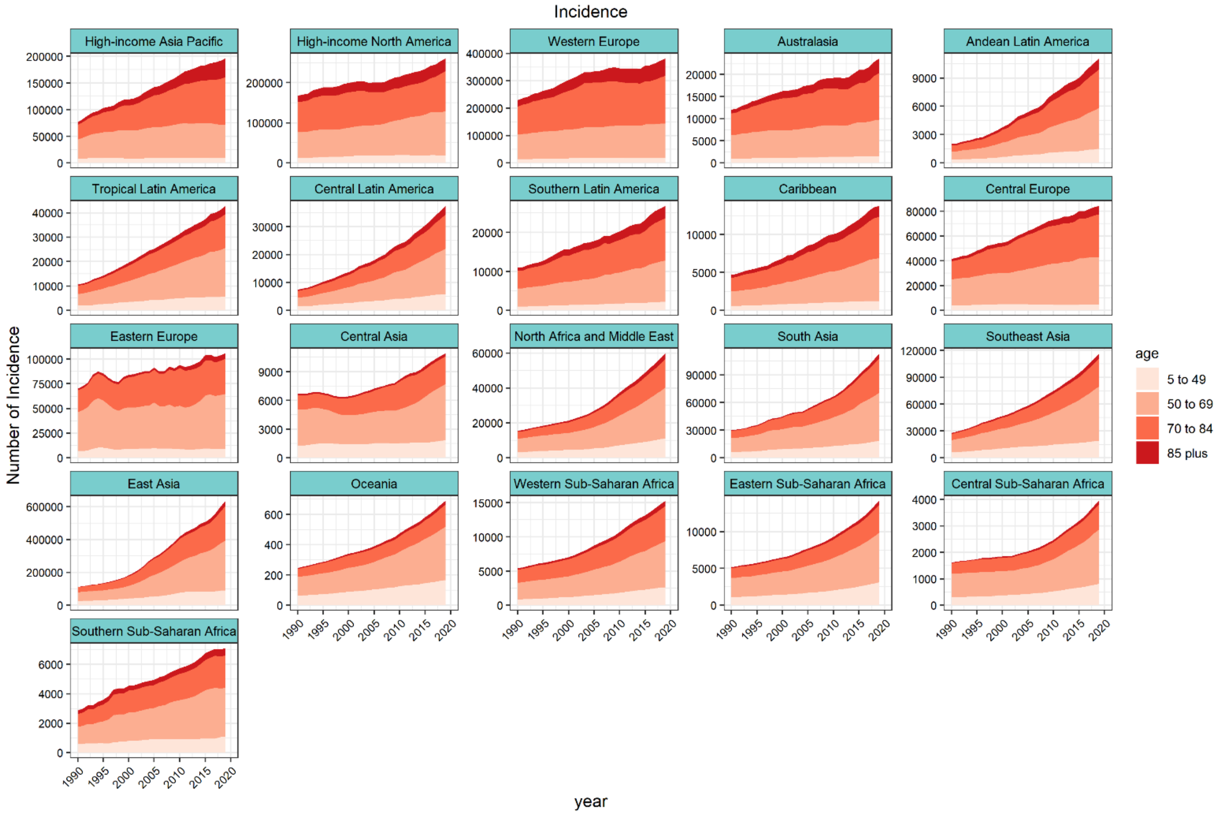

Figure S2-C. Age distribution of relative proportion of colorectal cancer incidence in 21 GBD regions, 1990-2019

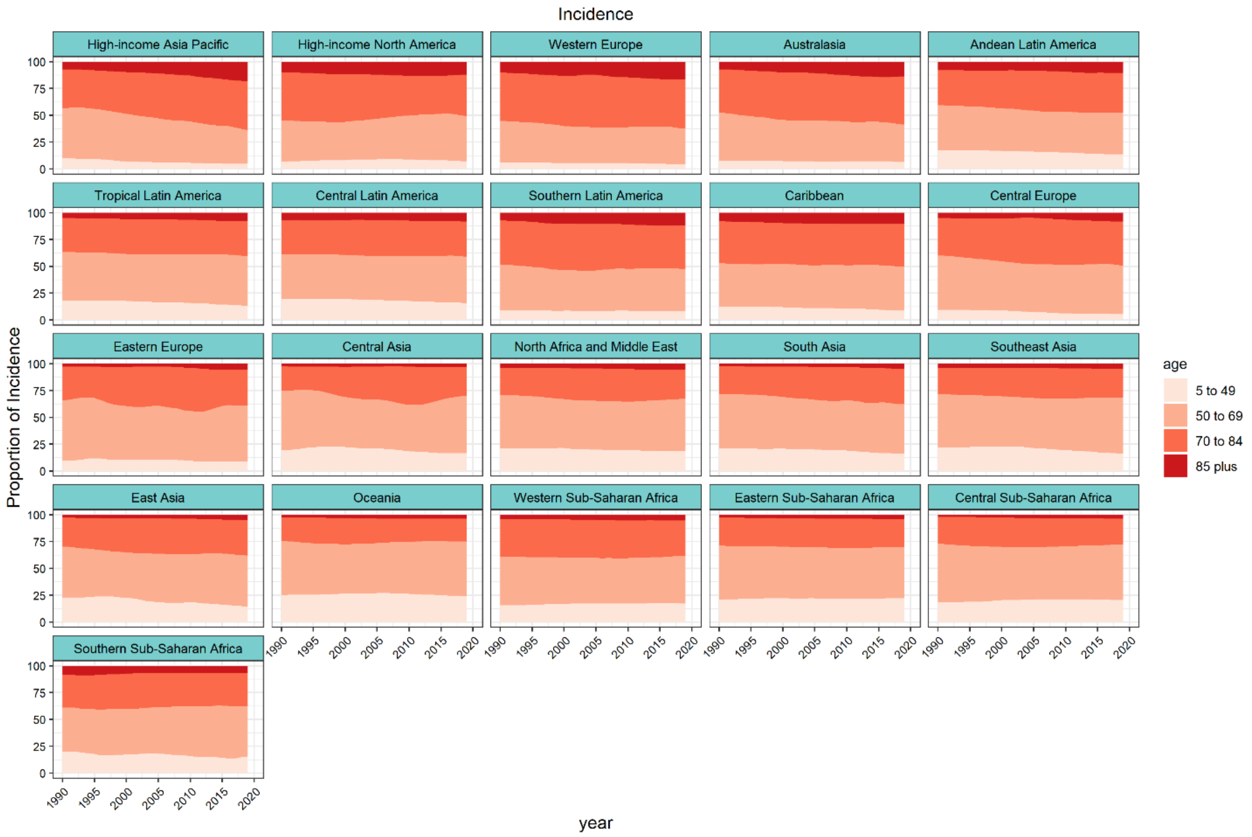

Figure S3-A. The local drifts of colorectal cancer incidence in high-SDI countries, 1990-2019.

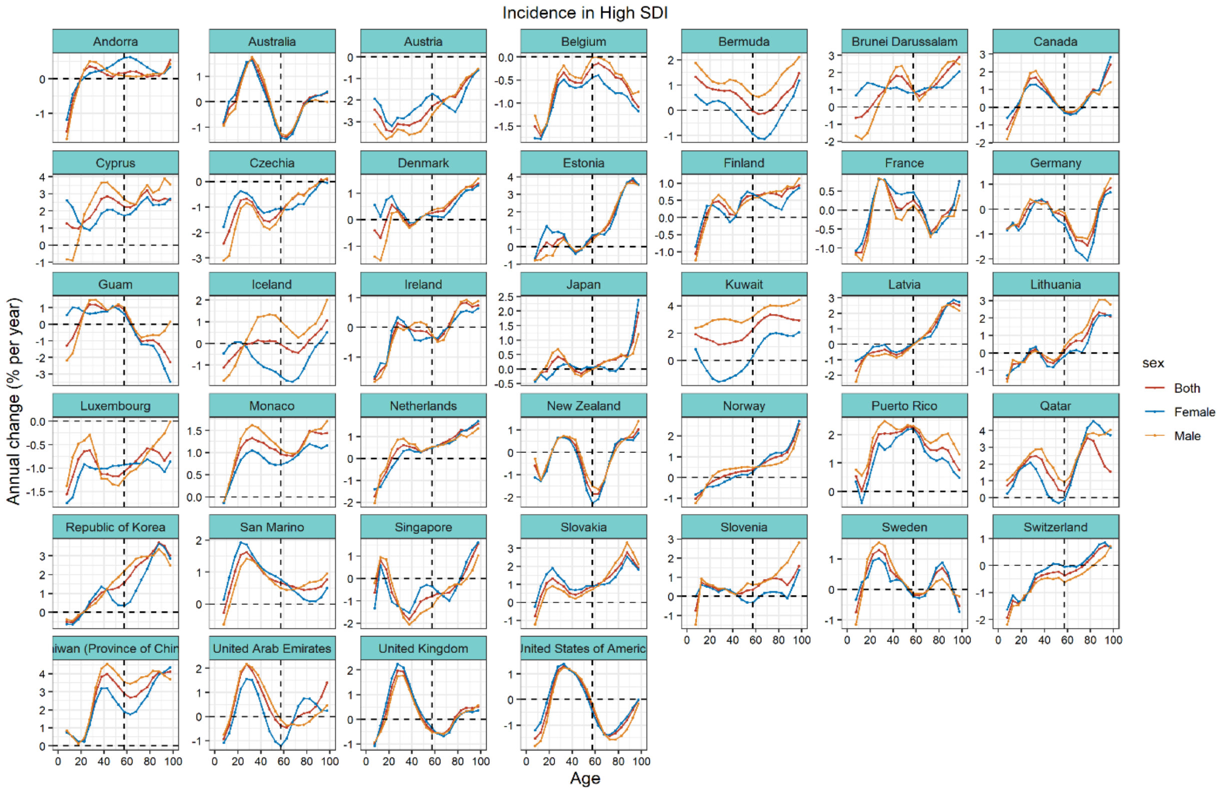

Local drifts of colorectal cancer incidence (estimates from age-period-cohort models) for 19 age groups (5−9 to 95 plus years), 1990−2019. The dots and shaded areas indicate the annual percentage change of incidence (% per year) and the corresponding 95% CIs.

Figure S3-B. Age distribution of absolute cases of colorectal cancer incidence in high-SDI countries, 1990-2019

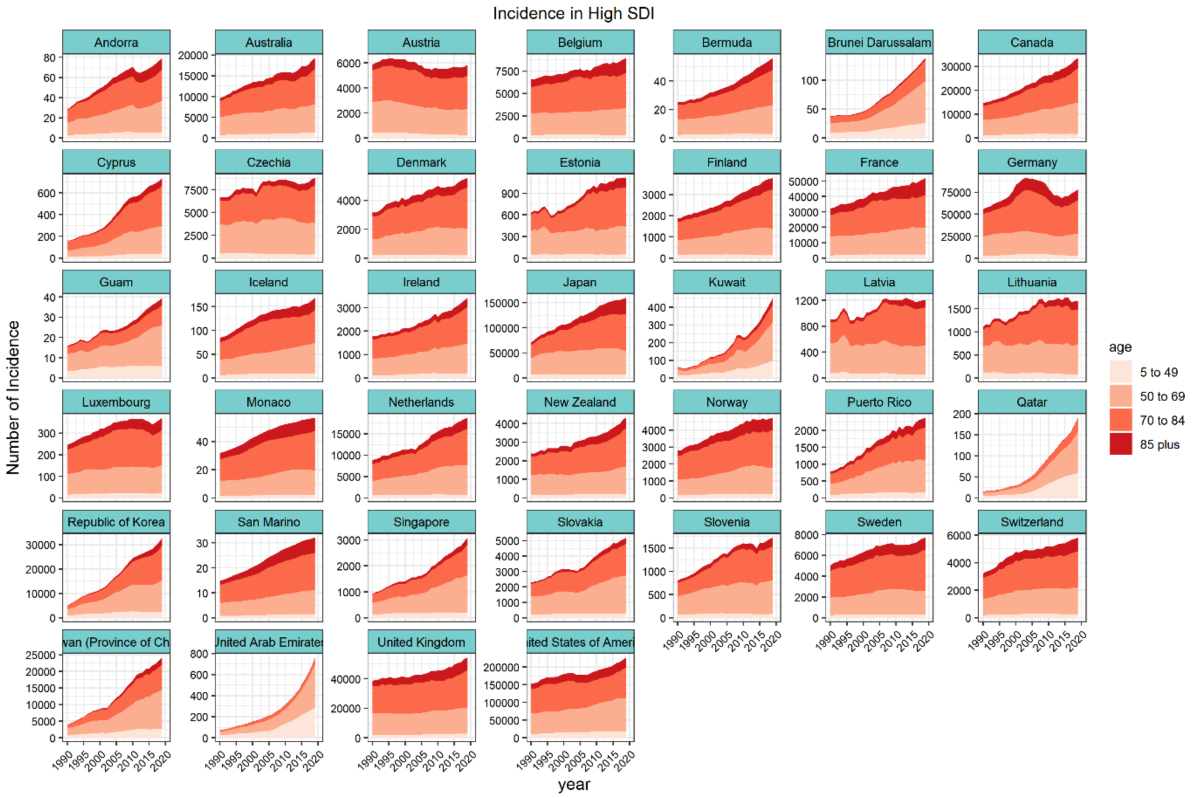

Figure S3-C. Age distribution of relative proportion of colorectal cancer incidence in high-SDI countries, 1990-2019

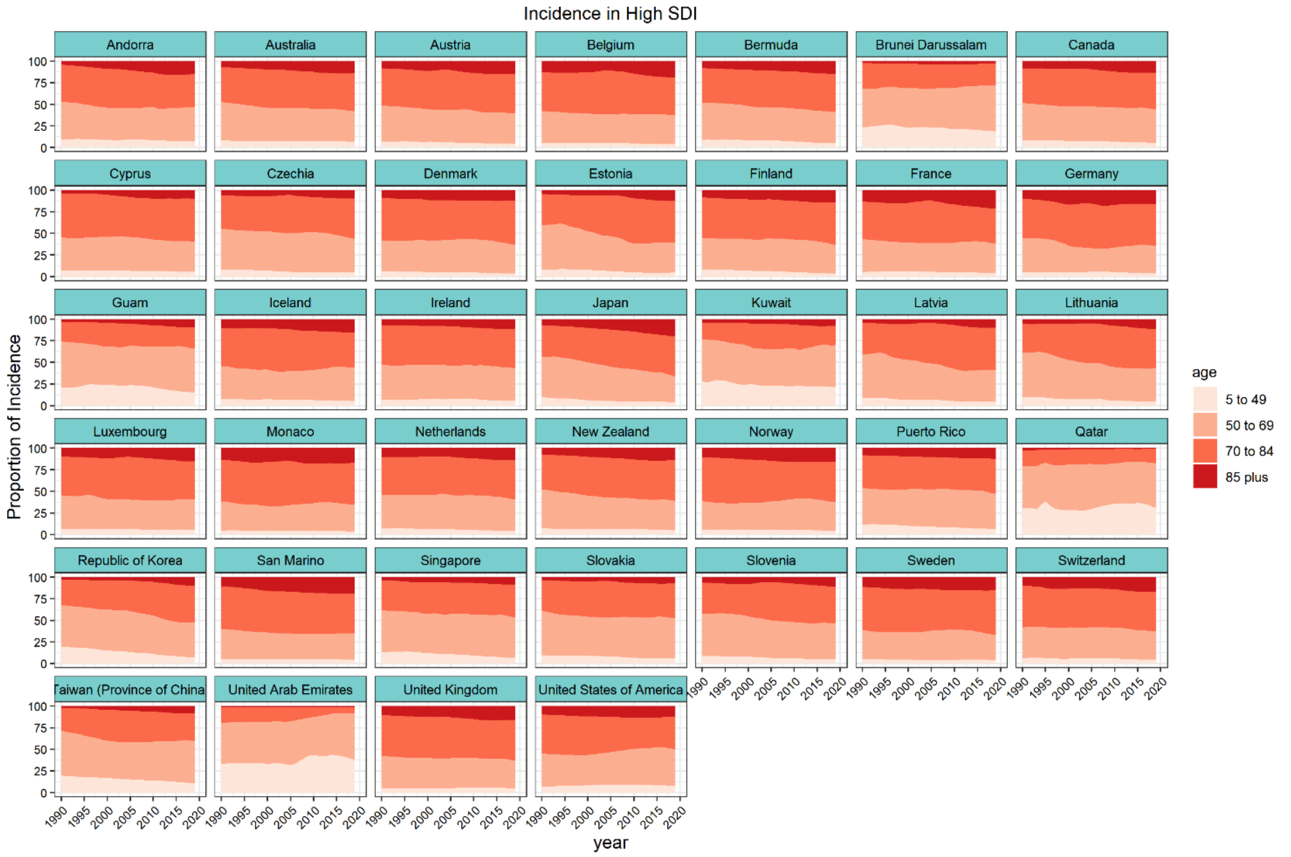

Figure S4-A. The local drifts of colorectal cancer incidence in high-middle-SDI countries, 1990-2019.

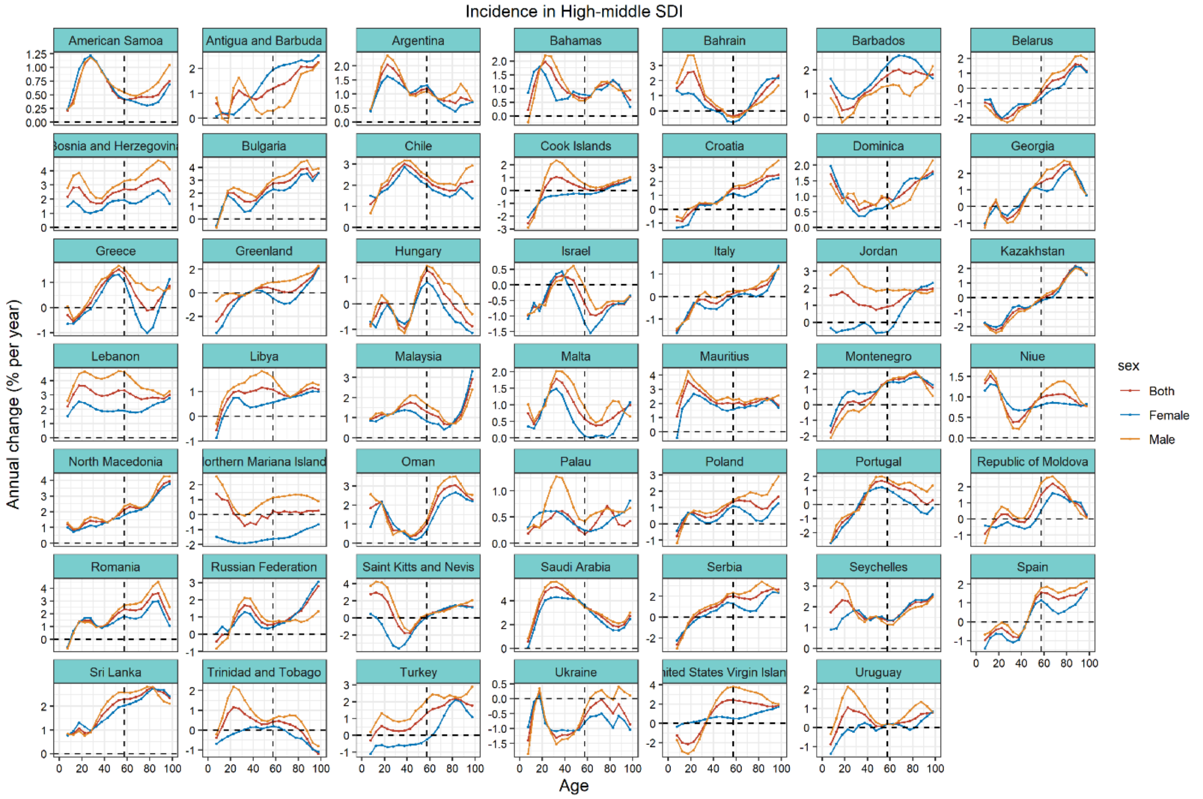

Local drifts of colorectal cancer incidence (estimates from age-period-cohort models) for 19 age groups (5−9 to 95 plus years), 1990−2019. The dots and shaded areas indicate the annual percentage change of incidence (% per year) and the corresponding 95% CIs.

Figure S4-B. Age distribution of absolute cases of colorectal cancer incidence in high-middle-SDI countries, 1990-2019

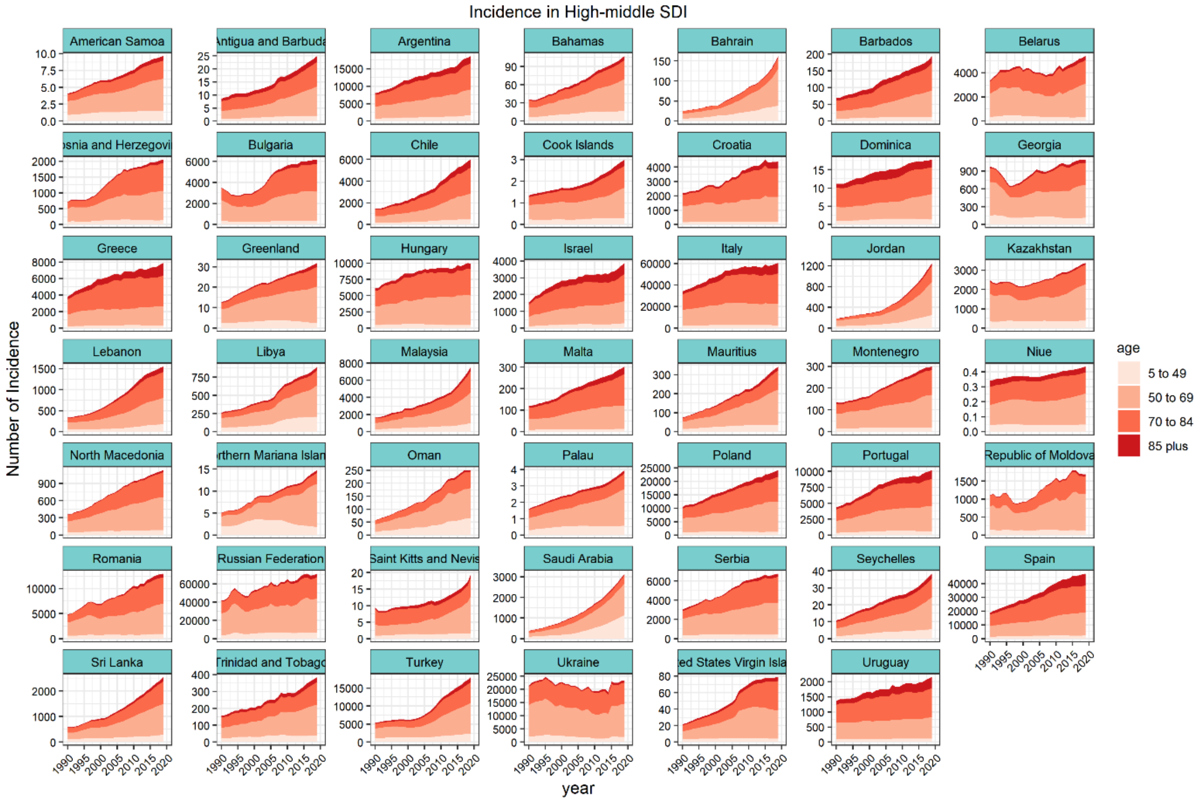

Figure S4-C. Age distribution of relative proportion of colorectal cancer incidence in high-middle-SDI countries, 1990-2019

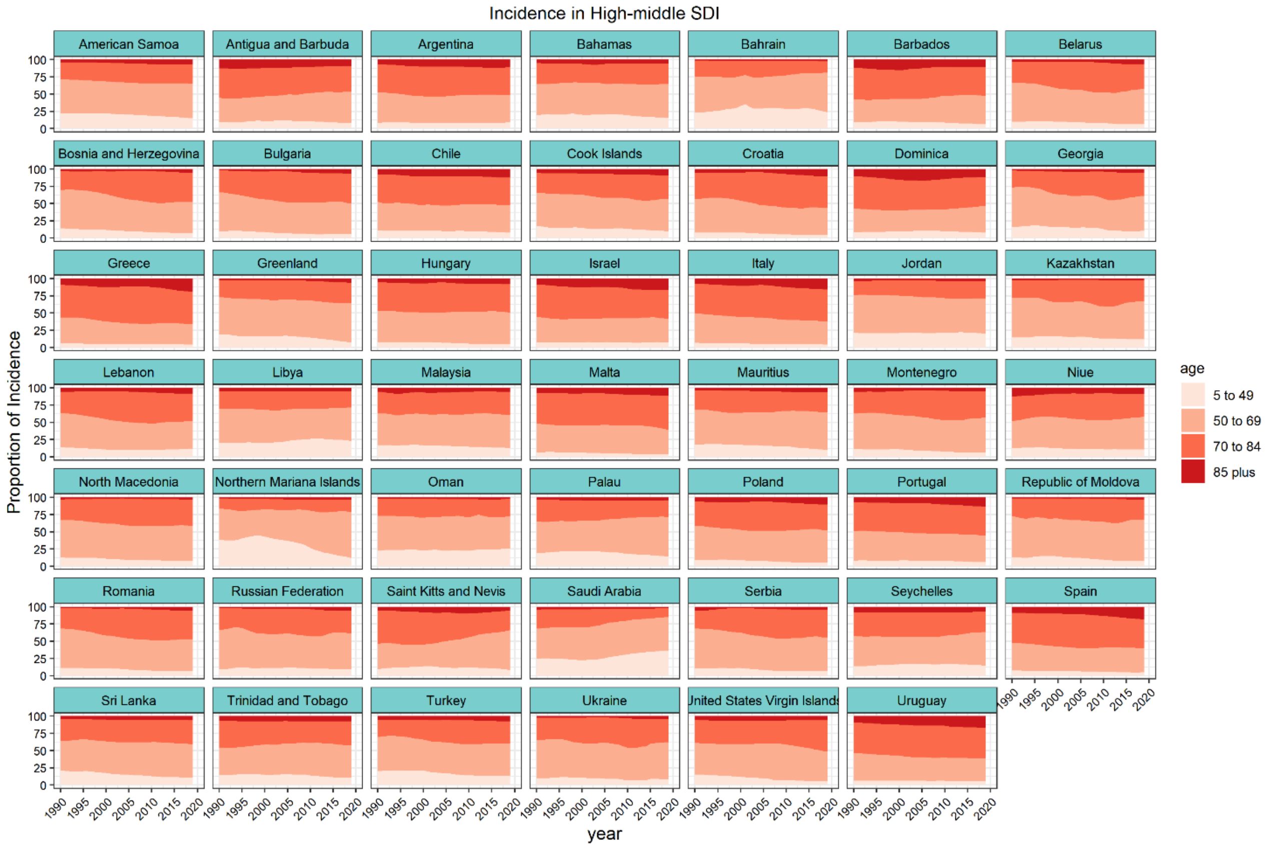

Figure S5-A. The local drifts of colorectal cancer incidence in middle-SDI countries, 1990-2019.

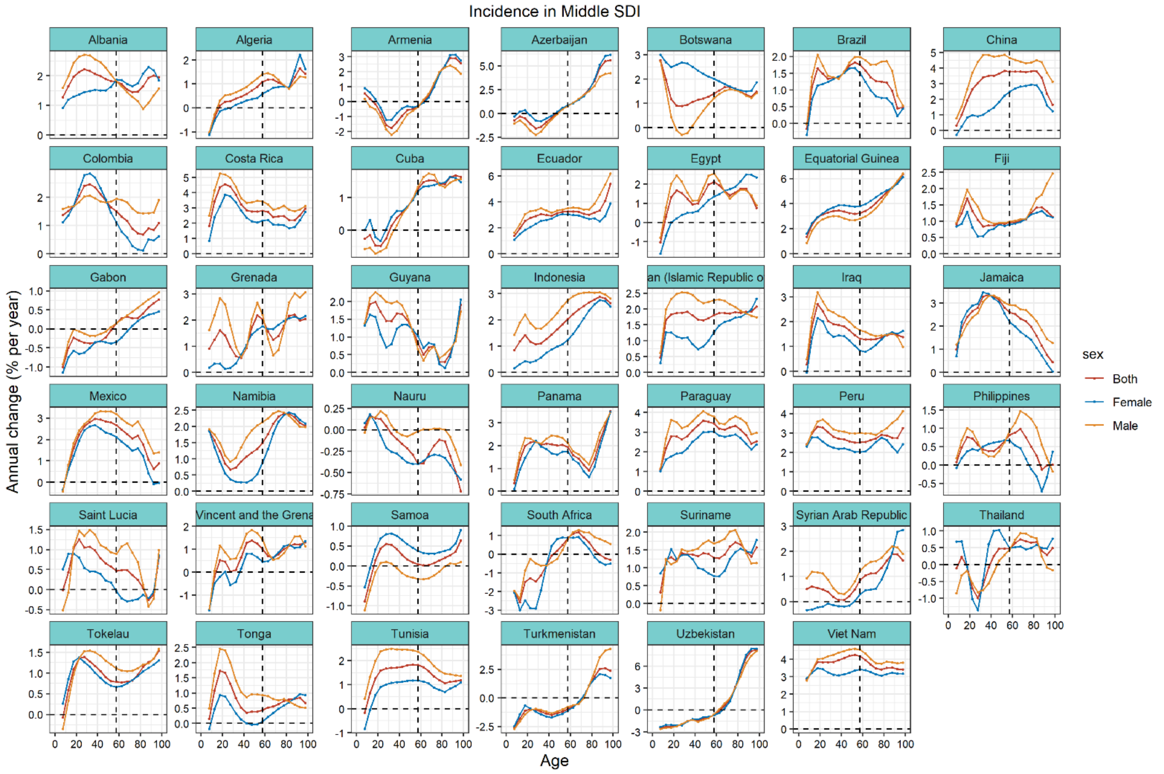

Local drifts of colorectal cancer incidence (estimates from age-period-cohort models) for 19 age groups (5–9 to 95 plus years), 1990–2019. The dots and shaded areas indicate the annual percentage change of incidence (% per year) and the corresponding 95% CIs.

Figure S5-B. Age distribution of absolute cases of colorectal cancer incidence in middle-SDI countries, 1990-2019

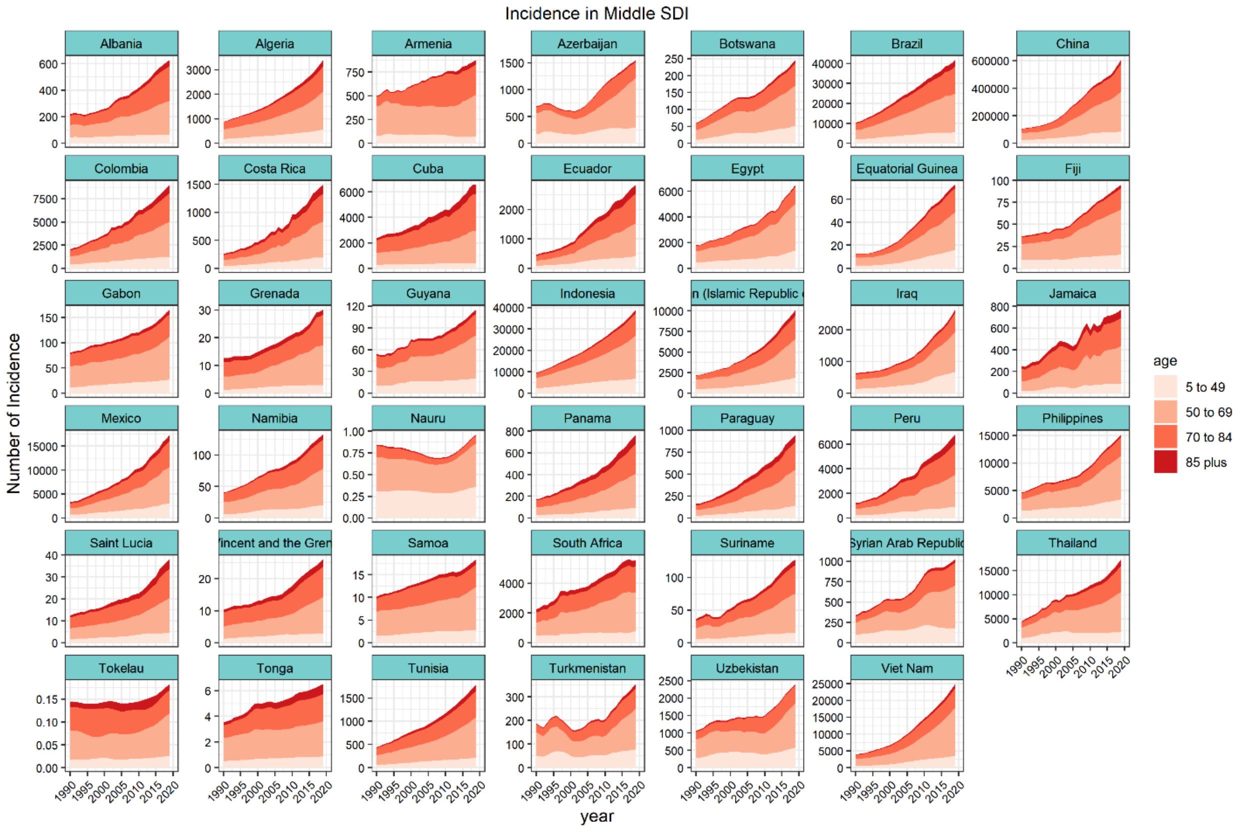

Figure S5-C. Age distribution of relative proportion of colorectal cancer incidence in middle-SDI countries, 1990-2019

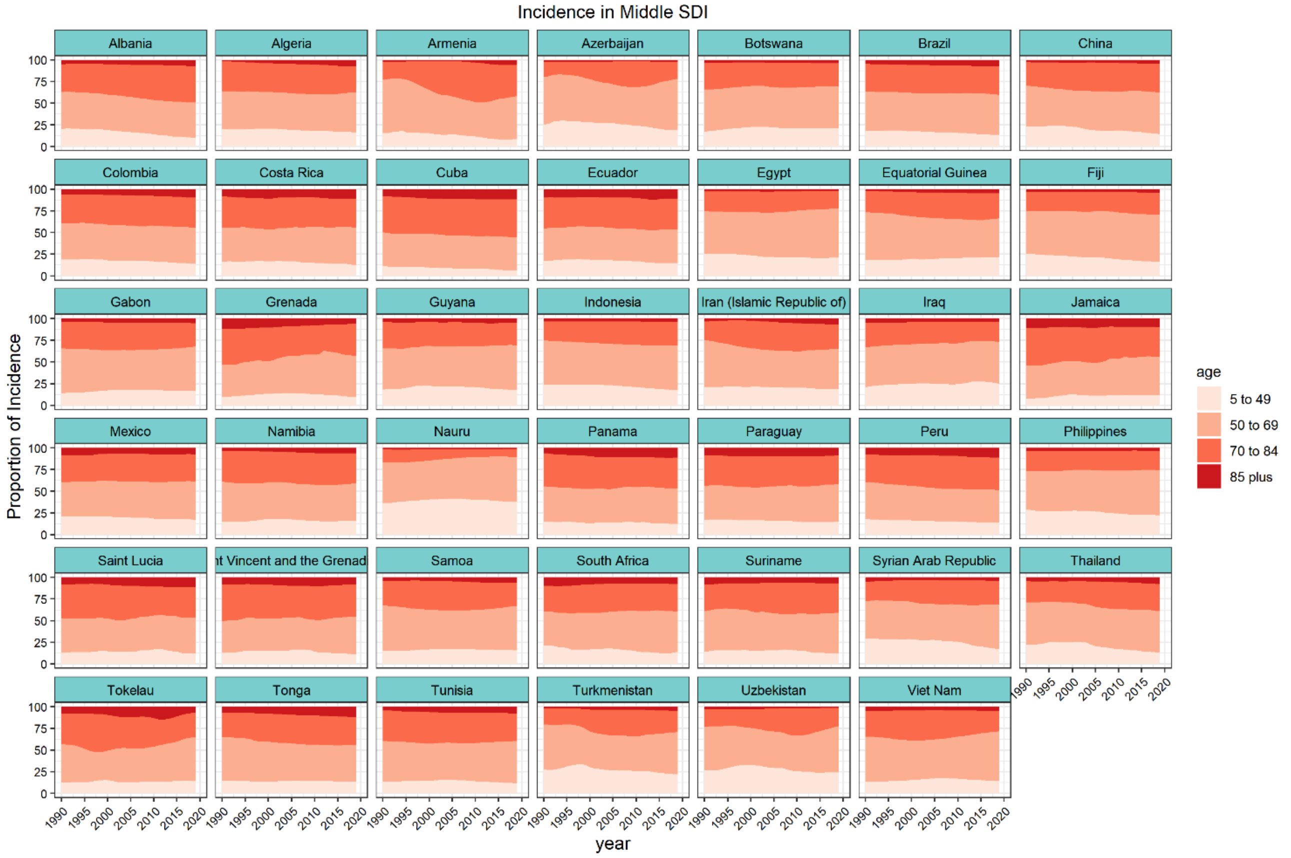

Figure S6-A. The local drifts of colorectal cancer incidence in low-middle-SDI countries, 1990-2019.

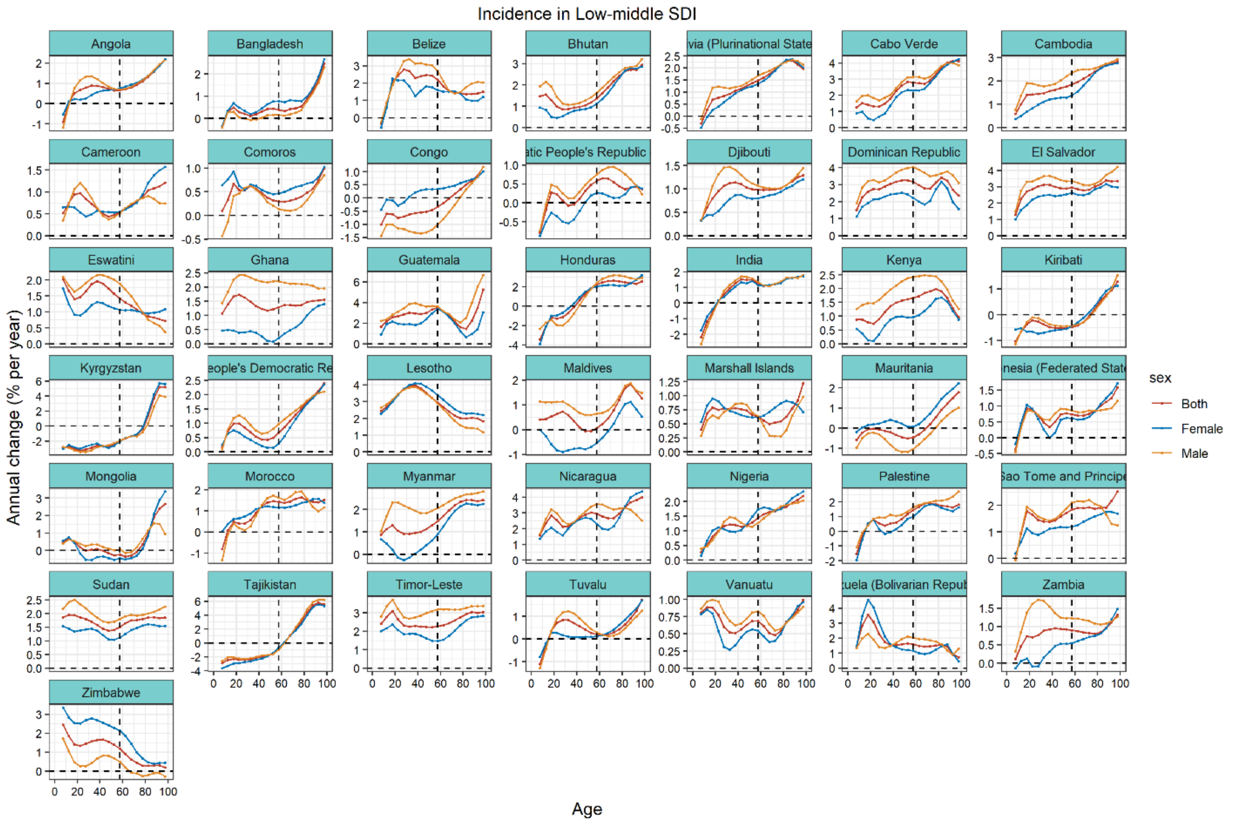

Local drifts of colorectal cancer incidence (estimates from age-period-cohort models) for 19 age groups (5−9 to 95 plus years), 1990−2019. The dots and shaded areas indicate the annual percentage change of incidence (% per year) and the corresponding 95% CIs.

Figure S6-B. Age distribution of absolute cases of colorectal cancer incidence in low-middle-SDI countries, 1990-2019

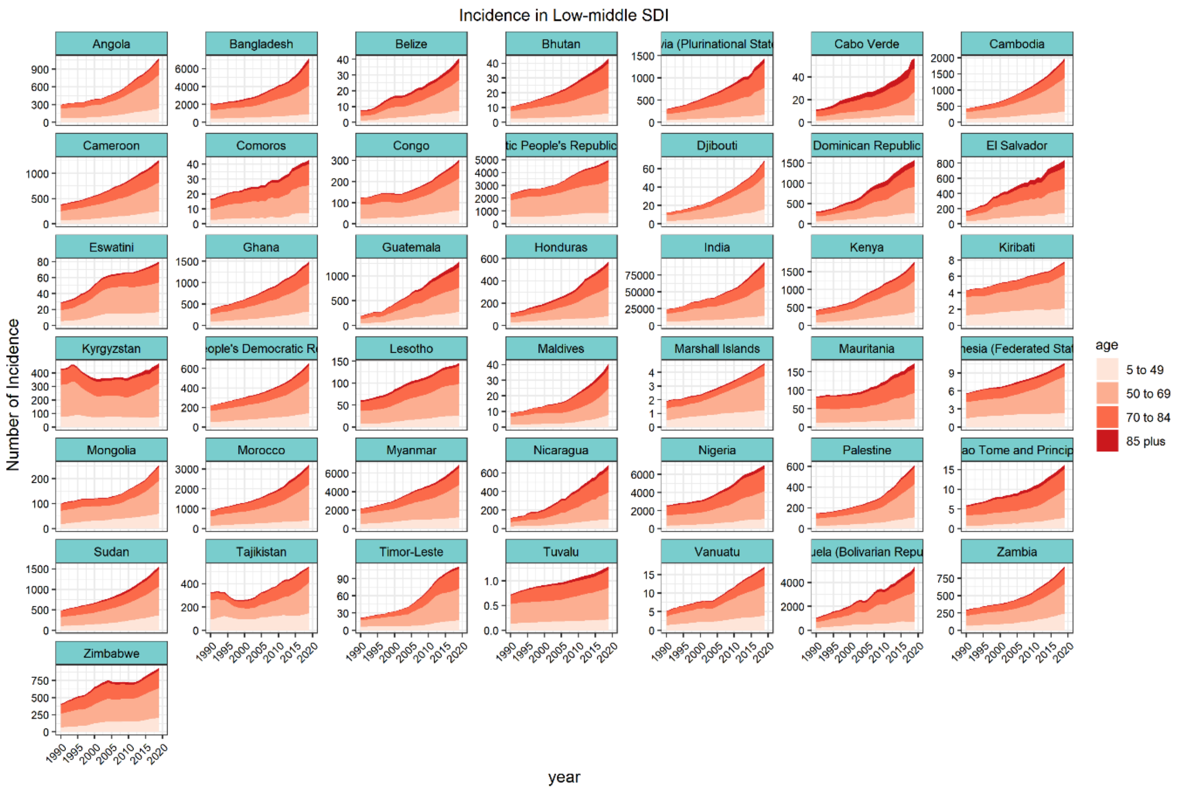

Figure S6-C. Age distribution of relative proportion of colorectal cancer incidence in low-middle-SDI countries, 1990-2019

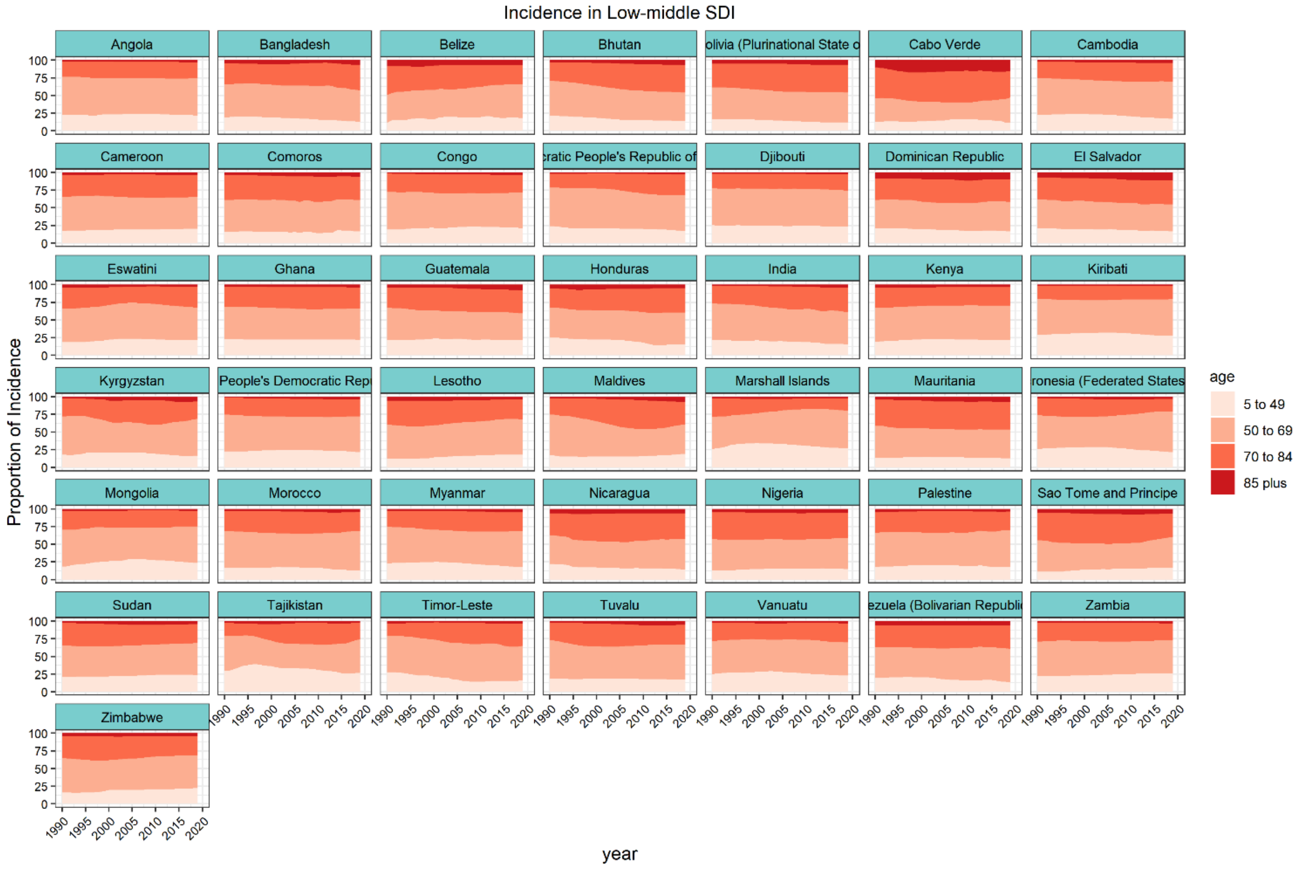

Figure S7-A. The local drifts of colorectal cancer incidence in low -SDI countries, 1990-2019.

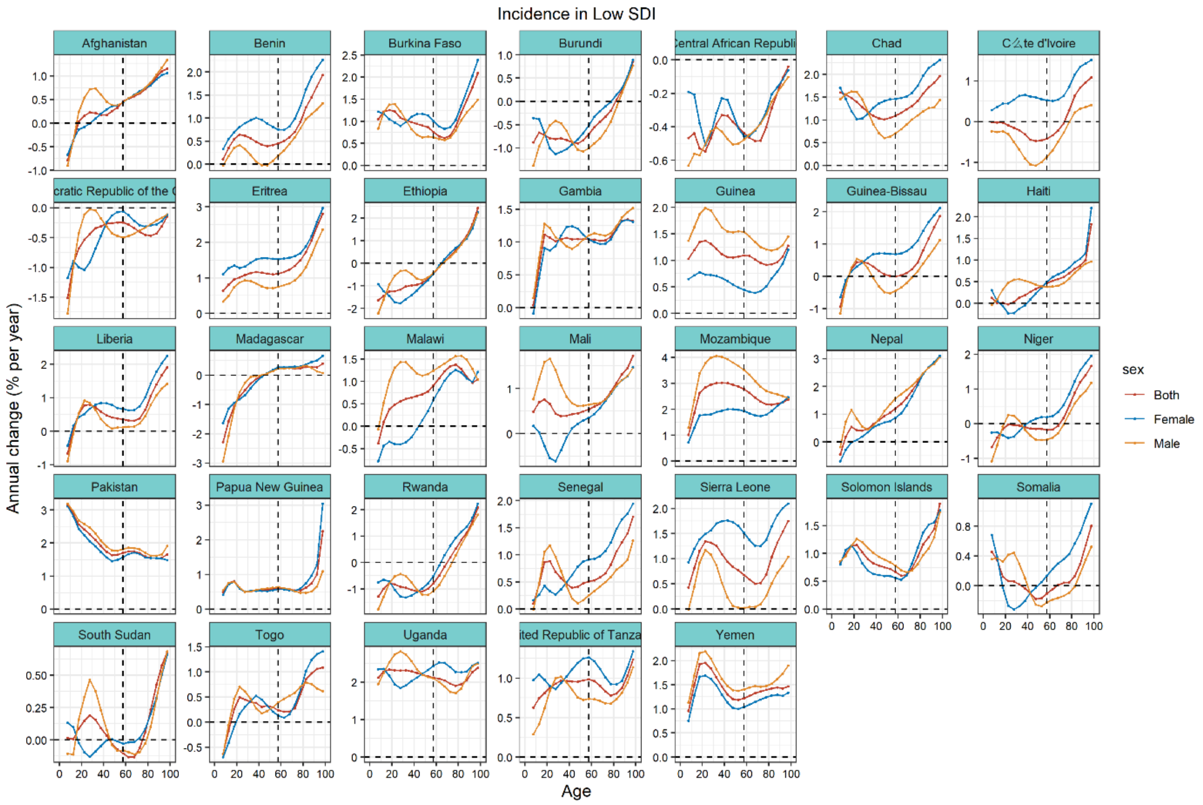

Local drifts of colorectal cancer incidence (estimates from age-period-cohort models) for 19 age groups (5−9 to 95 plus years), 1990−2019. The dots and shaded areas indicate the annual percentage change of incidence (% per year) and the corresponding 95% CIs.

Figure S7-B. Age distribution of absolute cases of colorectal cancer incidence in low -SDI countries, 1990-2019

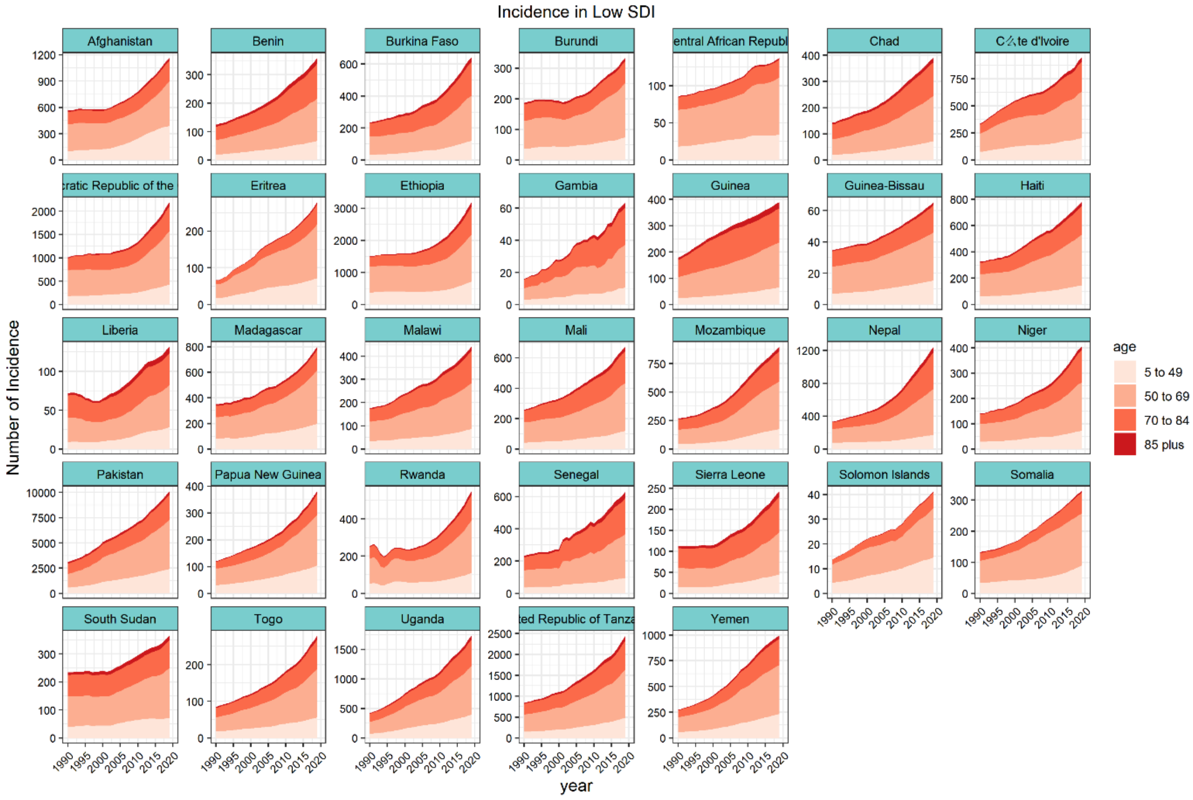

Figure S7-C. Age distribution of relative proportion of colorectal cancer incidence in low -SDI countries, 1990-2019

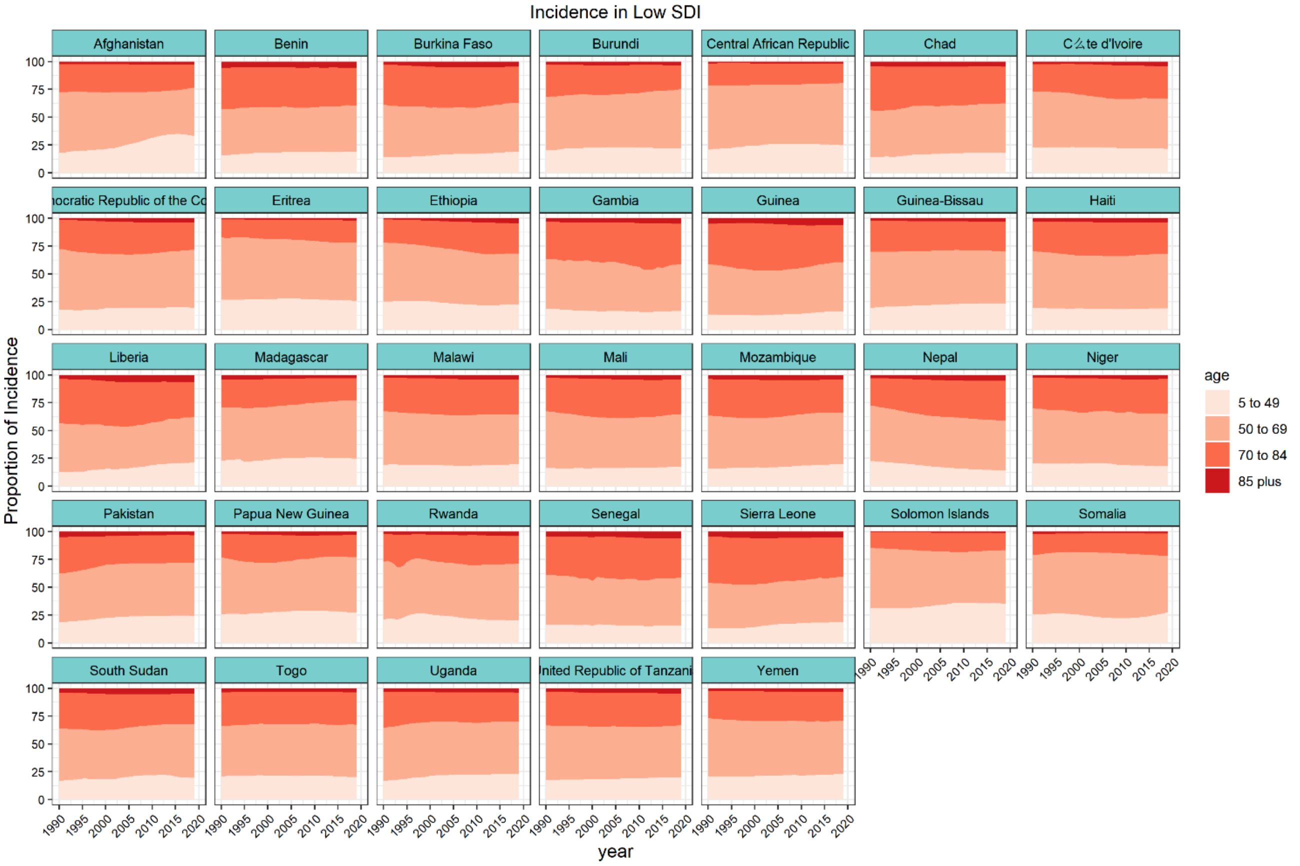

FigureS8. Incidence rates of colorectal cancer across different age groups, periods and birth cohorts by SDI quintiles during 1990-2019.

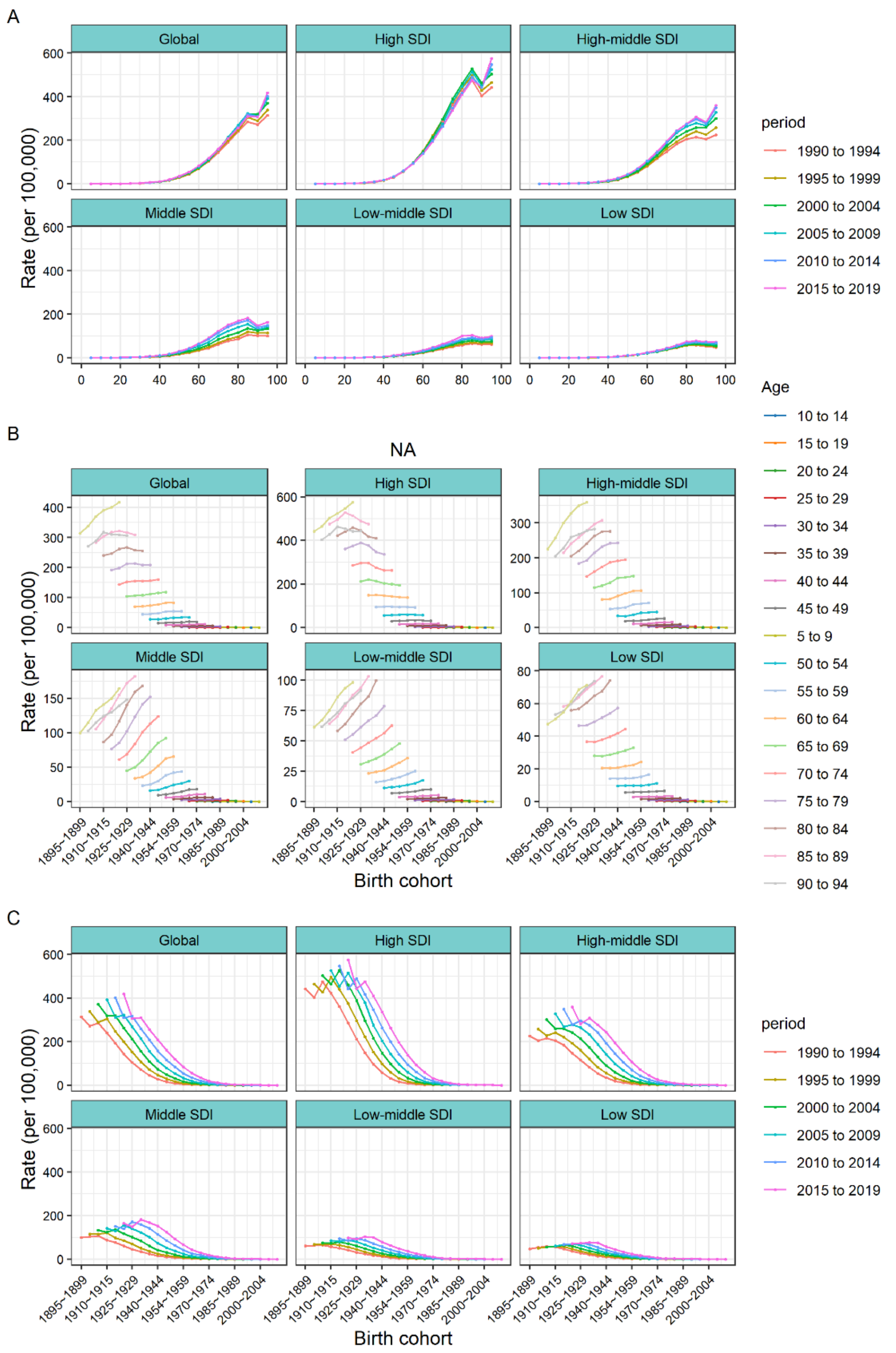

(A) Incidence rates of colorectal cancer across age groups by periods.

(B) Incidence rates of colorectal cancer across birth cohorts by age groups.

(C) Incidence rates of colorectal cancer across birth cohorts by periods

FigureS9. Incidence rates of colorectal cancer across different age groups, periods and birth cohorts in 21GBD regions during 1990-2019.

A.

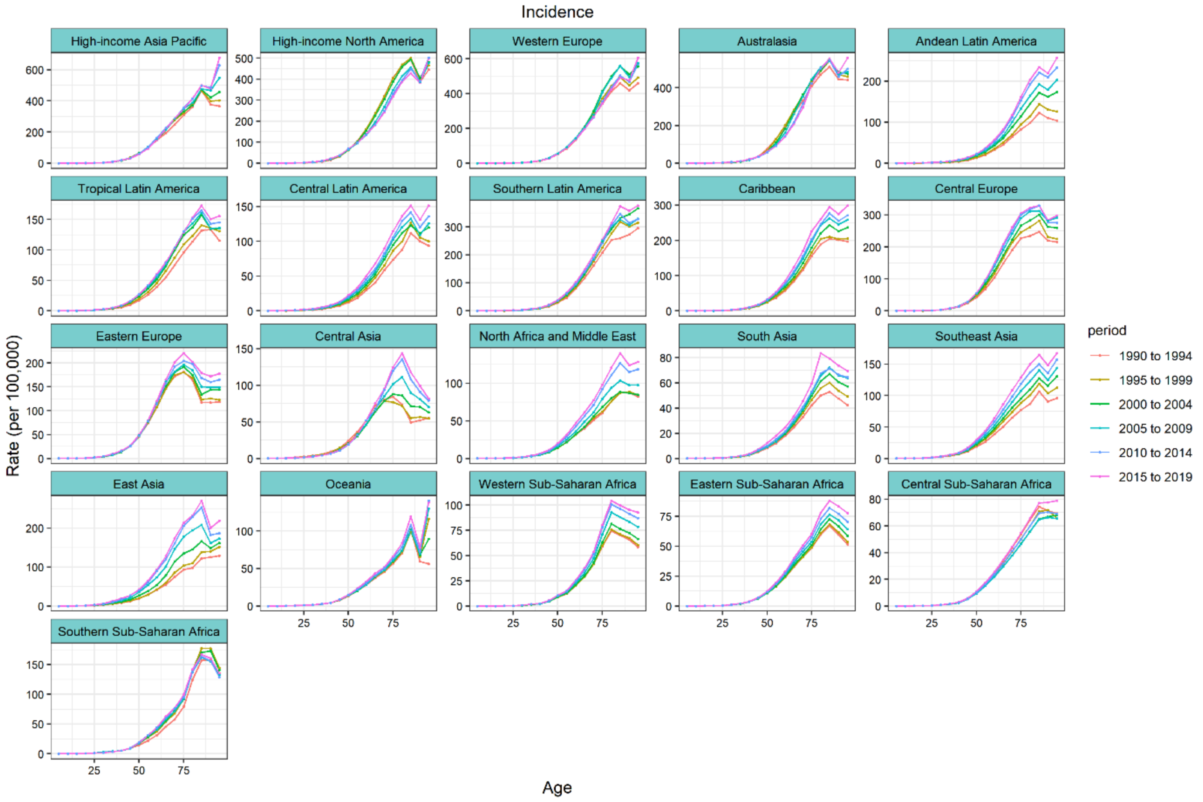

B

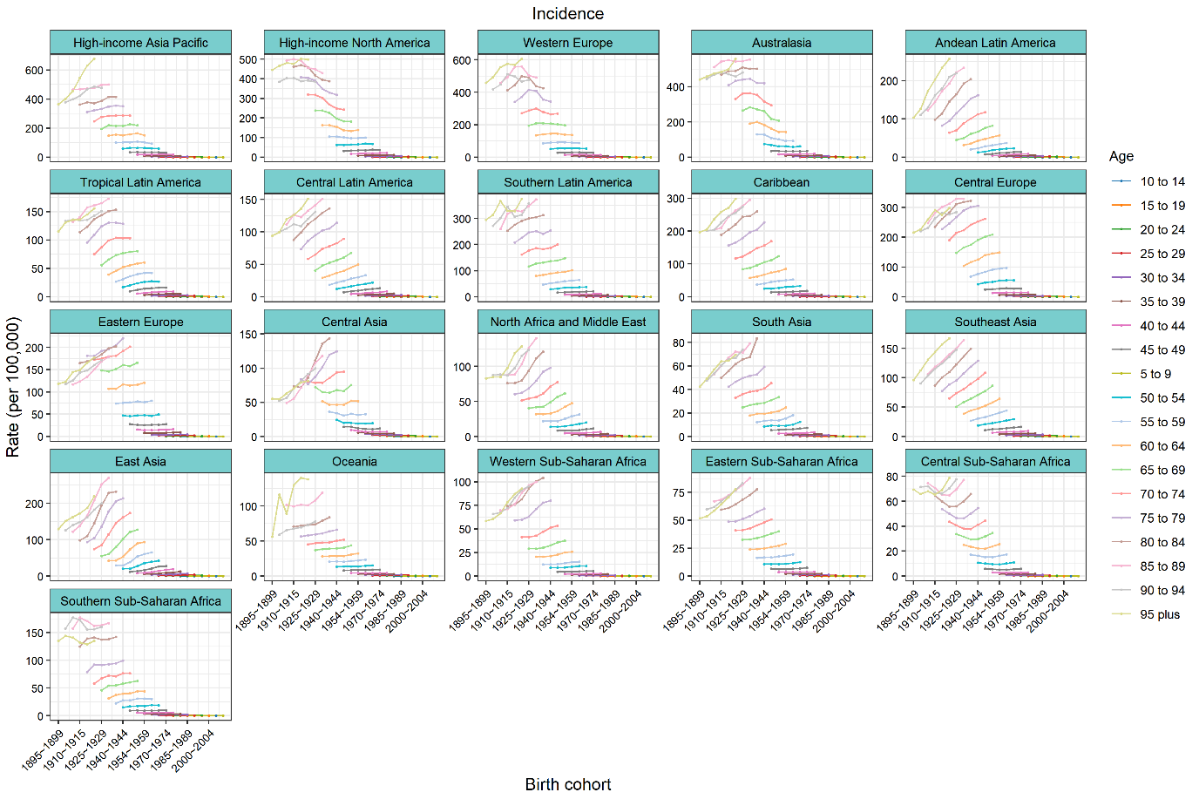

C

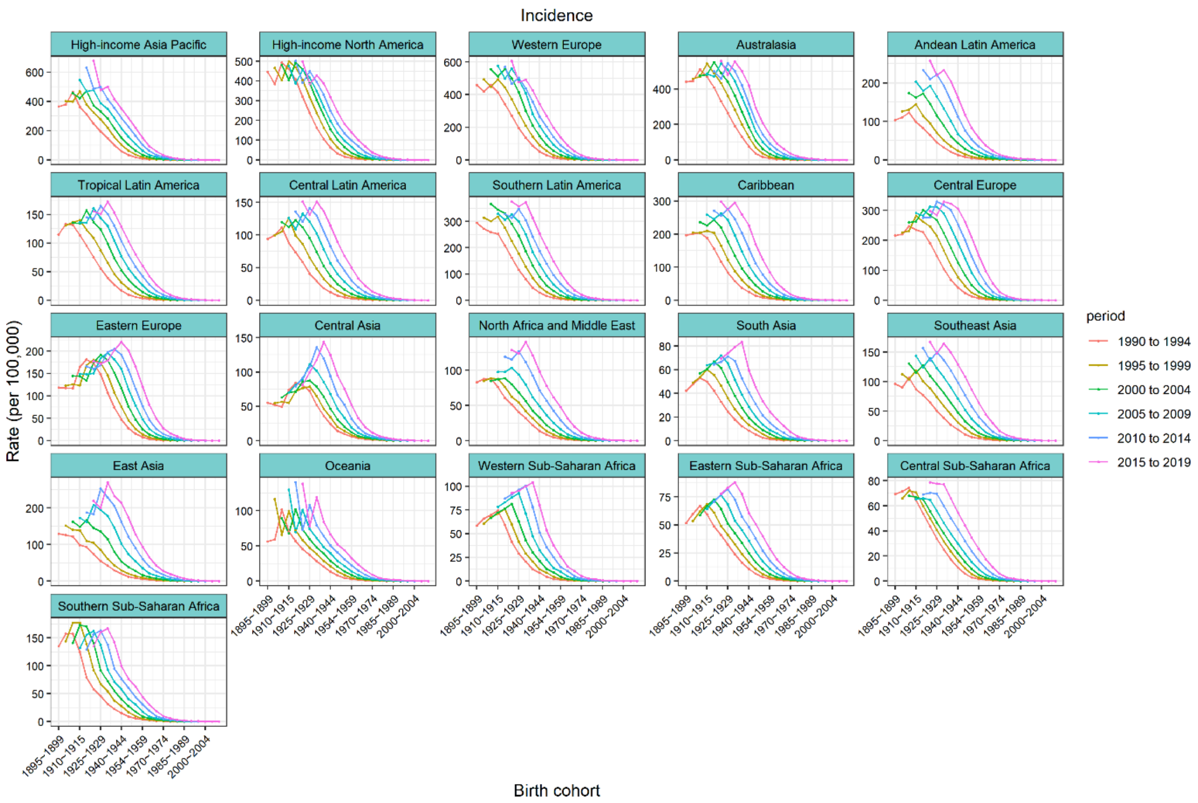

(A) Incidence rates of colorectal cancer across different age groups by periods in 21GBD regions, 1990-2019.

(B) Incidence rates of colorectal cancer across different birth cohorts by age groups in 21GBD regions, 1990-2019.

(C) Incidence rates of colorectal cancer across different birth cohorts by periods in 21GBD regions, 1990-2019.

FigureS10. Age-period-cohort effects on colorectal cancer incidence in 21GBD regions.

A

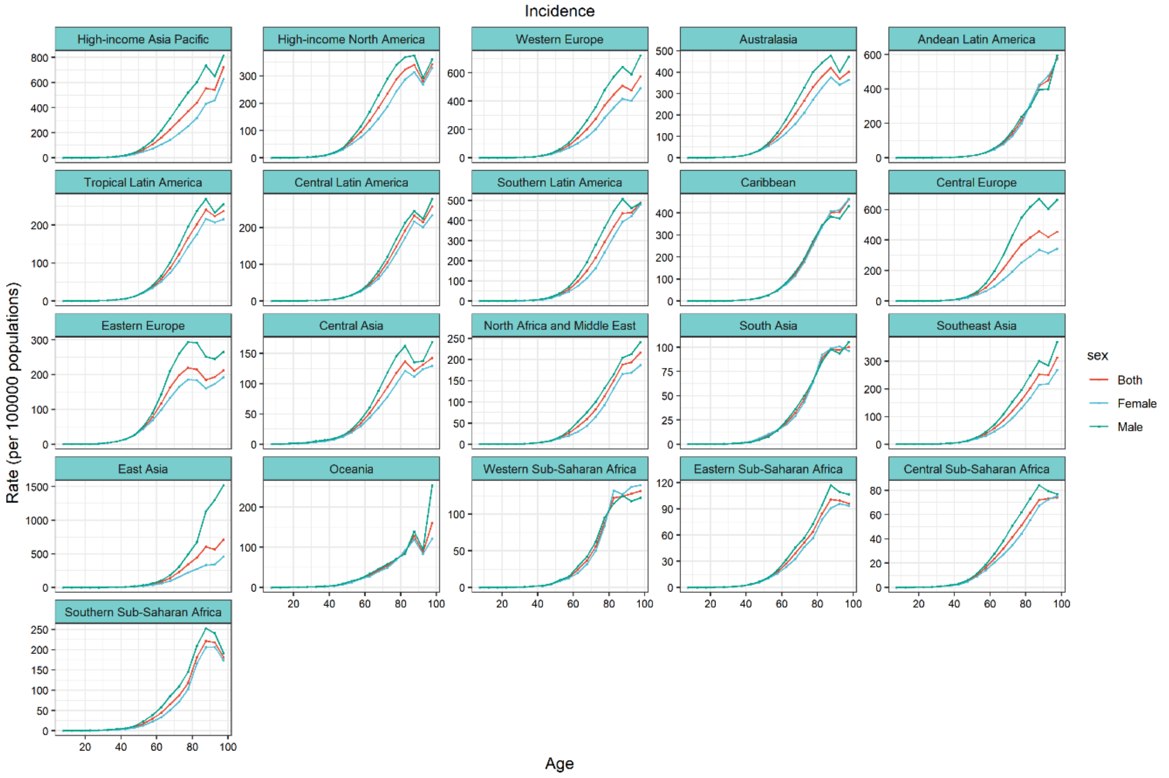

B

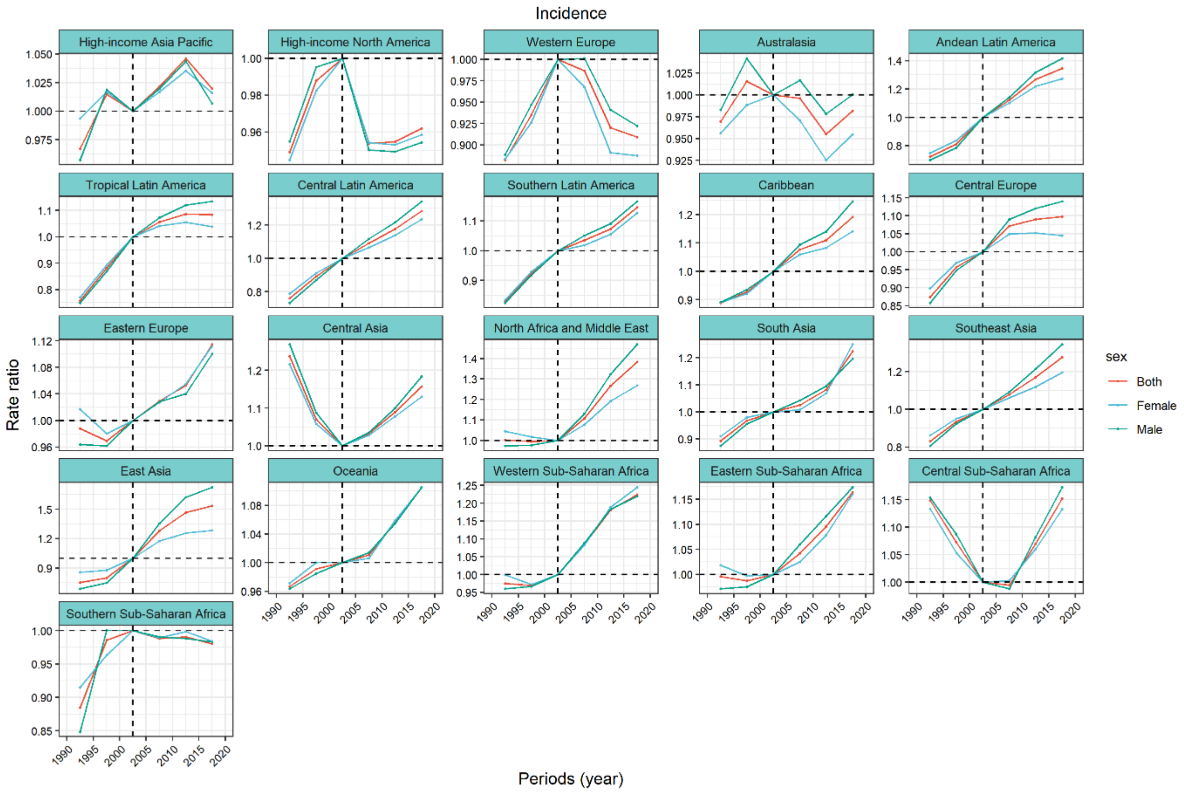

C

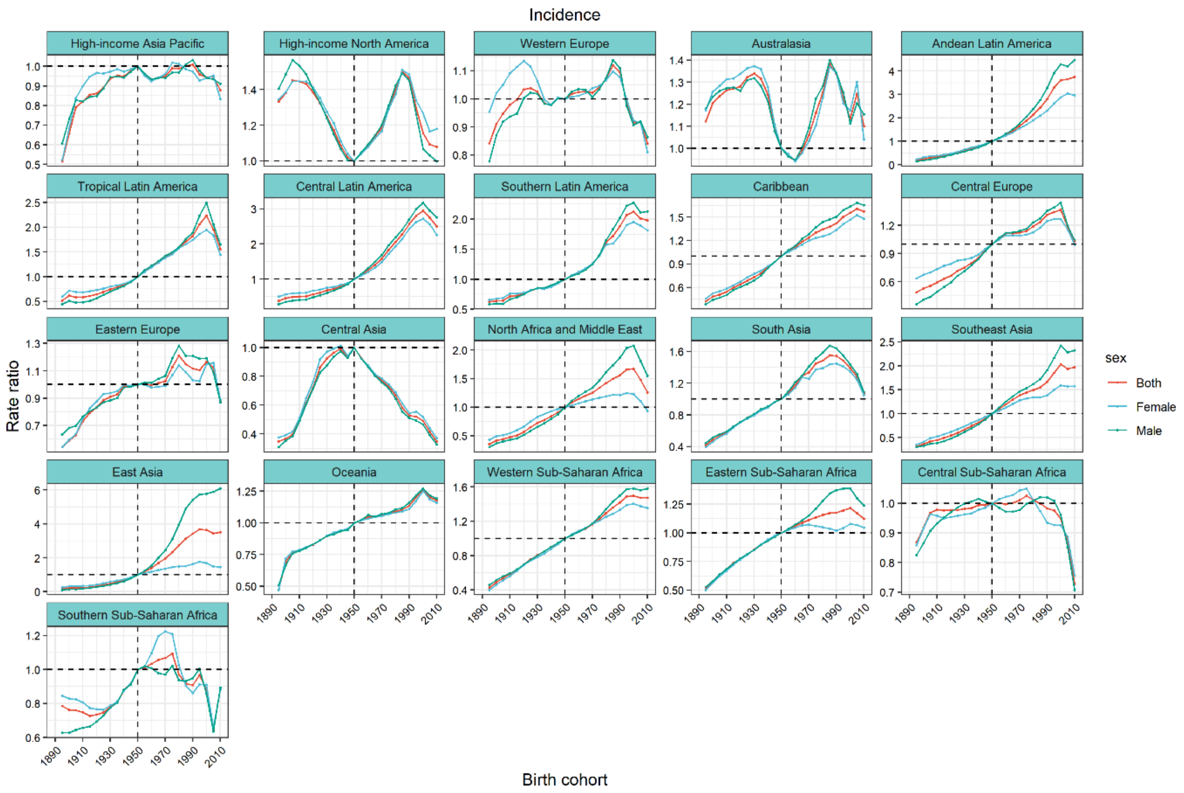

(A) Age effects are shown by the fitted longitudinal age curves of incidence (per 100,000 person-years) adjusted for period deviations.

(B) Period effects are shown by the relative risk of incidence (incidence rate ratio) and computed as the ratio of age-specific rates from 1990−1994 to 2015−2019 (2000−2005 as the referent period).

(C)Cohort effects are shown by the relative risk of incidence and computed as the ratio of age-specific rates from the 1895 cohort to the 2010 cohort, with the referent cohort set at 1950.

FigureS11. Incidence rates of colorectal cancer across different age groups, periods and birth cohorts in high-SDI countries during 1990-2019.

A

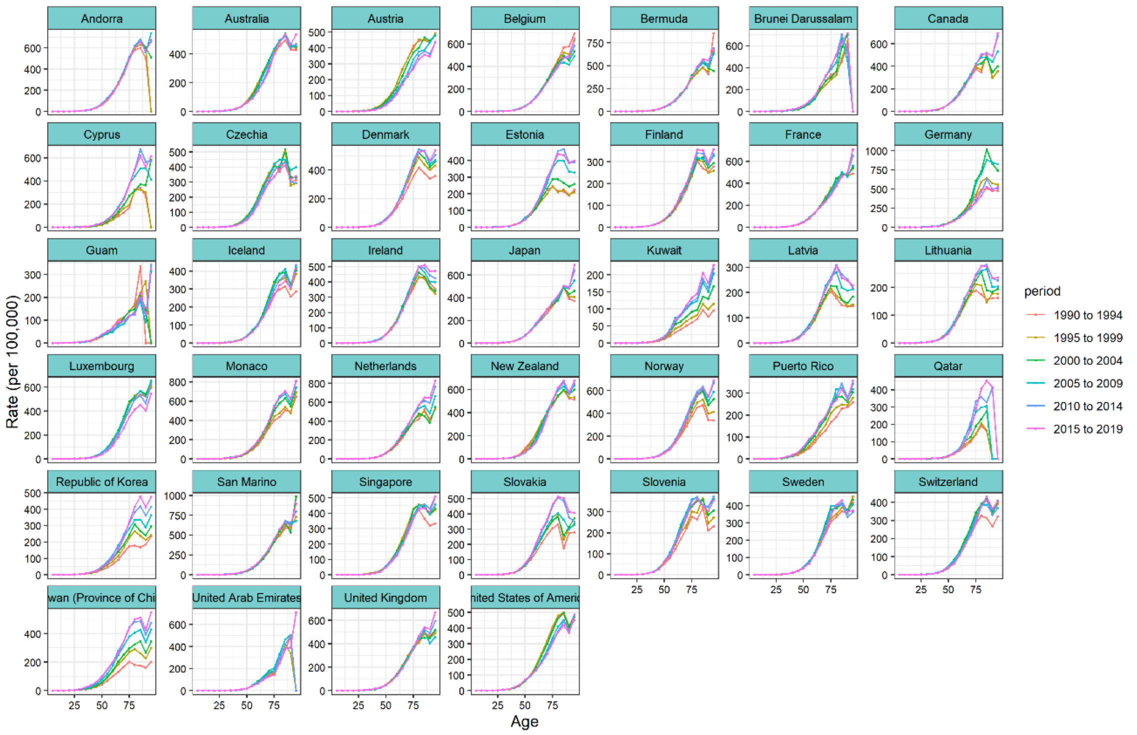

B

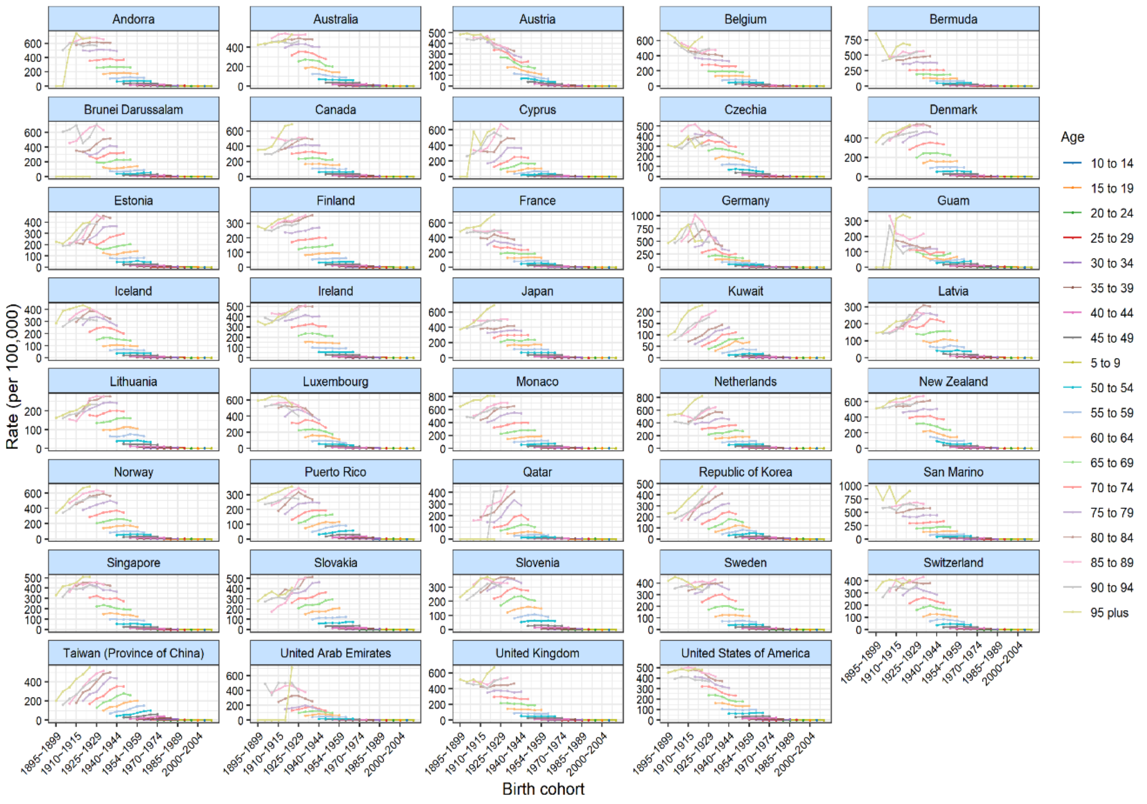

C

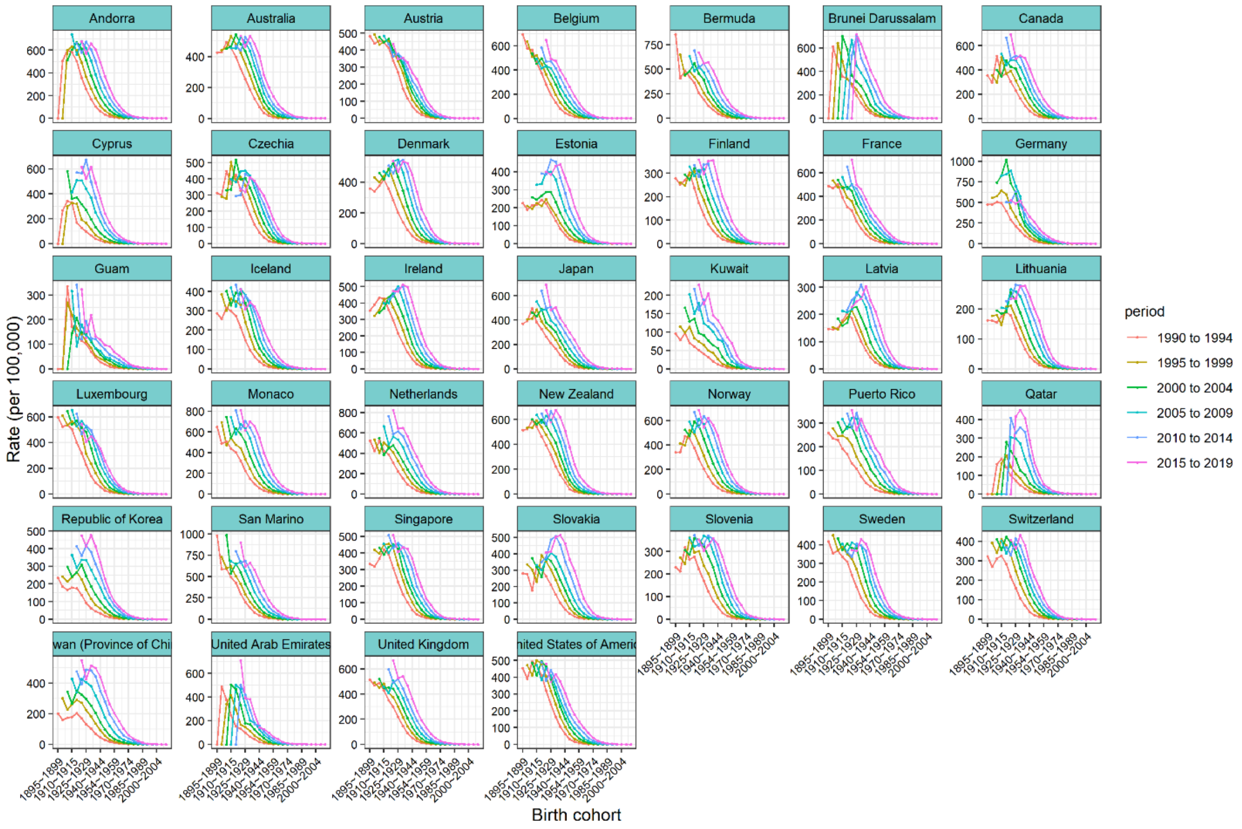

(A) Incidence rates of colorectal cancer across different age groups by periods in high-SDI countries, 1990-2019.

(B) Incidence rates of colorectal cancer across different birth cohorts by age groups in high-SDI countries, 1990-2019.

(C) Incidence rates of colorectal cancer across different birth cohorts by periods in high-SDI countries, 1990-2019.

FigureS12. Age-period-cohort effects on colorectal cancer incidence in high-SDI countries.

A

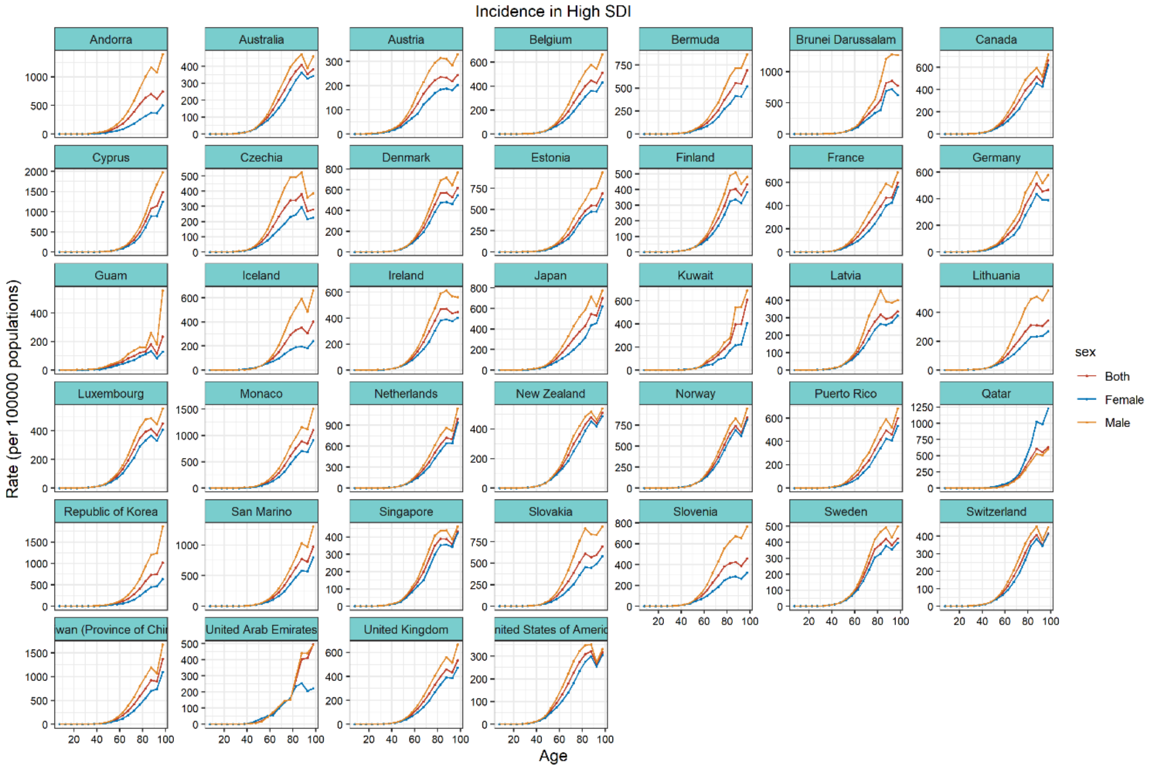

B

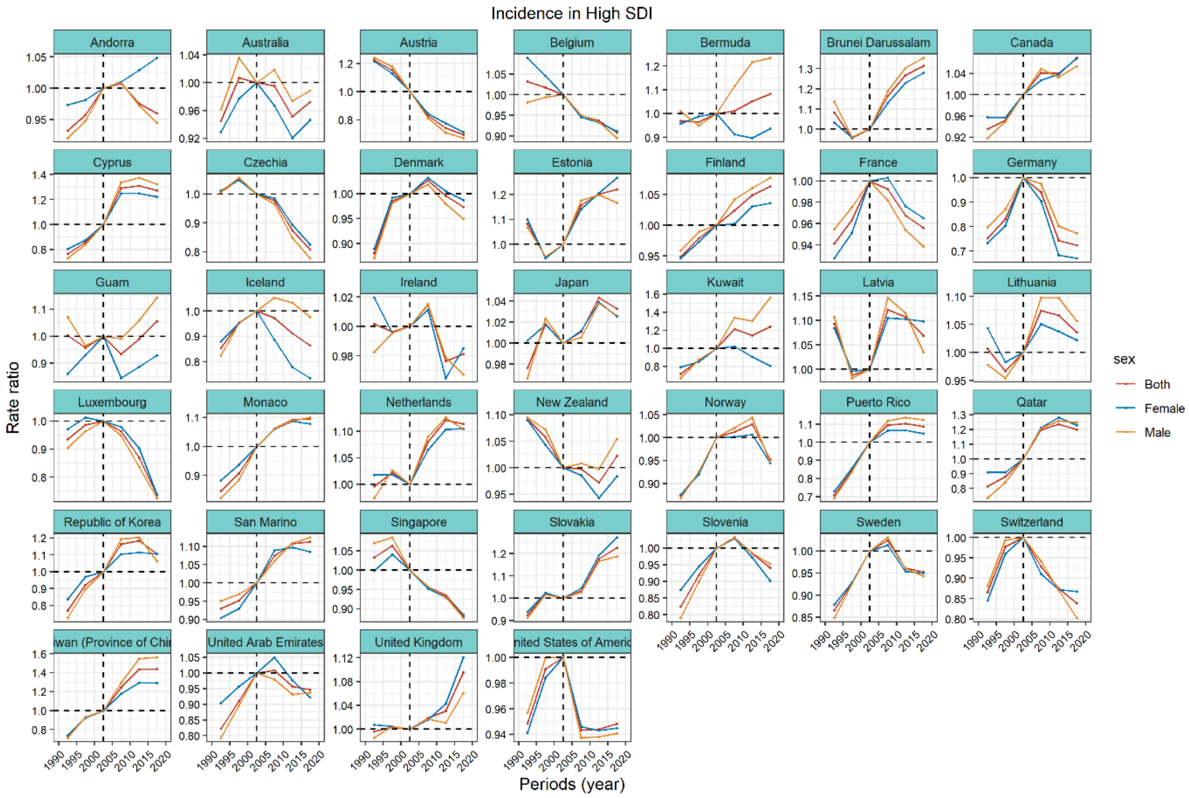

C

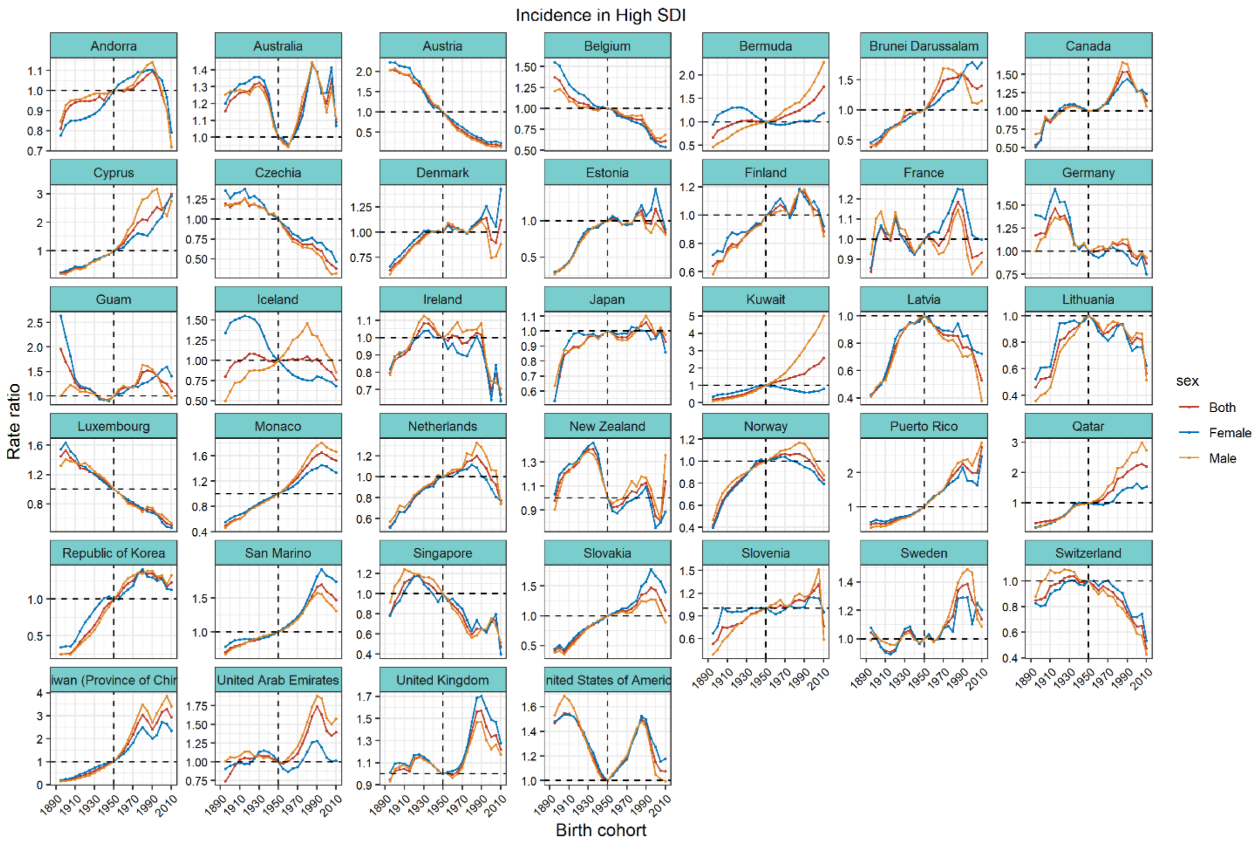

(A) Age effects are shown by the fitted longitudinal age curves of incidence (per 100,000 person-years) adjusted for period deviations.

(B) Period effects are shown by the relative risk of incidence (incidence rate ratio) and computed as the ratio of age-specific rates from 1990−1994 to 2015−2019 (2000−2005 as the referent period).

(C)Cohort effects are shown by the relative risk of incidence and computed as the ratio of age-specific rates from the 1895 cohort to the 2010 cohort, with the referent cohort set at 1950.

FigureS13. Incidence rates of colorectal cancer across different age groups, periods and birth cohorts in high-middle-SDI countries during 1990-2019.

A

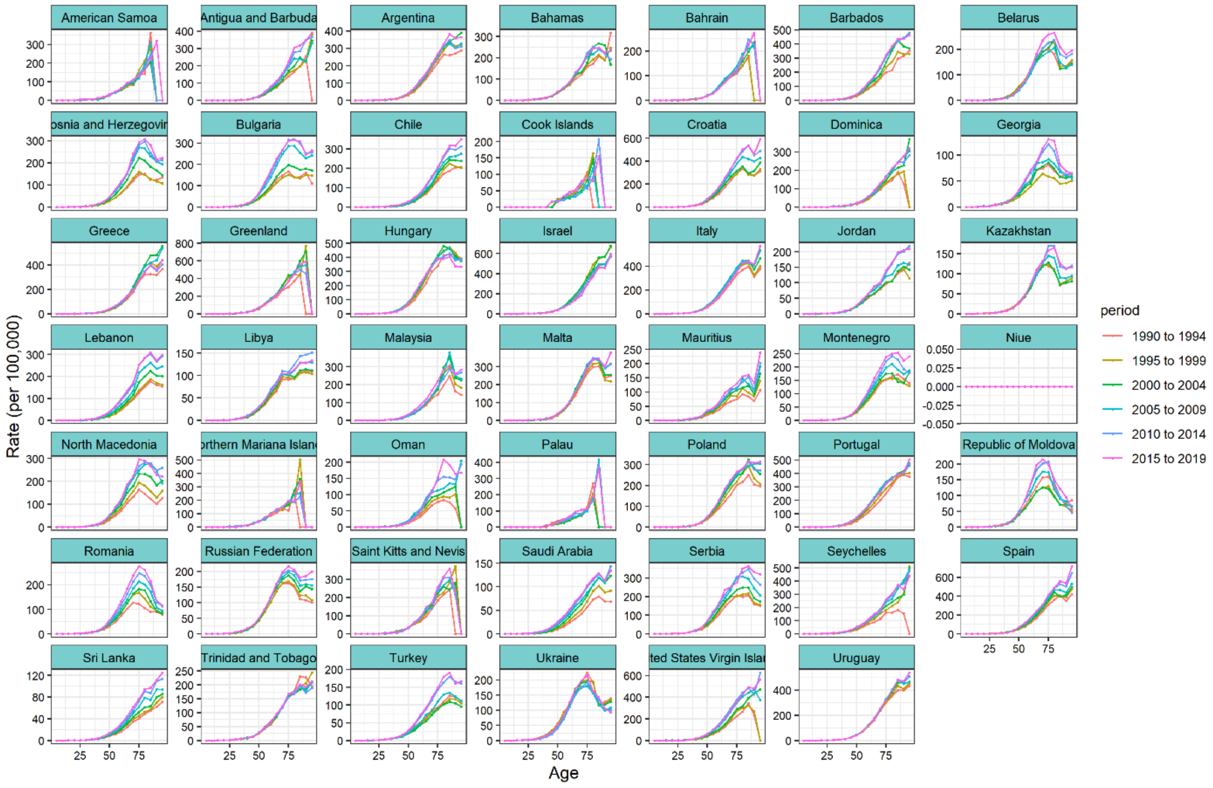

B

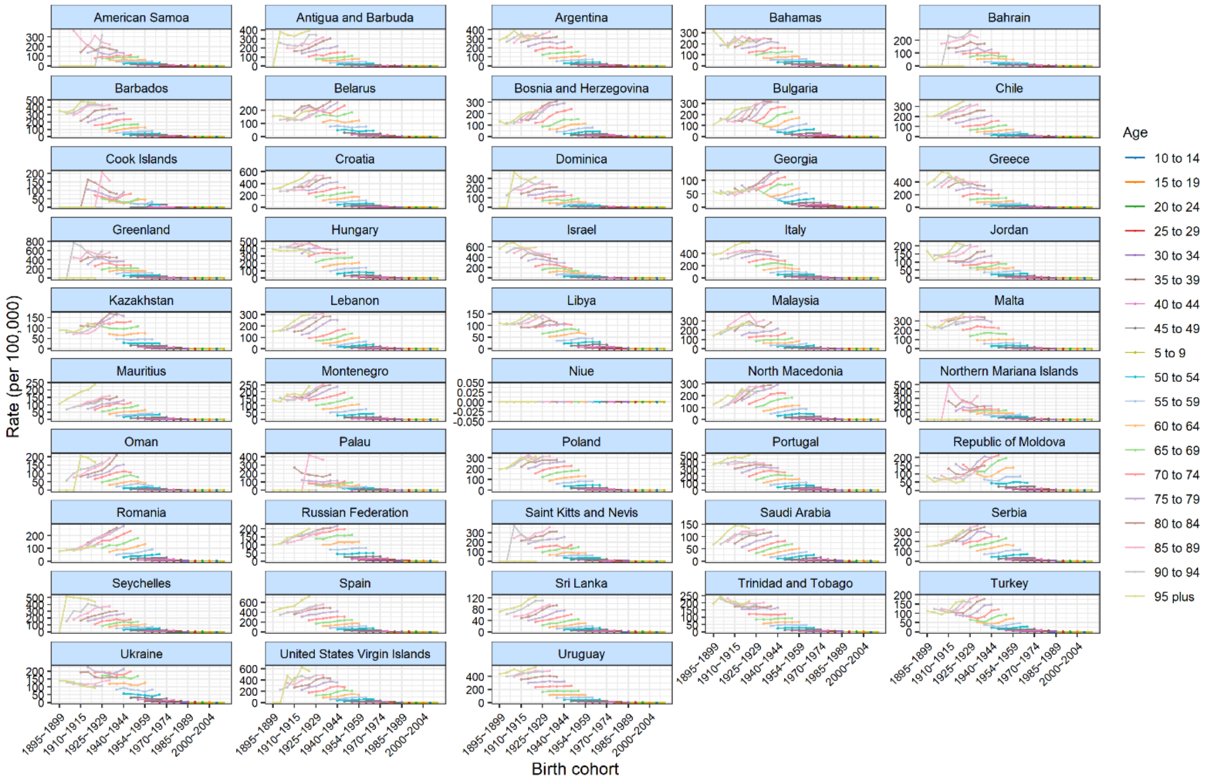

C

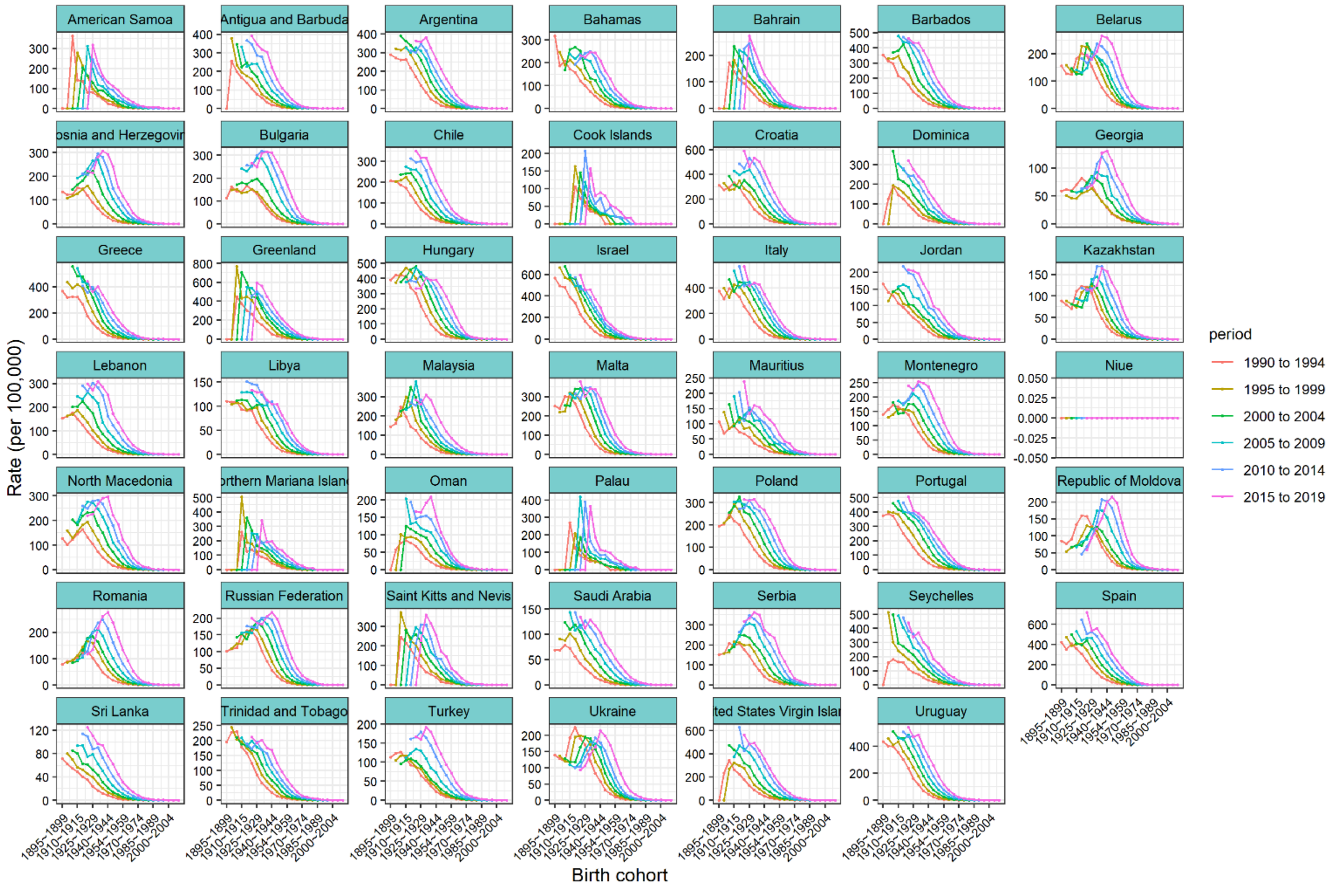

(A) Incidence rates of colorectal cancer across different by periods in high-middle-SDI countries, 1990-2019.

(B) Incidence rates of colorectal cancer across different birth cohorts by age groups in high-middle-SDI countries, 1990-2019.

(C) Incidence rates of colorectal cancer across different birth cohorts by periods in high-middle-SDI countries, 1990-2019.

FigureS14. Age-period-cohort effects on colorectal cancer incidence in high-middle-SDI countries.

A

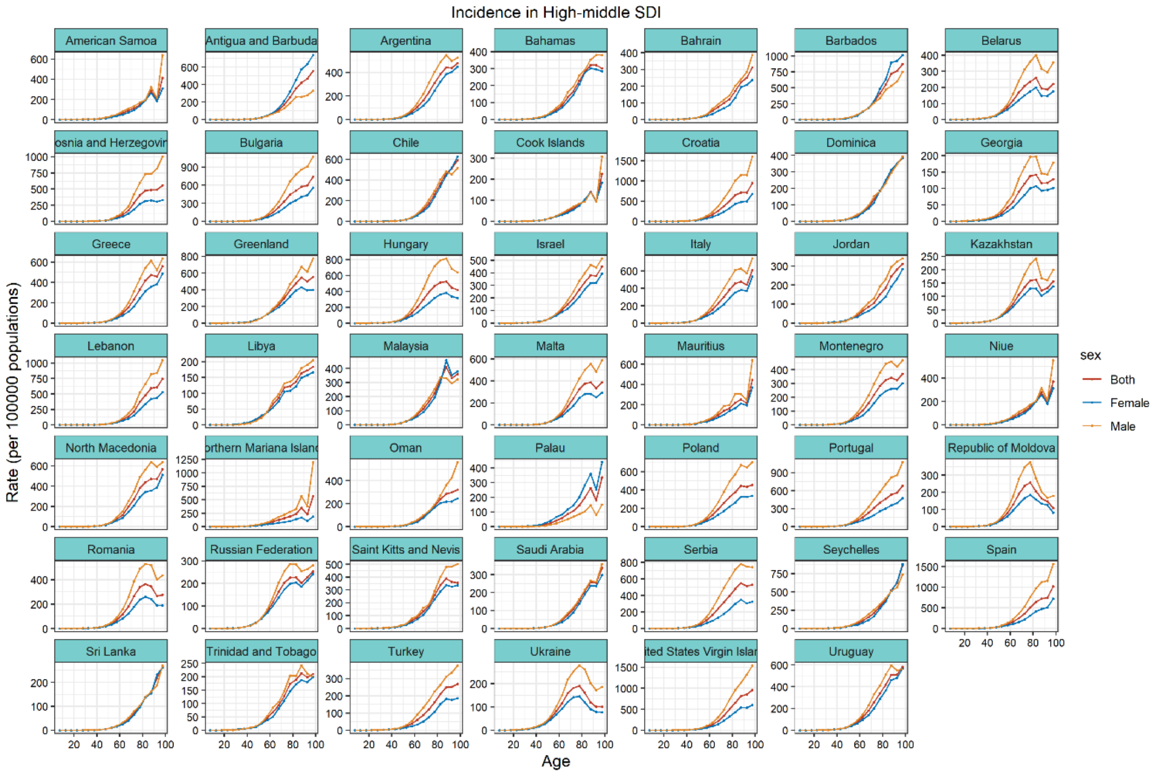

B

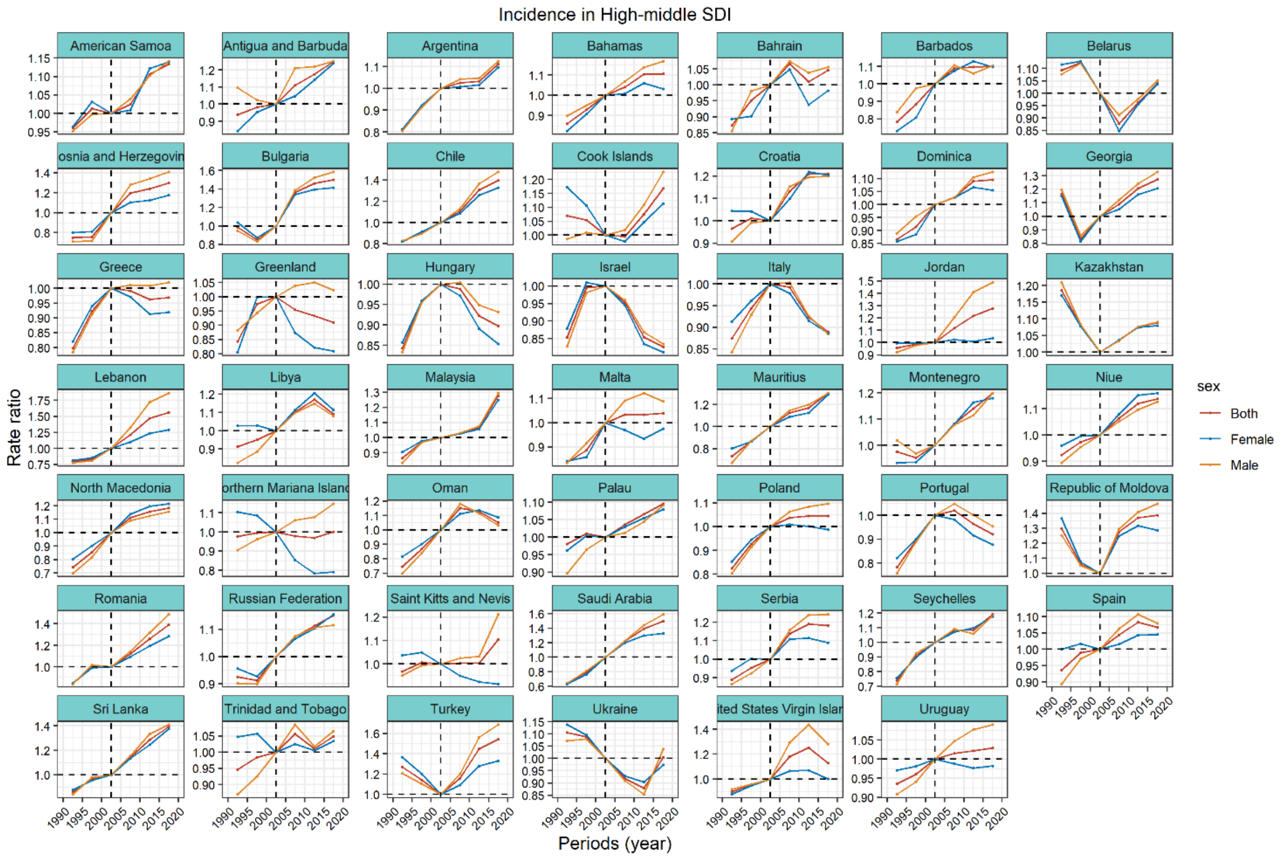

C

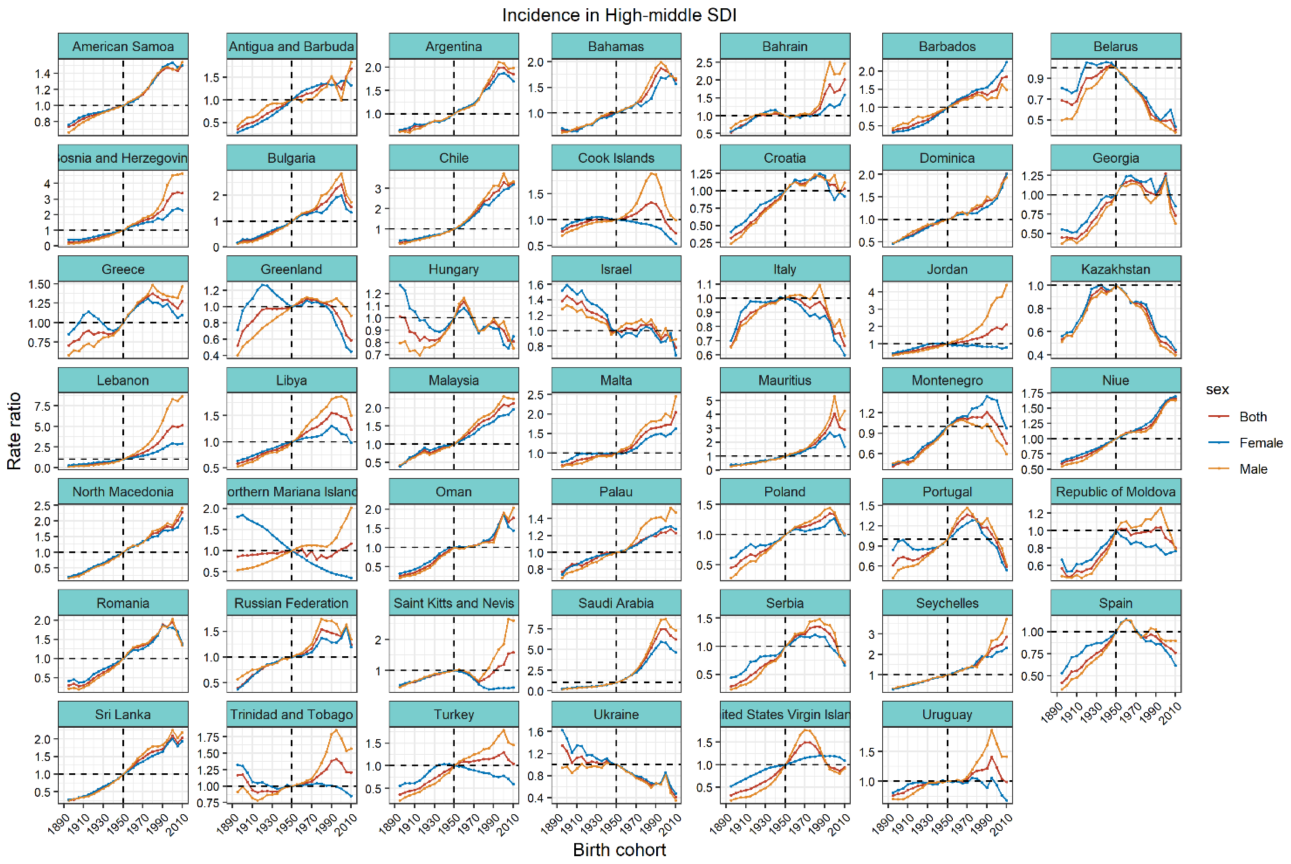

(A) Age effects are shown by the fitted longitudinal age curves of incidence (per 100,000 person-years) adjusted for period deviations.

(B) Period effects are shown by the relative risk of incidence (incidence rate ratio) and computed as the ratio of age-specific rates from 1990−1994 to 2015−2019 (2000−2005 as the referent period).

(C)Cohort effects are shown by the relative risk of incidence and computed as the ratio of age-specific rates from the 1895 cohort to the 2010 cohort, with the referent cohort set at 1950.

FigureS15. Incidence rates of colorectal cancer across different age groups, periods and birth cohorts in middle-SDI countries during 1990-2019.

A

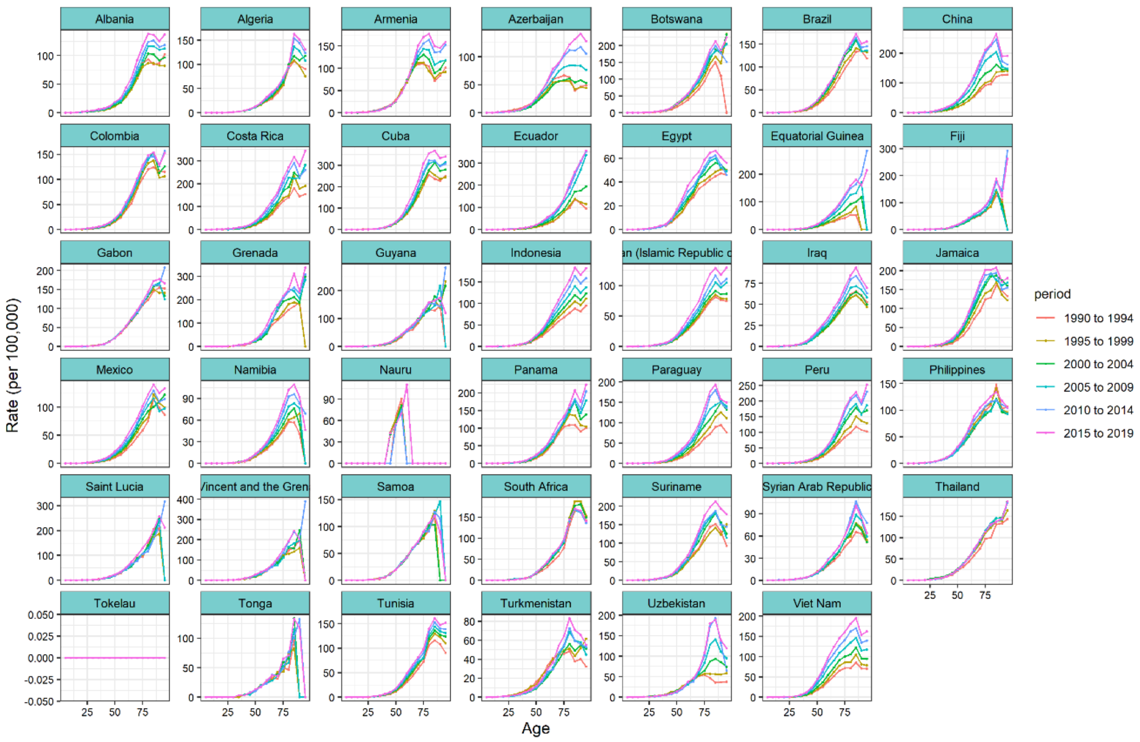

B

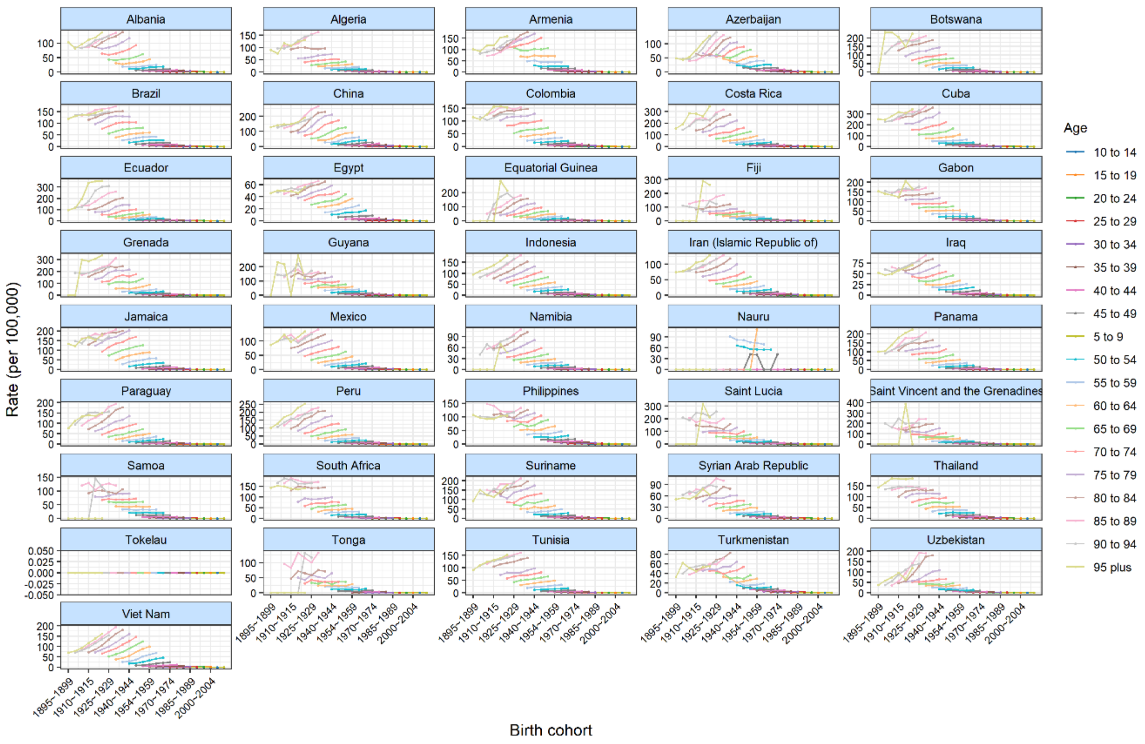

C

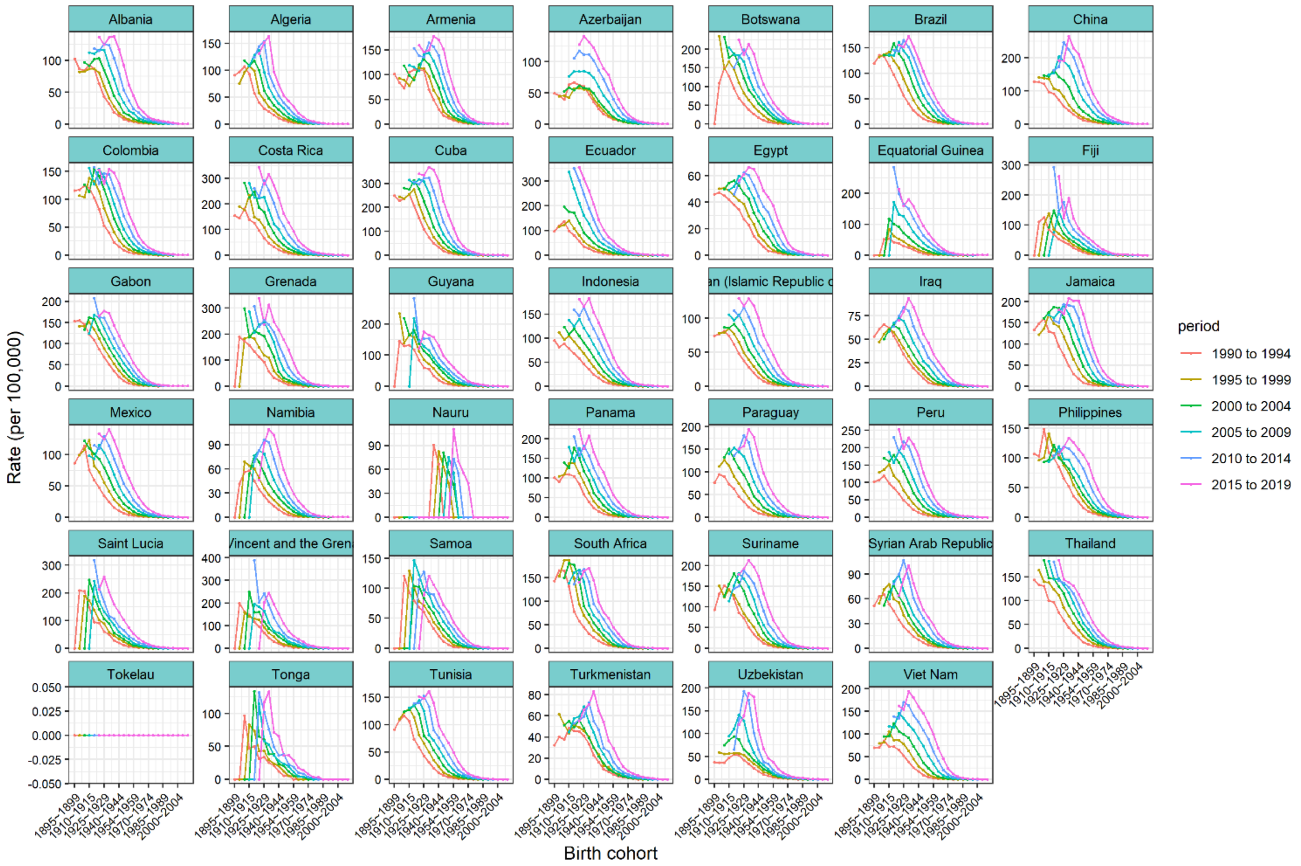

(A) Incidence rates of colorectal cancer across different by periods in middle-SDI countries, 1990-2019.

(B) Incidence rates of colorectal cancer across different birth cohorts by age groups in middle-SDI countries, 1990-2019.

(C) Incidence rates of colorectal cancer across different birth cohorts by periods in middle-SDI countries, 1990-2019.

FigureS16. Age-period-cohort effects on colorectal cancer incidence in middle-SDI countries.

A

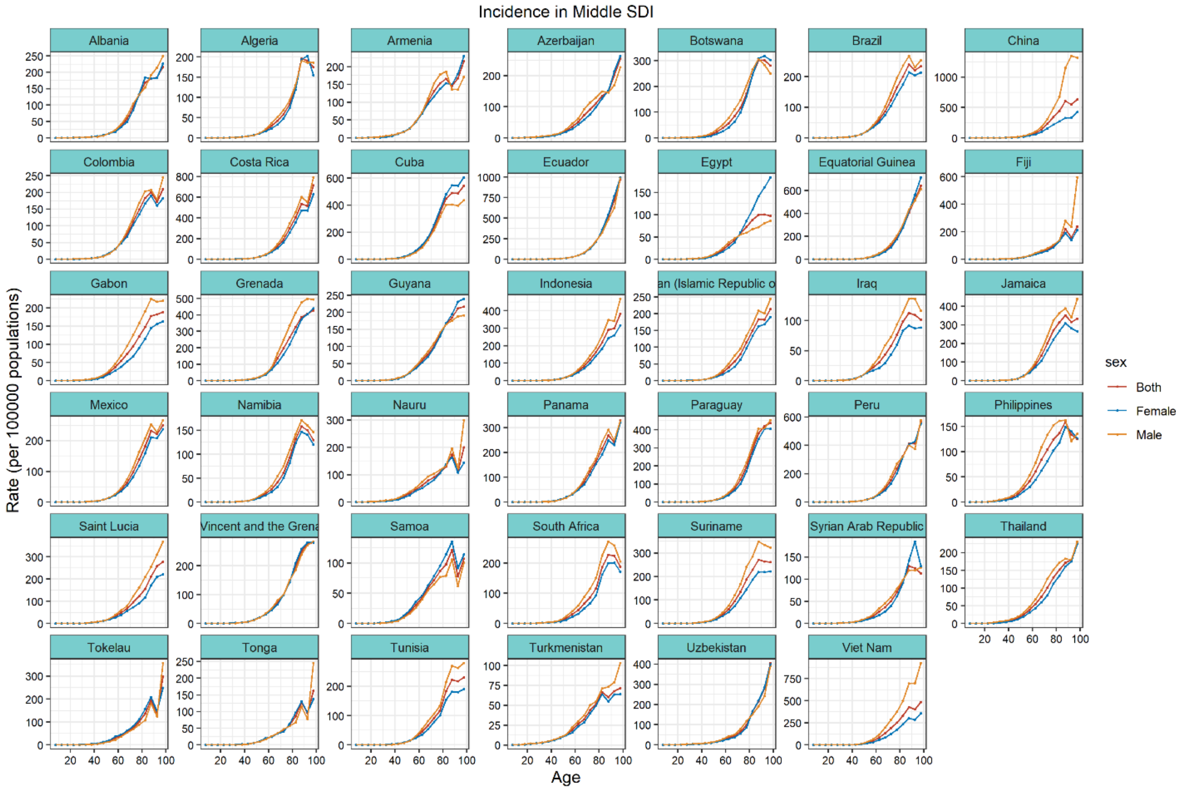

B

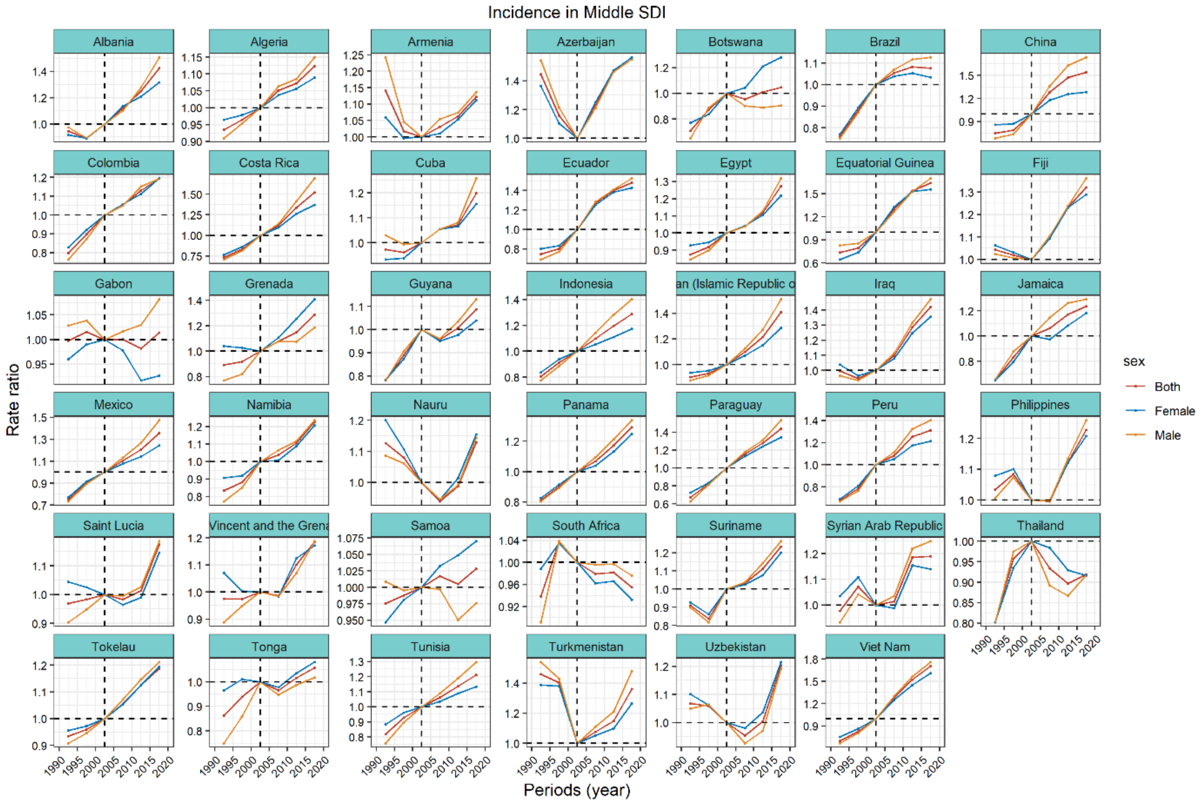

C

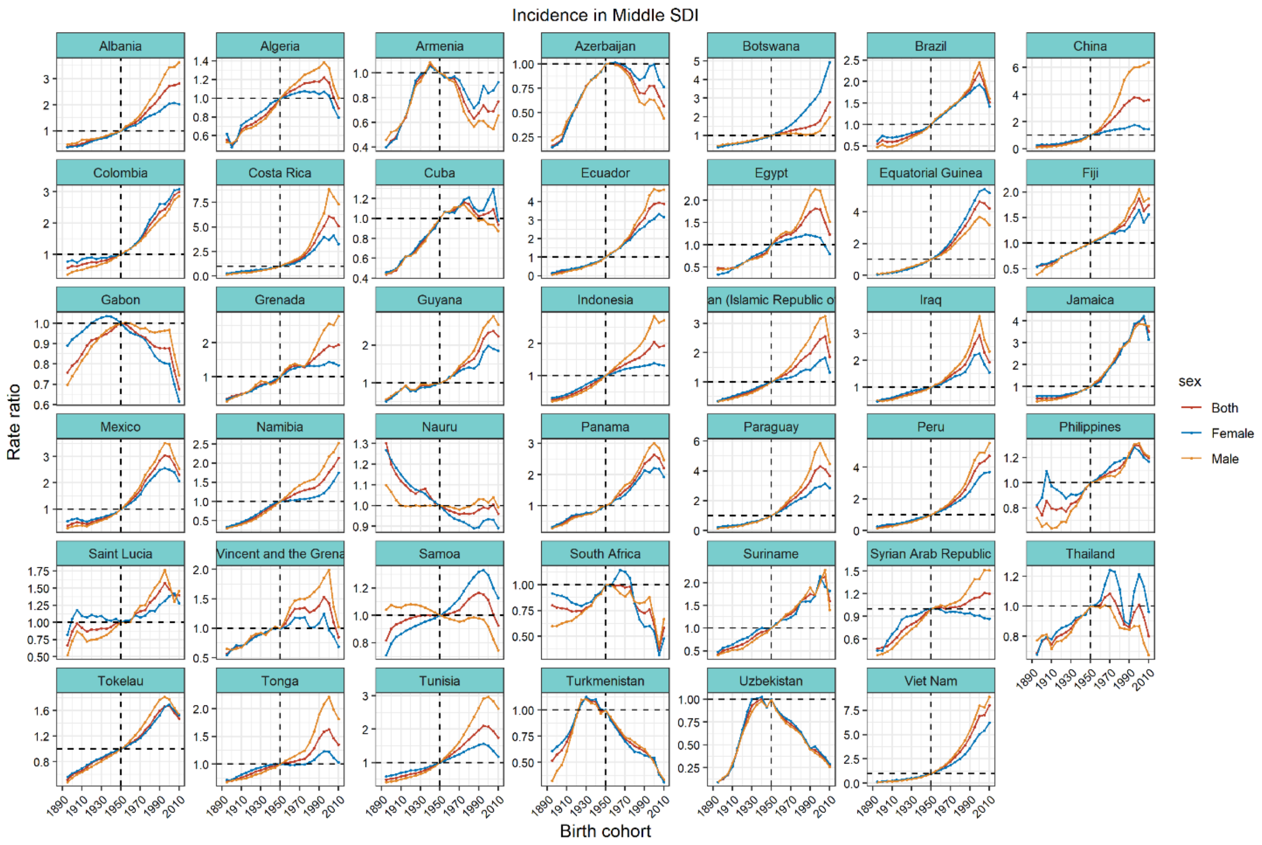

(A) Age effects are shown by the fitted longitudinal age curves of incidence (per 100,000 person-years) adjusted for period deviations.

(B) Period effects are shown by the relative risk of incidence (incidence rate ratio) and computed as the ratio of age-specific rates from 1990−1994 to 2015−2019 (2000−2005 as the referent period).

(C)Cohort effects are shown by the relative risk of incidence and computed as the ratio of age-specific rates from the 1895 cohort to the 2010 cohort, with the referent cohort set at 1950.

FigureS17. Incidence rates of colorectal cancer across different age groups, periods and birth cohorts in low-middle-SDI countries during 1990-2019.

A

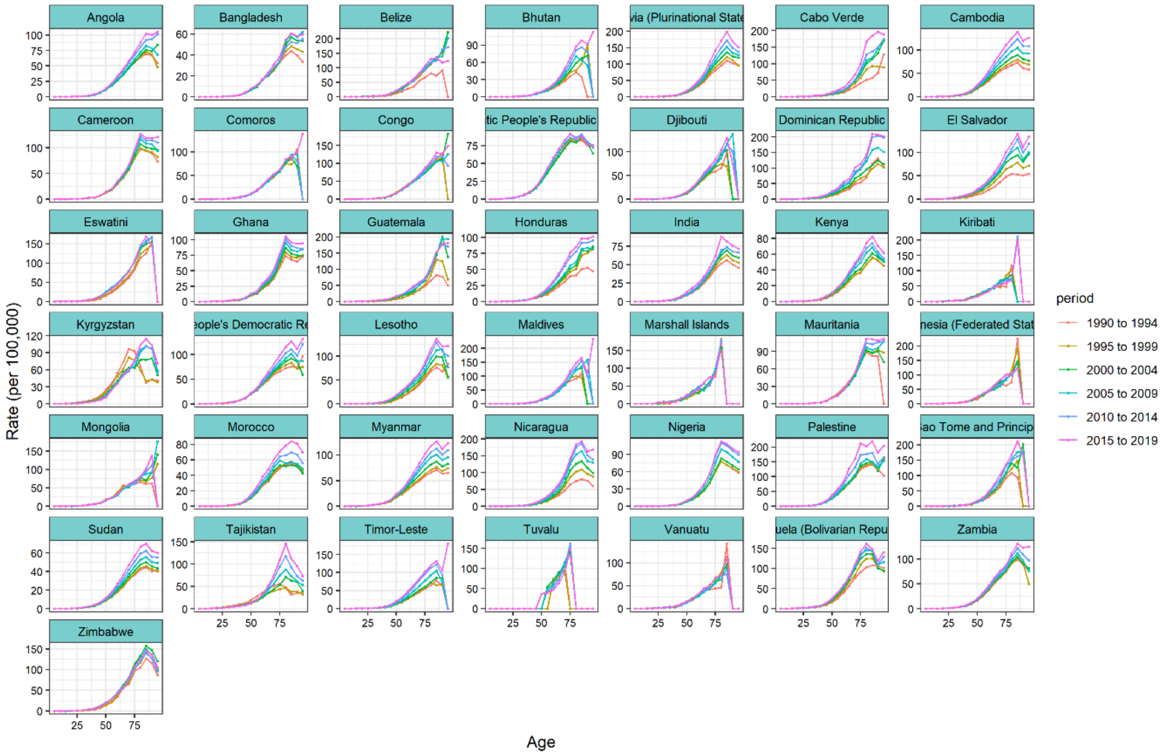

B

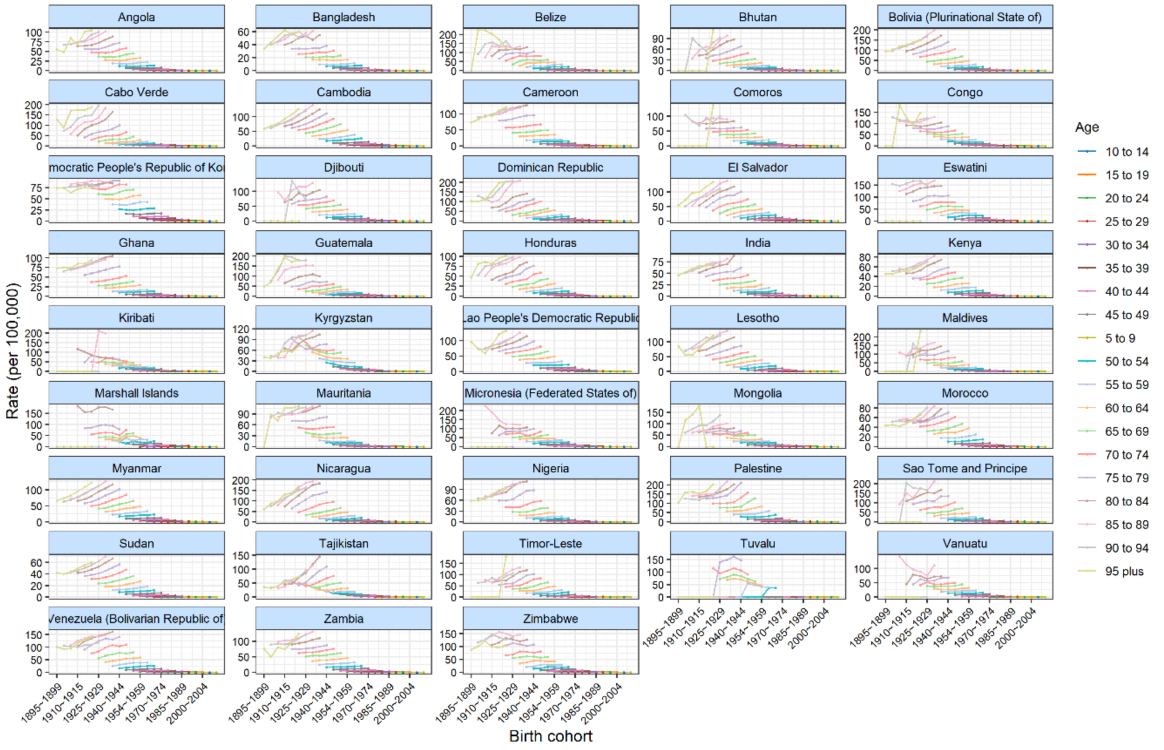

C

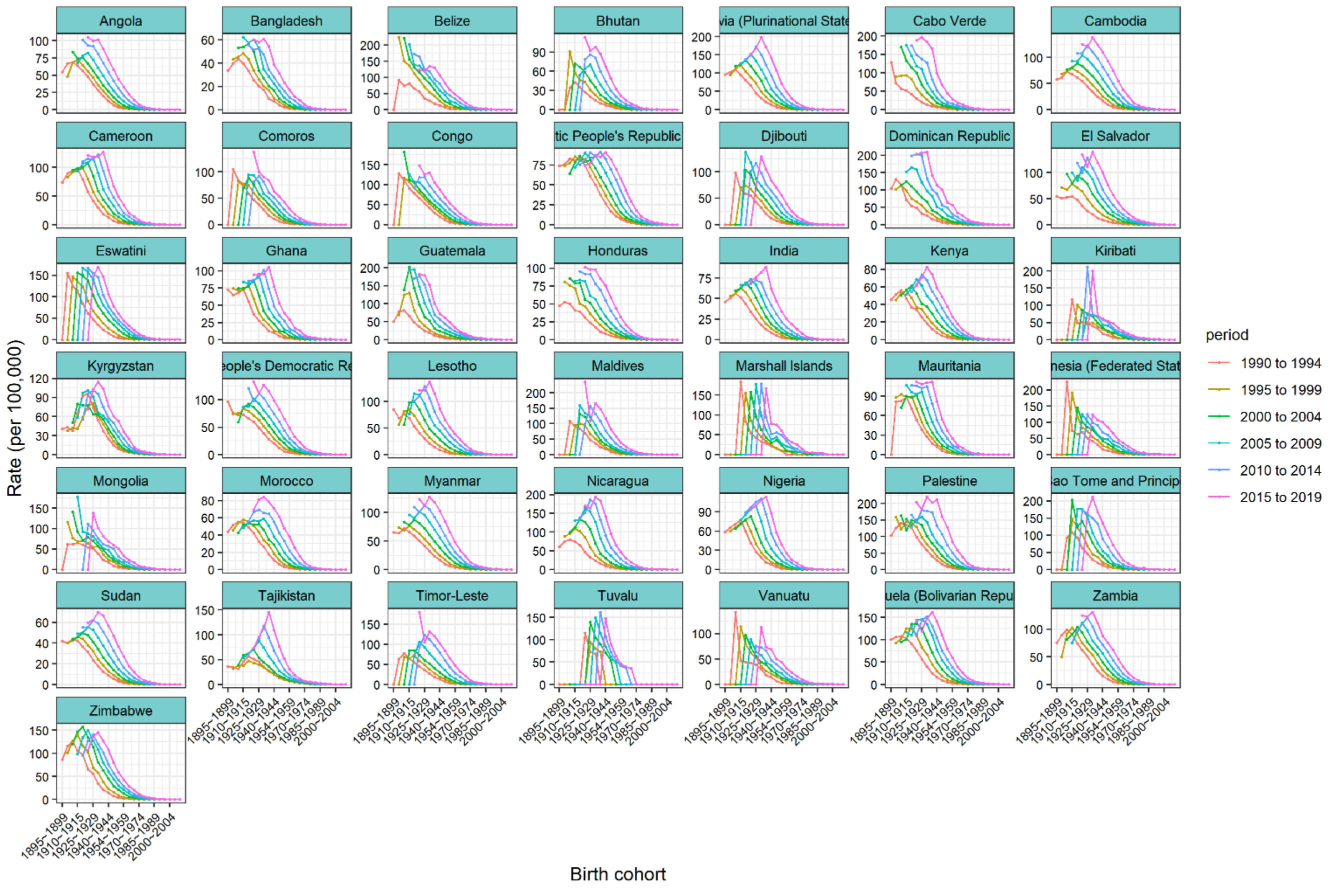

(A) Incidence rates of colorectal cancer across different by periods in low-middle-SDI countries, 1990-2019.

(B) Incidence rates of colorectal cancer across different birth cohorts by age groups in low-middle-SDI countries, 1990-2019.

(C) Incidence rates of colorectal cancer across different birth cohorts by periods in low-middle-SDI countries, 1990-2019.

FigureS18. Age-period-cohort effects on colorectal cancer incidence in low-middle-SDI countries.

A

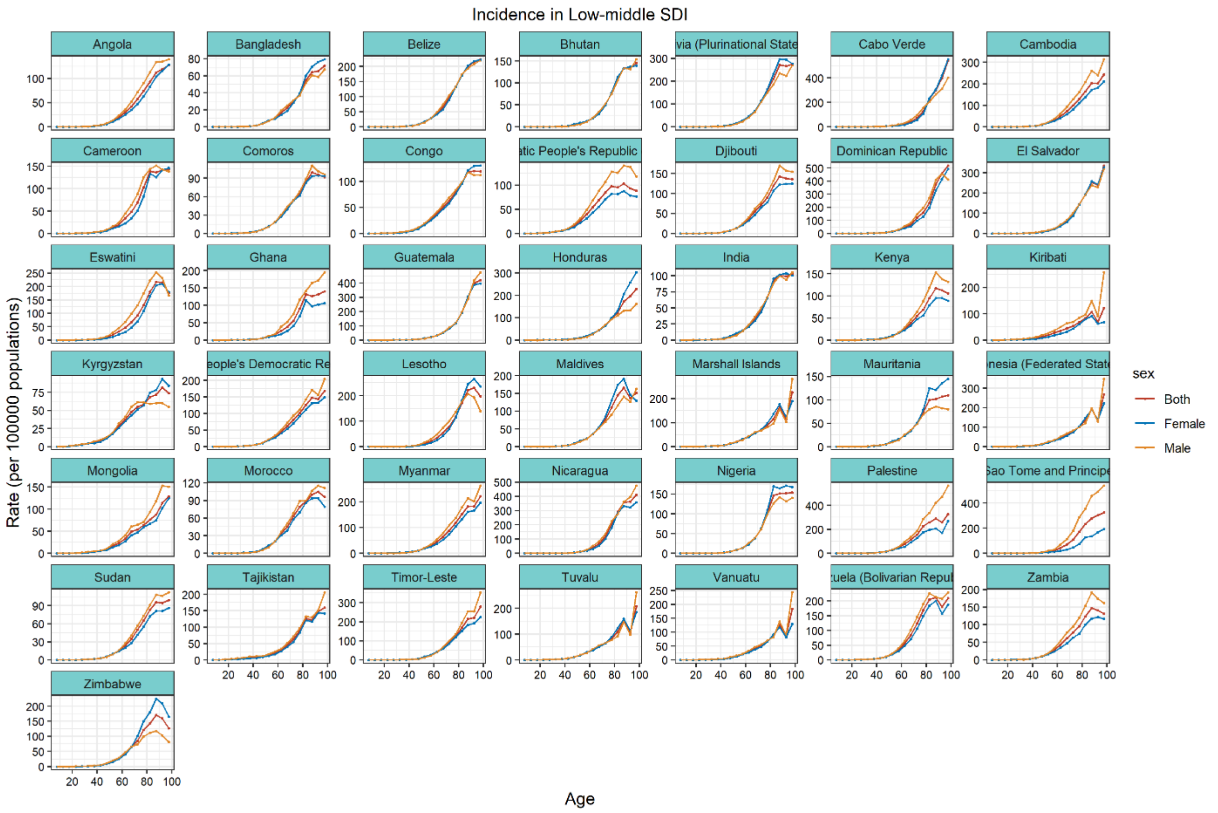

B

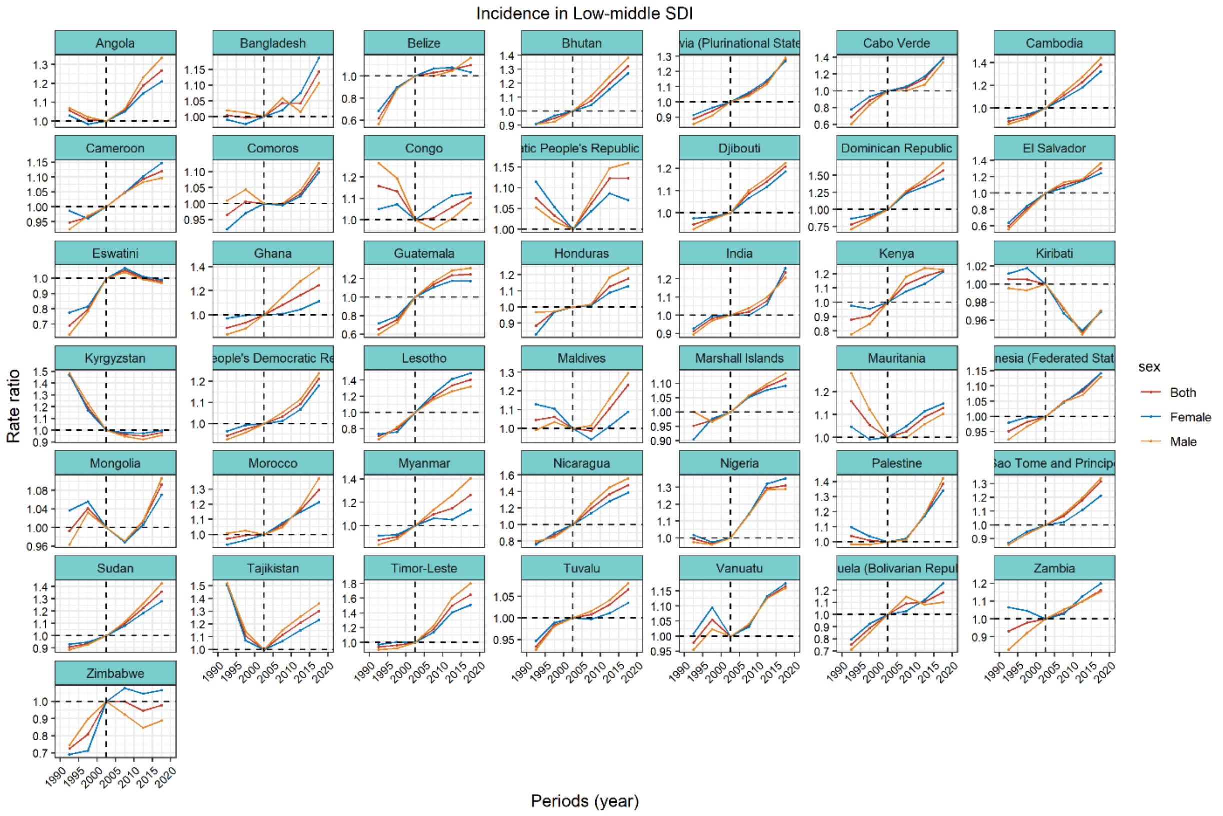

C

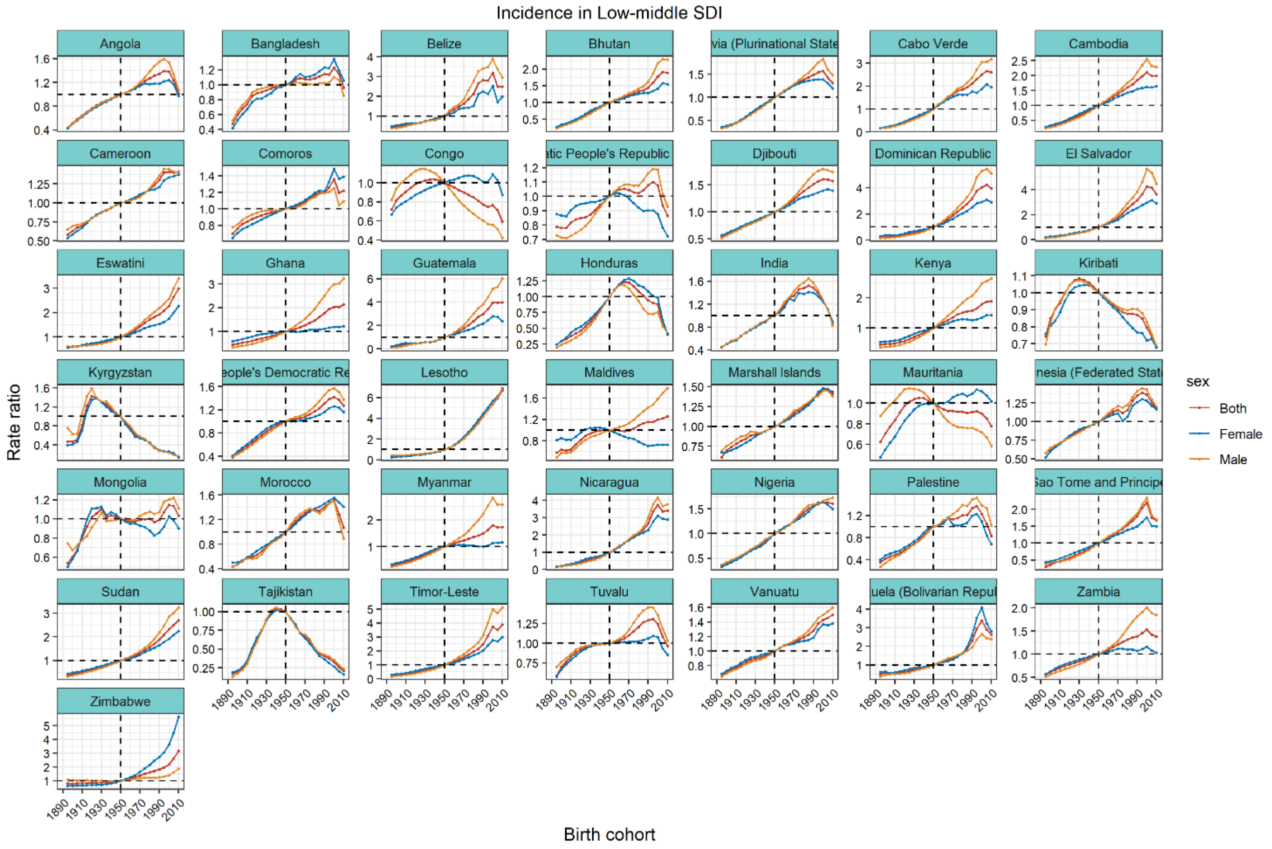

(A) Age effects are shown by the fitted longitudinal age curves of incidence (per 100,000 person-years) adjusted for period deviations.

(B) Period effects are shown by the relative risk of incidence (incidence rate ratio) and computed as the ratio of age-specific rates from 1990−1994 to 2015−2019 (2000−2005 as the referent period).

(C)Cohort effects are shown by the relative risk of incidence and computed as the ratio of age-specific rates from the 1895 cohort to the 2010 cohort, with the referent cohort set at 1950.

FigureS19. Incidence rates of colorectal cancer across different age groups, periods and birth cohorts in low-SDI countries during 1990-2019.

A

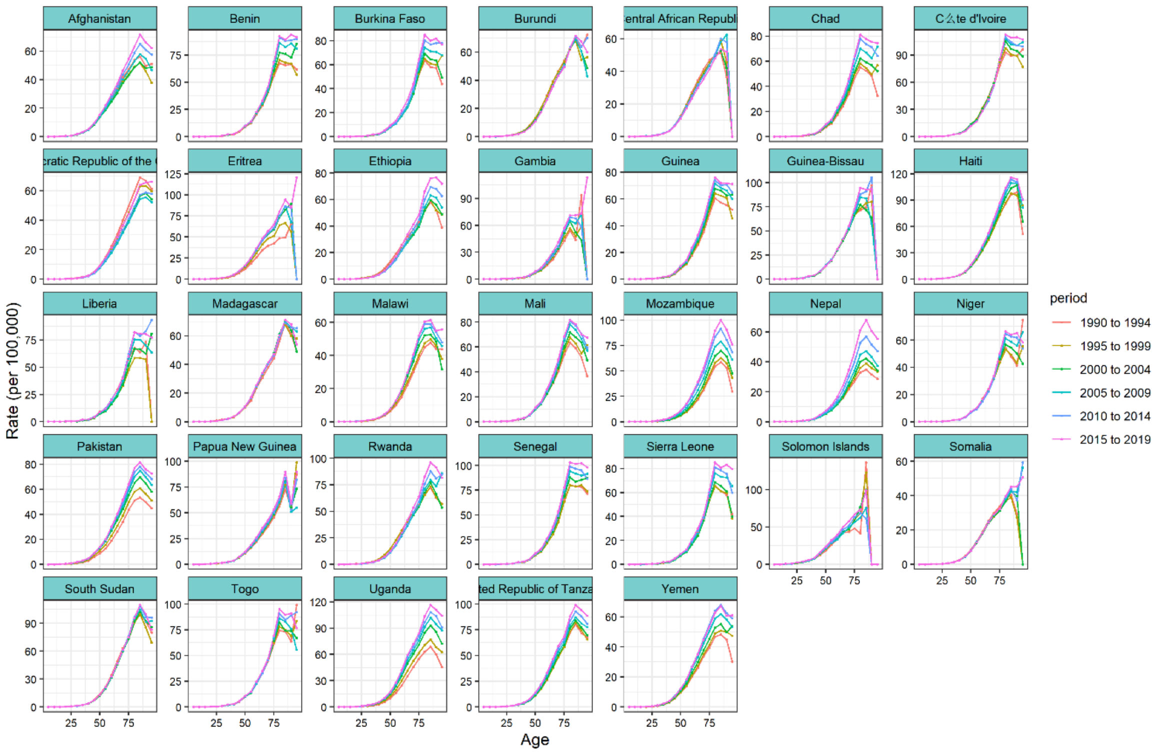

B

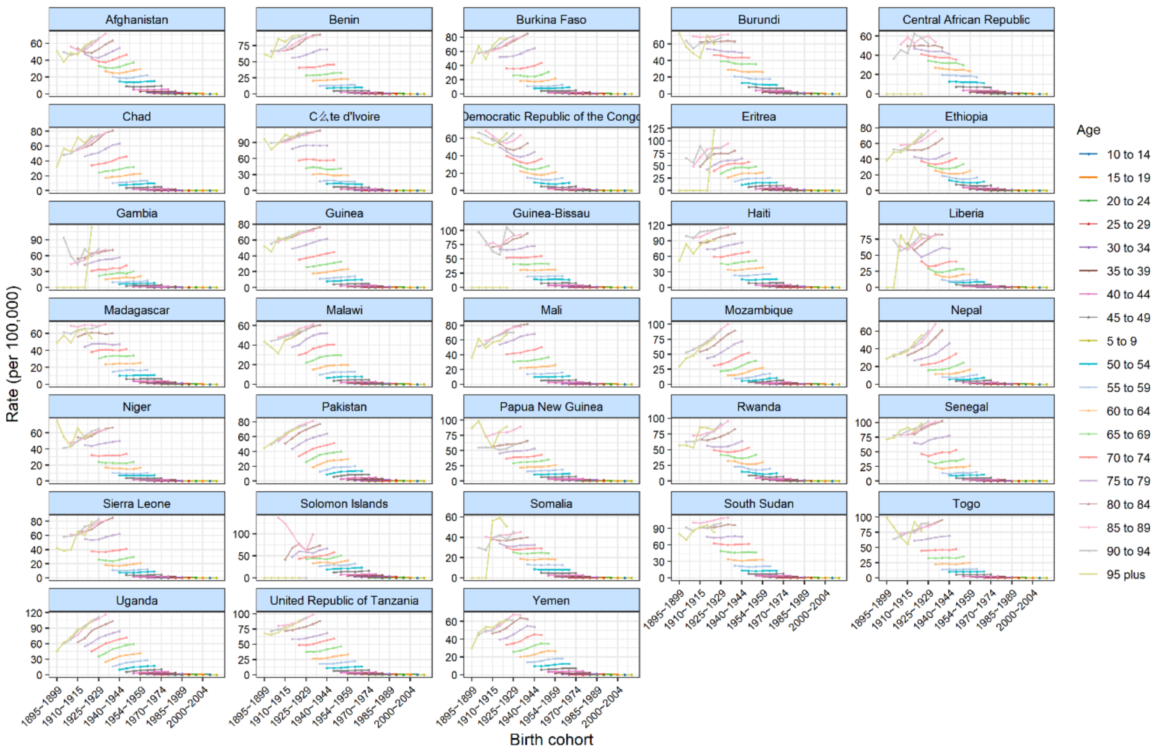

C

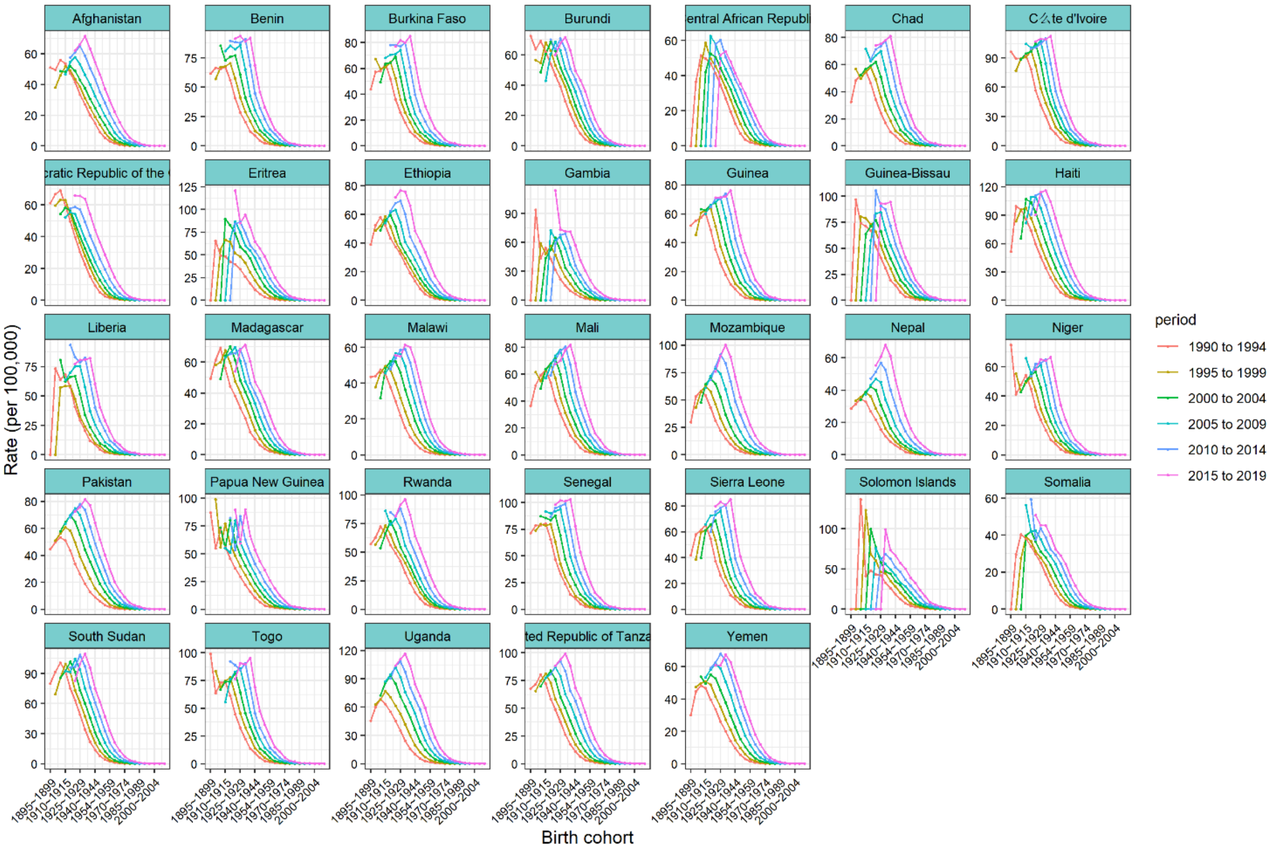

(A) Incidence rates of colorectal cancer across different by periods in low-SDI countries, 1990-2019.

(B) Incidence rates of colorectal cancer across different birth cohorts by age groups in low-SDI countries, 1990-2019.

(C) Incidence rates of colorectal cancer across different birth cohorts by periods in low-SDI countries, 1990-2019.

FigureS20. Age-period-cohort effects on colorectal cancer incidence in low-SDI countries.

A

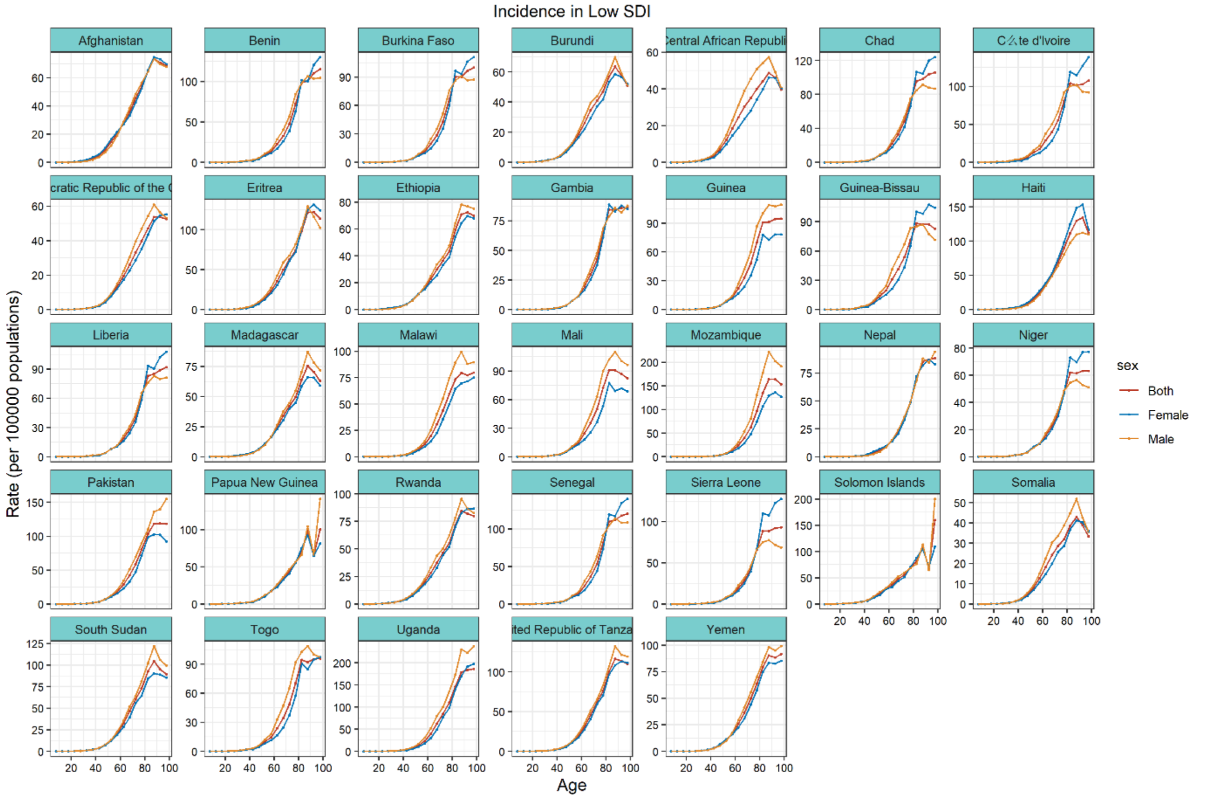

B

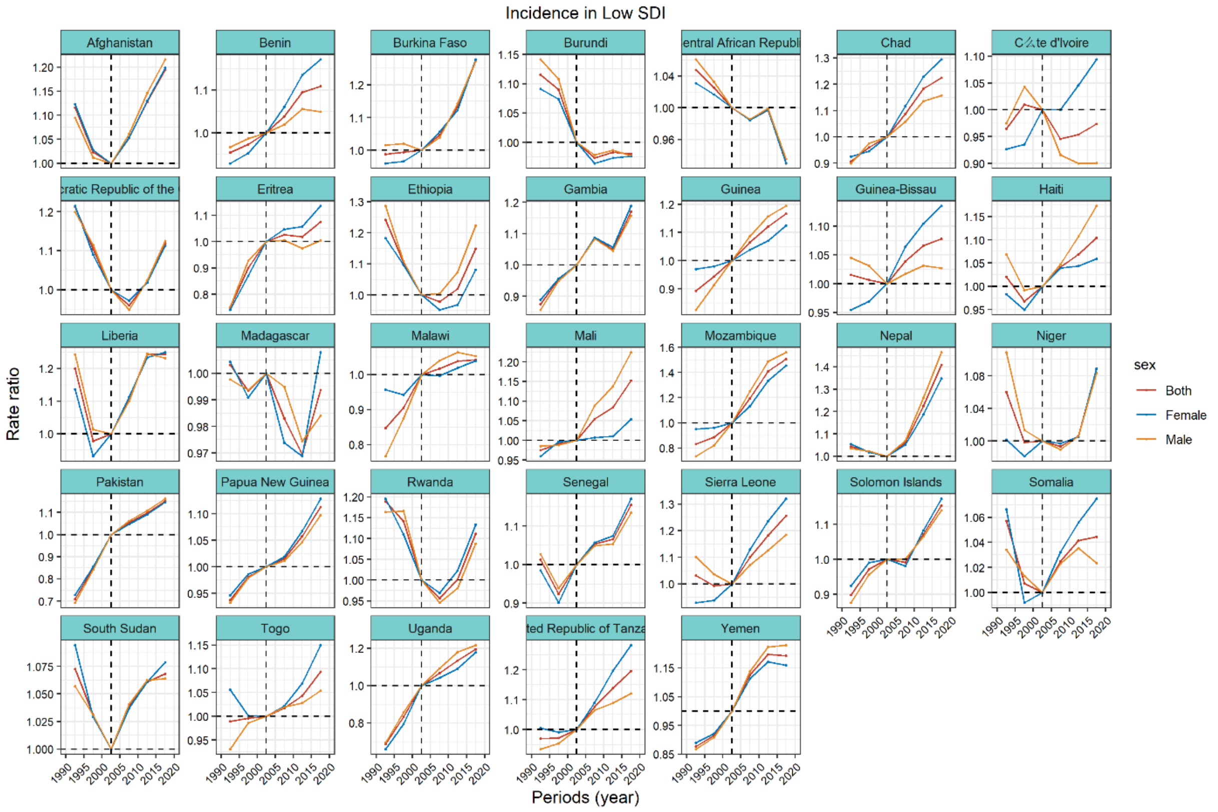

C

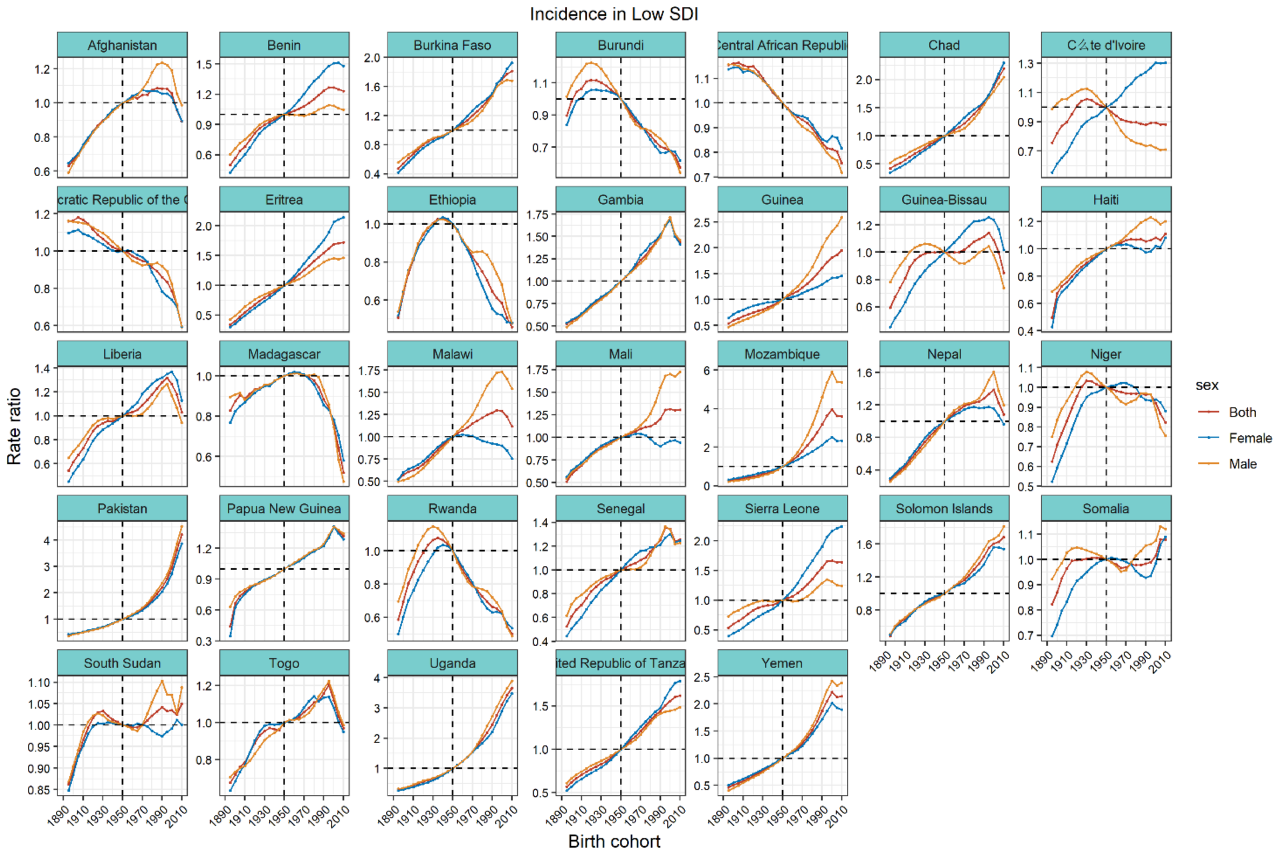

(A) Age effects are shown by the fitted longitudinal age curves of incidence (per 100,000 person-years) adjusted for period deviations.

(B) Period effects are shown by the relative risk of incidence (incidence rate ratio) and computed as the ratio of age-specific rates from 1990−1994 to 2015−2019 (2000−2005 as the referent period).

(C)Cohort effects are shown by the relative risk of incidence and computed as the ratio of age-specific rates from the 1895 cohort to the 2010 cohort, with the referent cohort set at 1950.
